# Supplementary material for: A multi-level classification based ensemble and feature extractor for credit risk assessment
Source: PeerJ Comput Sci. 2024 Feb 29;10:e1915. doi: 10.7717/peerj-cs.1915 (PMC10909241; doi:10.7717/peerj-cs.1915)
Supplement: Supplemental Information 1 — Model training results and comparison data. [file peerj-cs-10-1915-s001.zip › latex/peerj_NEW.pdf]

Individual Credit Risk Assessment Based on an Encoder-FE+GDBT Model

Yuanyuan Wang[1], Zhuang Wu[2], Jing Gao[3], Chenjun Liu[4], and Fangfang Guo[4]

[1]**Capital University of Economics and Business, BeiJing, China**

Corresponding author:
Zhuang Wu[2]

Email address: wuzhuang@cueb.edu.cn

ABSTRACT

With the growth of people's demand for loans, the requirement of banks for personal credit risk is to improve the accuracy of the initial credit risk level of new users. This article is based on individual Internet loan data from 2015 to 2017, proposes a mixed credit risk model, and discusses the three sampling methods dealing with unbalanced data influence on feature extraction and Ensemble learning method. Random Forest, XGBoost, LightGBM and GDBT are selected for training, the Stacking performance of LightGBM and GDBT is better. Feature extraction is used to further optimize the model after Stacking effect, the results show that the hybrid model of credit risk encoder FE + GDBT works better. The determination of initial credit rating for bank personal provide reference and lending decisions.

Keywords: hybrid credit risk model, feature extraction, SMOTE Tomek sampling, Stacking

Subjects: Data Mining and Machine Learning, Neural Networks and Optimization Theory and Computation

INTRODUCTION

We introduce a novel class of hybrid credit risk models for personal credit risk rating, combination of feature extractors and Ensemble learning models. At present, the electronic loan business is already widely used in China. But, the traditional personal credit data, which the traditional credit information has obvious defects, is not suitable for the status quo. The personal credit data set of this study is more suitable for electronic loans. It contains a variety of authentication information, which is convenient for cleaning and processing and conducive to subsequent work. Now, the deep learning model has rarely been used as a feature extraction tool in the field of personal credit risk assessment. The application of Ensemble learning tends to focus on the expansion of a single model, lack of horizontal comparison between different base learners of Ensemble learning. There are few studies on the combination of deep learning and integrated learning.

The contributions of this paper are as follows:

1. Three sampling methods that Random under-sampling(RU), SMOTE Tomek sampling(ST), and Random over-sampling(RO) deal with unbalanced data. The features selected in the paper based on the basic information of personal credit set are more suitable for the current electronic credit loan business, including mobile phone authentication, household registration authentication, video authentication, education certification, credit investigation authentication, and Taobao.com authentication (Taobao.com.com is the Asia-Pacific region's larger network retail, business circle). The learning of these features can better determine the initial level of individual credit risk, and provide certain support for the current credit rating evaluation of financial institutions, credit investigation agencies and other rating agencies.

2. Although the performance results of the feature extractors are not satisfactory, Encoder-FE has great performance. Encoder that with special structure of deep neural network can better learn features. It

not only reflects the powerful learning ability of the deep model, but also provides ideas for learning the features of personal credit information.

3. For the base learners of Ensemble learning, the best result of training set is GDBT that accuracy and loss by ST are 90.54% and 0.3199. After Stacking, there is a special result that needs to be explained: the loss value of the test set by RU is not decreased, and the effect is not as good as the results of the basic model (GDBT). This shows that Stacking is not all good.

4. We propose a hybrid credit risk model, which includes feature extractors and Ensemble learning models. Experimental results show that the loss reduces from 0.2151 to 0.2133 and from 0.2695 to 0.2648 in training and test set by ST; the accuracy increases from 92.41% to 92.58%, and the loss reduces from 0.2683 to 0.2553 in test set by RO. The best performing hybrid credit risk model is Encoder-FE+GDBT model

The remainder of the paper is organized as follows: Section 2 summarizes the related work; Section 3 Data sampling processing and features selection, as well as evaluation criteria; Section 4 features extractors design and construction, base learners of Ensemble learning selection, and hybrid credit risk model; Section 5 reports the experimental results and discussion. Finally, Section 6 concludes the proposed model, and presents several aspects of future work.

RELATED WORK

Personal credit risk assessment is a hot and sensitive topic in the financial industry which identify the credit rating of the new loan customer and whether to make the loan. Personal credit rating helps to make crucial decisions to lend some loan to the applicant or not. Thus, we proposed the hybrid credit risk model in the paper, which used as an auxiliary tool to help researchers and the financial industry distinguish between risky customers and non-risky customers. Throughout the history of credit risk measurement, its development process has experienced the expert subjective judgment method, statistical method, and then to the traditional machine learning method, and now is the modern credit risk assessment model based on artificial intelligence, credit risk measurement has been continuously developed and improved.

For the expert subjective judgment method, credit applicants submit written certification materials, and experts often use 5C element analysis method and 5W element analysis method according to their experience to make subjective judgments on credit decisions, which is difficult to ensure fairness. Statistical methods emerged and developed to address subjective influences, including Multivariate analysis Zhou et al. (2010); De Andres et al. (2011); Finlay (2011); Yeh and Lien (2009), Dependent Variable Limited Lessmann and Voß (2009); Lin (2009); Wang et al. (2011); Zambaldi et al. (2011),Dong et al. (2010); Tsai and Chen (2010), Probabilistic MethodsPsillaki et al. (2010),Tong et al. (2012), Non-Linear Regression Louzis et al. (2012); Ghosh (2015), Linear Regression Li et al. (2011), Non-Parametric Statistics Tsai and Chen (2010); Malik and Thomas (2010), Sampling TechniquesSun et al. (2018); Xia et al. (2017b), Multiple Criteria Decision MakingPeng et al. (2011); Zhu et al. (2013); Kruppa et al. (2013); AF Ferreira et al. (2014), etc. With the development of computer technology, machine learning comes into people's view. Some commonly used machine learning (ML) techniques are decision tree (DT) Zhu et al. (2013), k-nearest neighbors (KNN), support vector machine (SVM) Lessmann and Voß (2009) and Naïve Bayes (NB) Hsieh and Hung (2010). It is difficult for a single machine learning algorithm to comprehensively guarantee the best result in every case, so we start to consider from multiple aspects and conduct the combination of multiple machine learning models and ensemble learning exploration.

In this paper, three aspects are summarized:

Sampling methods: The personal credit rating in this paper is a multi-classification problem. The number of users at each level is not equal, and the number of customers with "good" credit is more than that of "bad", indicating that the data set is lack of balance. Unbalanced data is one of the common problems in credit rating datasets. The commonly used sampling methods include Random under-sampling (RU), Random over-sampling (RO) and Synthetic minority oversampling technique (SMOTE). RO, taking samples randomly from categories with few samples, and then adding the sampled samples to the data set. Because repeated sampling often leads to severe overfitting, it is now rarely used in machine learning. RU is similar, randomly taking a small number of the same number of samples. Its defect is to sample the samples of the least category as the standard. Too small number of the least category will lead to insufficient number of final samples. The prevailing oversampling method now is to achieve class balance

by synthesizing some minority samples somehow, and one of these is SMOTE. In summary, SMOTE's idea was to interpolate between a few class samples to produce additional samples. The SMOTE achieves optimized performance by oversampling the minority class samples Chawla et al. (2002).For sampling methods related research, Yu et al. (2018) propose a DBN based over-sampling SVM ensemble learning paradigm to solve imbalanced data problem in credit classification. The experimental results indicate that the classification performance are improved effectively when the DBN-based ensemble strategy is integrated with over-sampling techniques. Mirzaei et al. (2020) present an effective under-sampling technique to select the suitable samples of majority class using the DBSCAN algorithm. The results of balancing training sets show that this method is superior to other 6 pretreatment methods. Guzmán-Ponce et al. (2021) propose a two-stage under-sampling technique that combines the DBSCAN and a minimum spanning tree algorithm, thus handling class overlap and imbalance simultaneously with the aim of improving the performance of classifiers. Sun et al. (2018) proposes a new DT ensemble model for imbalanced enterprise credit evaluation based on the SMOTE and the Bagging ensemble learning algorithm with differentiated sampling rates (DSR), which is named as DTE-SBD. It can not only dispose the class imbalance problem of enterprise credit evaluation, but also increase the diversity of base classifiers for DT ensemble. Xia et al. (2017b) Two real-world P2P lending datasets are examined. Among, CSLR-SMOTE and CSRF-SMOTE methods are used; Experimental results reveal that the proposed loan evaluation and portfolio allocation model are the best performing methods. The above studies indicate that the application of sampling methods can be used as a promising tool for credit risk classification of unbalanced data. In order to deal with unbalanced data and compare the performance of various sampling methods, RU, RO and ST methods are applied in this paper. SMOTE Tomek sampling (ST), a comprehensive sampling method, combines SMOTE and Tomek Links methods. Tomek Link can "clean out" the overlapping samples between classes, so that the samples that are closest to each other belong to the same category, which allows for better classification.

Feature extraction methods: Machine learning and ensemble learning can be further enhanced by implementing certain preprocessing mechanisms, such as feature extraction (FE) and resampling the instances. For feature extraction methods related research, Chen et al. (2009) selected conventional statistical LDA, Decision tree, Rough sets and F-score approaches as features extraction, and combined with support vector machine (SVM) classifier to construct different credit scoring models. Feature extraction can better classify by removing irrelevant and redundant features. Oreski and Oreski (2014) proposed the hybrid genetic algorithm with neural networks (HGA-NN), which is used to identify an optimum feature subset and to increase the classification accuracy and scalability in credit risk assessment. The feature extraction methods are t-test, correlation matrix, stepwise, regression, PCA, and factor analysis. Dahiya et al. (2017) used GA and ANN to select the optimal features improve the accuracy and stability of the credit scoring model. Lenka et al. (2022) employed to identify the informative features, which help to reduce the models? dimensionality and complexity. It implements three feature extraction techniques, i.e., IG, PCA, and GA, to select the relevant features.

Ensemble learning methods: Wang and Ma (2012) propose a hybrid ensemble approach (RSB-SVM), which is based on bagging and random subspace, and use Support Vector Machine (SVM) as base learner. Experimental results reveal that RSB-SVM can be used as an alternative method for enterprise credit risk assessment. Abellán and Castellano (2017) extend a previous work about the selection of the best base classifier used in ensembles on credit data sets, and prove that a classifier is the key point to be selected for an ensemble scheme. Xia et al. (2017a) propose a sequential ensemble credit scoring model based on XGBoost, and provide feature importance scores and decision chart, which enhance the interpretability of credit scoring model. Xia et al. (2018) propose a novel heterogeneous ensemble credit model that integrates the bagging algorithm with the stacking method, and verify the validity of the method.

Improving the performance of the Ensemble learning model can be achieved with a single base learner with different variants or with a combination of different base learners.In order to improve the generalization ability and robustness of the Ensemble learning model, it is necessary to pay attention to the diversity and performance of the base learner. Diversified base learners enhance the performance of the Ensemble learning model Lenka et al. (2022). Bagging Kearns et al. (1992) and Boosting Abellán and Castellano (2017); Pławiak et al. (2020); Arora and Kaur (2020); Khashman (2010) are two common methods for generating multiple subsets. Combined output methods include voting (Supermajority voting, Relative majority voting, and Weighted voting), weighted average, and stacking Tsai et al. (2014); Behr and Weinblat (2017) , etc. Therefore, the base learners of the paper including Random forest and GDBT

belong to bagging, and including XGBoost and LightGBM belong to Boosting.The construction of Ensemble learning model includes the creation of different base learner and the combination of base learning output. The commonly used stacking method with better effect is selected in this paper.

DATA PROCESSING

Data features

In terms of data cleaning, we delete the missing data or lost data. In addition to the initial rating of the target feature (Initial rating list credit rating at the time of transaction), there are 19 features. Table 1 shows these features and description.

Table 1. Data features description

No.	Features	Features meaning
1	Loan amount	Total transaction amount
2	Borrowing term	The total number of the loan term (in months)
3	Borrowing rate	Annualized interest rate (percent)
4	Initial rating list credit rating at the time of transaction	A to F are credit ratings
5	Borrowing type	The types of loans are divided into ‘Ecommerce’, ‘APP’, ‘Ordinary’, and ‘Other’
6	First bid	Whether the bid is the first bid of the borrower
7	Age	The age of the borrower at which the list was successfully borrowed
8	Gender	The list borrower gender
9	Mobile phone authentication	This list indicates whether the borrower’s mobile phone real-name authentication is successful
10	Account authentication	Indicates whether the account authentication of the list borrower is successful
11	Video authentication	This list indicates whether the video authentication of the borrower is successful
12	Education certification	Whether the list of borrowers has been successfully certified. Success means a college degree or above
13	Credit reference authentication	The list of borrowers? credit reference authentication is successful. Success means having a credit report online
14	Taobao.com certification	This list of borrowers? Taobao.com certifications is successful. Success is expressed as a Taobao.com shop owner
15	Historical number of successful loans	The number of successful loans a borrower borrowed before the list closed
16	Historical amount of successful borrowing	The amount of successful borrowing by the borrower before the closing of the list
17	History always needs to be repaid	The amount of principal to be repaid by the borrower before the closing of the list
18	Historical Normal Repayment Maturities	The number of repayment maturities of the borrower before the closing of the list
19	Historical delinquencies	The number of delinquencies of the borrower before the closing of the list

In Table 1, the selections of the features, including mobile phone authentication, registration certification, video certification, credit certification, credit reference authentication, Taobao.com certification, etc., which more suitable for the current electronic credit features. The certifications can not only prove the identity of the current customer can also win at a greater extent related to customer credit information, and the success of the certification, to a certain extent, it can prove the level of customer credit risk.

On the basis of the original data features, two features are added, namely, the proportion of historical normal repayment times and the proportion of historical overdue repayment times. These two features can directly represent the customer’s repayment attitude.

Assuming, Number of successful loans in history is $H\_T(i)$, borrowing term is $H\_M(i)$ (value takes mode equal to 12), the historical normal repayment periods is $H\_N(i)$ and the historical number of late payments is $H\_O(i)$. The Formula 1 shows the proportion of normal repayment times ($P\_N(i)$), and the formula of the ratio of overdue repayment times ($P\_O(i)$) is shown in Formula 2 .

$$P\_N(i) = \frac{H\_N(i)}{H\_T(i) \times H\_M(i)} \tag{1}$$

$$P\_O(i) = \frac{H\_O(i)}{H\_T(i) \times H\_M(i)} \tag{2}$$

Symbols and characteristic description of all features ($X1$-$X20$) and target features (Y) are described in Table 2.

Table 2. Features and symbols description

Symbol	Features	Symbol	Features
Y	Loan amount	$X11$	Education certification
$X1$	Borrowing term	$X12$	Credit reference authentication
$X2$	Borrowing rate	$X13$	Taobao.com certification
$X3$	Initial rating list credit rating at the time of transaction	$X14$	Historical number of successful loans
$X4$	Borrowing type	$X15$	Historical amount of successful borrowing
$X5$	First bid	$X16$	History always needs to be repaid
$X6$	Age	$X17$	Historical Normal Repayment Maturities
$X7$	Gender	$X18$	Historical delinquencies
$X8$	Mobile phone authentication	$X19$	The proportion of historical normal repayment times
$X9$	Account authentication	$X20$	The proportion of historical overdue repayment times
$X10$	Video authentication		

The Y represents the dependent variable of the objective function, $X1$-$X20$ represents the independent variables affecting Y. The reason for this is to conduct the following principal component analysis, rather than taking the initial grade feature ($X3$) of the study focus as the objective function.

Data sampling

First of all, delete lost data. Next, the data is randomly divided into training set and test set in the ratio of 8:2, which were used to training the set and test set the generalization ability of the model. The $X3$ (Initial rating list credit rating at the time of transaction) situation of the training set and the test set is counted, as shown in Table 3.

Table 3. Individual initial credit rating data distribution

$X3$	Training set	Test set
A	765	190
B	2281	6601
C	19202	4803
D	15168	3816
E	2370	547
F	276	83

From Table 3, the $X3$, which the proportion of A-F, is typical unbalanced data. In this study, the assessment of initial personal credit rating is a classification problem. If algorithm training is used directly for classification, the training effect may be poor. Therefore, it requires the sampling of unbalanced data. In this paper, Random under-sampling(RU), SMOTE Tomek sampling(ST), and Random over-sampling(RO) are selected to sample the training set and test set.

After processing by the three methods, the changes in the number of samples are shown in Table 4.

After sampling, the amount of data from A to F remains at the same level, in other words, the amount is equal or similar, as shown in Table 4. This indicates that the processed samples are balanced data, which is convenient for subsequent training.

Table 4. Individual initial credit rating data distribution after processing

$X3$	A	B	C	D	E	F
RO Training set	19201	19201	19201	19201	19201	19201
RO Test set	4803	4803	4803	4803	4803	4803
RU Training set	276	276	276	276	276	276
RU Test set	83	83	83	83	83	83
ST Training set	18333	17860	14917	15353	17727	18741
ST Test set	4549	4412	3674	3776	4394	4632

Evaluation criteria

The assessment of personal credit risk in this paper is a multi-classification problem, the predicted initial grade results need to be classified. It is necessary to classify the predicted initial grade results into six categories.

Log loss function for multiple classes, loss function corresponding to Softmax classifier. The main difference between sigmoid and Softmax is that sigmoid is used for binary classification while Softmax is used for multiple classification. The calculating process of Softmax is shown in Formula 3.

$$S_j = \frac{e^{a_j}}{\sum_{k=1}^{T} e^{a_k}} \tag{3}$$

Assuming, the input sample of Softmax is I, a T classification problem is discussed, that is, I is a vector of $T \times 1$, then a_j in the Formula 3 represents the jth value in the vector of $T \times 1$. And a_k in the denominator is the all T values in the vector $T \times 1$.

The calculating process of Softmax loss is shown in Formula 4:

$$L_j = -\sum_{j=1}^{T} y_i log S_j \tag{4}$$

S_j is the jth value of Softmax's output vector S and represents the probability that this sample belongs to the jth category y, which T values only one value is 1 and the other $T-1$ values are 0, is a $T \times 1$ vector.

The calculating process of cross entropy loss is shown in Formula 5:

$$E = -\sum_{j=1}^{T} y_j log P_j \tag{5}$$

In Formula 5, P_j is the jth value of the input probability vector P. When the input P of the cross entropy is the output of the Softmax, the cross entropy is equal to the Softmax loss.

The log loss of multiple classes (categorical cross entropy) is selected as the evaluation index of the training and test set. The calculating process of Loss is shown in Formula 6.

$$Loss = -\frac{1}{n}\sum_{i=1}^{n}\sum_{j=1}^{k} y_{ij} log \hat{y}_{ij} \tag{6}$$

In the Formula 6, n is the number of samples and k is the number of categories. For multi-classification problems, there are as many categories as the output of the model. There is exclusivity between categories.

Loss value is the sum of the all categories, and the smaller - Loss value is, the better.

The other is accuracy. Both the real label and the model prediction are scalars. For example, the real label is [1,2,4,6,3,5], and the prediction of the model output is [1,2,3,6,4,5], at this point accuracy = 4/6. The accuracy calculation formula is as follows Formula 7.

$$Accuracy = \frac{\sum_{j=1}^{M} y_j}{\sum_{i=1}^{N} y_i} \tag{7}$$

In the Formula 7, N is the number of samples and M is the number of correct categories. The y_i is the value of the category label.

HYBRID CREDIT RISK MODEL FRAMEWORK

The paper introduces the framework of hybrid credit risk model, which consists of two parts, including the three feature extractors in the first part and stacking the batter base learners of Ensemble learning in the second part. We also discuss the learning effect of the hybrid models under three sampling conditions.

Feature extraction process

This section describes three kinds of feature extractors, including DNN Feature Extractor (DNN-FE), Encoder Feature Extractor (Encoder-FE) and Principal component analysis (PCA) Feature Extractor (PCA-FE). As a feature extractor, DNN-FE is a deep learning model formed through multiple layers superposition, which can study the impact of deep learning on results; Encoder-FE, Encoder learns features of the hidden layer as input of subsequent model, and studies whether features learned in an unsupervised way can improve the performance of post-integrated learning model; PCA-FE can reduce the dimension of high-dimensional data to contain as much information as possible, making the few features acquired after dimensionality reduction more representative.

DNN Feature Extractor

DNN is a superposition of multiple networks formed as a deep learning model, in which the hidden layer can be a complex set of nonlinear mapping, and the massive abstract transforms the original data, so deep convolutional neural networks can extract richer features.

In the paper, a multi-layer fully connected DNN (sometimes called Multi-Layer perceptron, MLP) is applied. Individual credit risk rating is a multi-classification problem, so the loss function that the multi-classification cross-entropy loss function is chosen for DNN Feature Extractor (DNN-FE). The optimizer selects Adaptive moment estimation(Adam), the advantage of Adam mainly lies in that after bias correction, the learning rate of each iteration has a certain range, which makes the parameters relatively stable, so it is considered to be the preferred optimization algorithm for deep learning at present.

We determine the network structure of DNN by the following steps: First of all, the number of nodes in the input layer of DNN is 20 that equals the number of final selected features, six nodes in the output layer are the result of the multi-classification. Secondly, the experiment is carried out with a half-decreasing structure in every hidden layer, and the selection range of node numbers is 10-100. Finally, the structure of DNN is determined according to the experimental results, which shown in Figure 1, as 20-100-50-20-6(H_1-H_2-H_3-H_4-H_5).

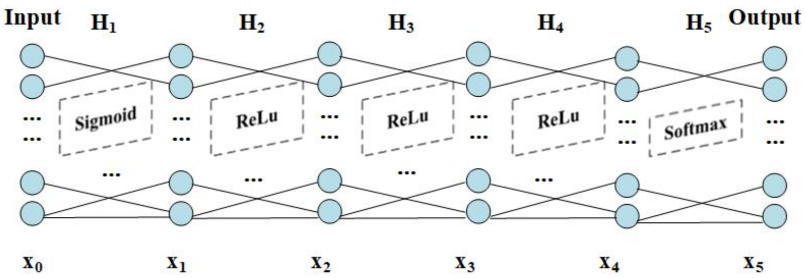

Figure 1. Structure of DNN Feature Extractor

The DNN-FE selects the hidden layers' information as input to the Ensemble learning model. First of all, we train and save the DNN-FE to extract the hidden layers' information. When loading and using it, we need to ensure that the output dimension of DNN-FE is equal to the input dimension of the Ensemble learning model. At that time, we find the layer (H_4) contains 20 nodes (X_4), the number of dimension equals to it. Therefore, we drop the final output layer of DNN-FE, save the information of the current model for inputting the following model. The paper final selects trained result with the new DNN-FE model (H_1-H_2-H_3-H_4), and then input the Ensemble learning model.

To explore and verify the optimal activation functions, the paper experimented with the activation functions commonly used in DNN, mainly observing the comparison of accuracy in training set. The result is shown in Figure 2. The activation functions such as sigmoid, tanh, ReLu, and leaky-ReLu are shown in Figure 3.

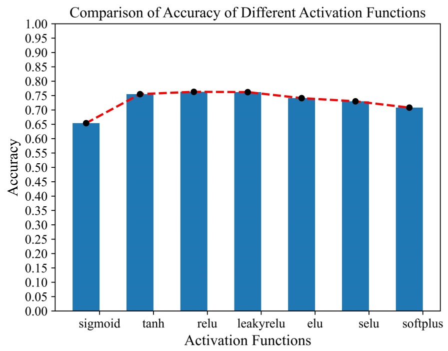

Figure 2. The accuracy for different types of activation functions in training set

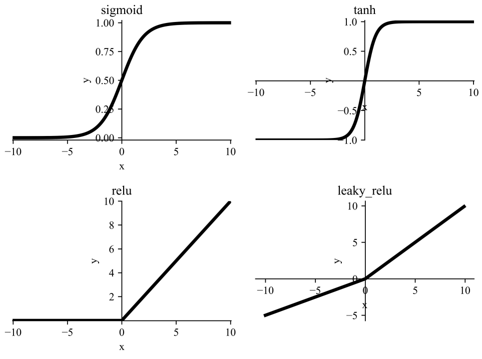

Figure 3. Figures of activation functions

The activation functions have different effects in DNN-FE. The accuracy and loss of various activation functions in training set are compared, and shown in Table 5. The best values of results are in bold.

Table 5. Results of activation functions in training set

Activation Functions	Accuracy	Loss
sigmoid	0.654119178	0.852063407
tanh	0.755061151	0.616983513
ReLU	**0.762972875**	**0.59039235**
Leaky- ReLU	0.76150306	0.595248039
ELU	0.741401764	0.641169026
SELU	0.729924136	0.669819384
SoftPlus	0.707781693	0.719742627

In Table 5 and Figure 2, there is not much difference between the accuracy of the traditional types of activation functions, among which Leaky- ReLU and ReLU classical activation functions perform better; Sigmoid worst performers, in all the traditional types of activation function in training will face the plight of gradient disappeared, lead to cannot further enhance accuracy; ReLU function both in the training set accuracy and loss are significantly better than the other activation function, can greatly enhance convergence speed of the model. In the DNN-FE, the Sigmoid function is selected as the activation function in the input layer later to ensure that the predicted value after is in the range of positive numbers. The ReLu function is selected as the activation function, which can greatly provide accuracy. The Softmax classifier function is used for multi-classification in the output layer. Finally, six types of results are output.

Using the RU method, the results of the accuracy and loss values of DNN-EF, which training 100

epochs in the training and validation set for example, are shown in Figure 4(a). The test and validation set are shown in Figure 4(b).

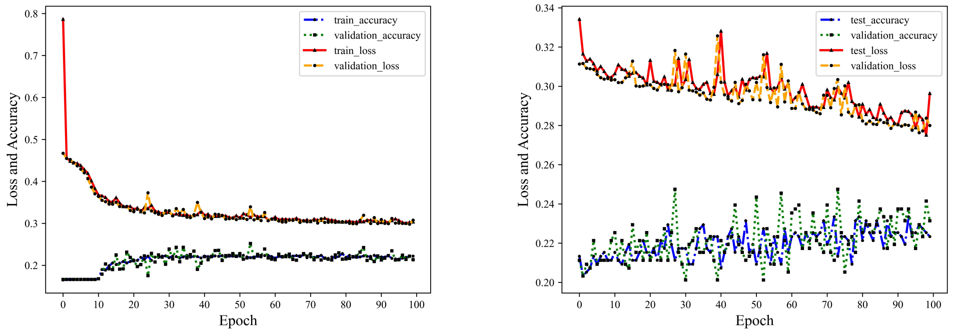

(a) Loss and accuracy of training set by RU (b) Loss and accuracy of test set by RU

Figure 4. Results of training and test set by DNN-FE and RU

In Figure 4(a), loss decreases greatly and rapidly within 10 epochs, ranging from 1.0 to 0.3, with little improvement in accuracy; In Figure 4(b), The decline range of loss is between 0.34 and 0.28, showing a fluctuating decline, the change in accuracy is similar to the training set, from 0.2 to 0.25.

Using the ST method, the accuracy and loss values of results in the training and test set, which training 100 epochs, are shown in Figure 5(a) and 5(b).

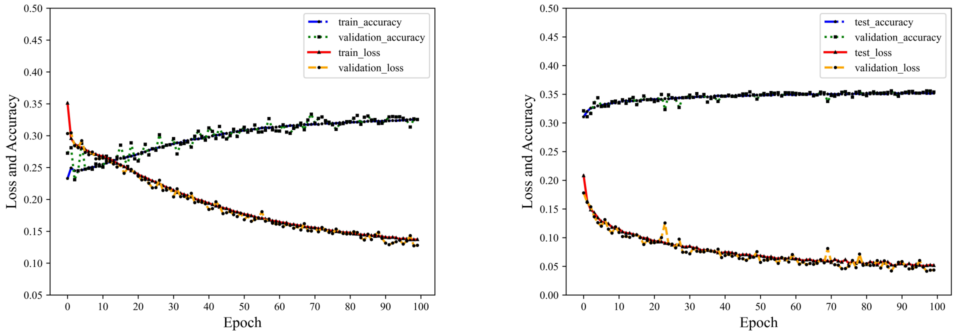

(a) Loss and accuracy of training set by ST (b) Loss and accuracy of test set by ST

Figure 5. Results of training and test set by DNN-FE and ST

In Figure 5(a), loss decreases greatly and rapidly, ranging from 0.35 to 0.1, and the accuracy improvement is from 0.24 to 0.35; In Figure 5(b), the loss of the test set is lower than that of the training set, ranging from 0.22 to 0.03, and the accuracy of the test set is higher.

Through the RO method, the results of the accuracy and loss values in the training set are shown in Figure 6(a), and the test set in Figure 6(b).

In Figure 6(a) and 6(a), the loss and accuracy are very similar to the ST method.

In Figures 4-6, the loss decreases rapidly in less than 10 epochs by DNN-FE. In training set, the decrease of loss is very large, and the increase of accuracy is very small; In test set, loss fluctuates and decreases, while accuracy fluctuates and increases, both of which change little.

Next, the DNN-FE model was used for 100 epochs of training, the average results for loss and accuracy are shown in Table 6.

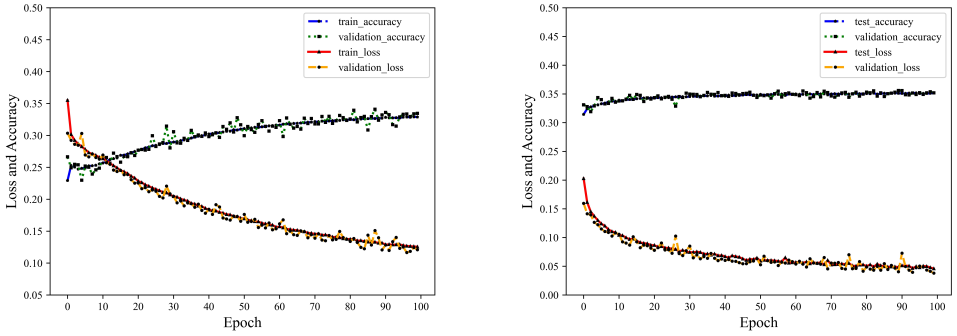

(a) Loss and accuracy of training set by RO (b) Loss and accuracy of test set by ST

Figure 6. Results of training and test set by DNN-FE and RO

Table 6. The mean results of the three methods

			RU	ST	RO
DNN-FE	Training set	Loss	0.072345041	0.062131729	**0.050253212**
		Accuracy	0.311178237	0.350879073	**0.353610069**
	Test set	Loss	0.12824893	0.009888236	**0.00813388**
		Accuracy	0.277666003	**0.359941959**	0.359353542

From Table 6, the best accuracy and lowest loss in training set by RO, and there is very little gap between ST and RO methods. In the test set, the performance is approximated by RO and ST methods. It is worth mentioning that the reduction of the loss span is large from 0.12824893 to 0.072345041 by RU, from 0.062131729 to 0.009888236 by ST and from 0.050253212 to 0.00813388 by RO. The ST and RO methods, which accuracy is approximate in test set, are more accurate than the RU method. Both of loss is similar. Therefore, it is speculated that the feature extraction results of RO and ST methods are similar and better than that of RU method.

Modified Encoder Feature Extractor

The Auto-Encoder is mainly composed of Encoder and Decoder, whose main purpose is to convert input into the intermediate features, then convert the intermediate features into output, and compare input and output to make them infinitely close.

Auto-Encoder(AE) includes encoding (Encoder) and decoding (Decoder) two-phase symmetry structure, and the same number of hidden layers on the encoding and decoding, the structure of the design goal is to get the input layer and output layer, data approximately equal, namely by rebuilding the minimum error to the input For the characteristic representation of information, the encoding process of the Auto-Encoder is shown in Formula 8, where x represents input; w_1 and b_1 represent the weight and bias of the encoding respectively. The decoding process of the Auto-Encoder is shown in Formula 9, where $\hat{x}$ represents the output; w_2 and b_2 represent the weight and bias of decoding respectively. f is a nonlinear activation function acting on changes in the encoding and decoding.

$$y = f(w_1 x + b_1) \tag{8}$$

$$\hat{x} = f(w_2 y + b_2) \tag{9}$$

Since the Encoder of the hidden layer is usually a compressed structure, namely data mining through the encoder, the correlation between characteristics of dimension reduction to obtain a higher level of expression. The structure of Auto-Encoder, which encoding and decoding the process, is shown in Figure 7.

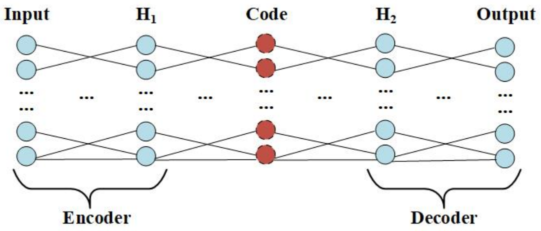

Figure 7. Encoder and Decoder structure

The features learned by the Encoder can be sent into the Ensemble learning model, so the Encoder can play the role of feature extractor named Encoder Feature Extractor (Encoder-FE). In the paper, the output of a hidden layer of the Encoder, as the input of the Ensemble learning model, is the process of training Encoder-FE. The Encoder-FE structure has three steps: The first step is making sure the number of hidden layers. Because increasing the number of layers does not significantly improve the quality, the Encoder-FE structure sets as a single hidden layer. The second step is making sure the number of the hidden layer nodes. The different number of nodes seriously affect the quality of the Encoder-FE. Due to the symmetrical structure of Encoder-FE, the dimensions of the output layer and input layer are the same, which is also 20. The number of Encoder-FE hidden layer nodes are between 0.5 and 6.0 times of features. In other words, the number of hidden layer nodes range from 10 to 120. For the third step, the Encoder-FE adds regularization which is L1. Because L1 regularization can better refine important features and effectively prevent overfitting. The L1 regularization strength is 10^{-5}.

The Encoder-FE results of the training set are shown in Table 7. The accuracy and loss performance of Encoder-FE are ranked by nodes from 10 to 120 through RU, ST, and RO methods. Finally, the results of the best values after training 100 epochs are shown in bold.

Table 7. Accuracy and loss on Encoder-EF under different nodes in training set

No.	RU		ST		RO	
	Accuracy	Loss	Accuracy	Loss	Accuracy	Loss
10	0.205870695	0.50202294	0.246499519	0.317327674	**0.255979386**	**0.269553086**
20	0.164218131	2.037812512	0.252216536	0.376751502	0.245072406	0.414165245
30	0.165242301	2.983963695	0.177956869	1.764019219	0.179491492	1.793878254
40	0.202856797	0.433191515	0.177936916	1.858850295	**0.258656964**	**0.315423219**
50	0.164905745	2.465141864	**0.255602393**	**0.307760783**	0.243757331	0.526701923
60	**0.216074924**	**0.398321054**	0.229109123	0.359815967	0.184790686	1.697652671
70	0.196616918	1.535035479	0.177940629	1.861460186	0.228812747	0.340332582
80	0.177014504	2.057871491	0.234186135	0.587567173	0.219696598	0.494752699
90	**0.210002417**	**0.344811413**	0.191195834	0.35420279	0.204379627	0.377894253
100	0.181033235	1.679312267	0.24950053	0.365956819	0.22031557	0.343383874
110	0.203899698	0.371654426	0.1788566	1.904578166	0.180519055	1.852904
120	0.188723264	1.583452941	0.180390533	1.836395991	0.200355758	0.309168555

In Table 7, For the US way, when the number of Encoder-FE hidden layer nodes is 60, accuracy is best; when the number of nodes is 90, loss is best; When we consider both accuracy and loss, the number of nodes is 60. For the ST and RO ways, the best results are those with a nodes number of 50 and 40; So the nodes number of the hidden layer are determined to be 60,50, and 40. When the Encoder-FE hidden layer nodes number of 60,50, and 40, the accuracy and loss in training and test set with 100 epochs are shown in Figure 8-10.

In Figure 8(a), the loss of the training set decreased very rapidly, but accuracy improved only a little. In Figure 8(b), the loss and accuracy don't change much.

In Figure 9(a), the loss of the training set decreased very rapidly within 10 epochs, but accuracy fluctuates in the range of 0.1-0.3. In Figure 9(b), the loss and accuracy show an obvious downward and upward trend.

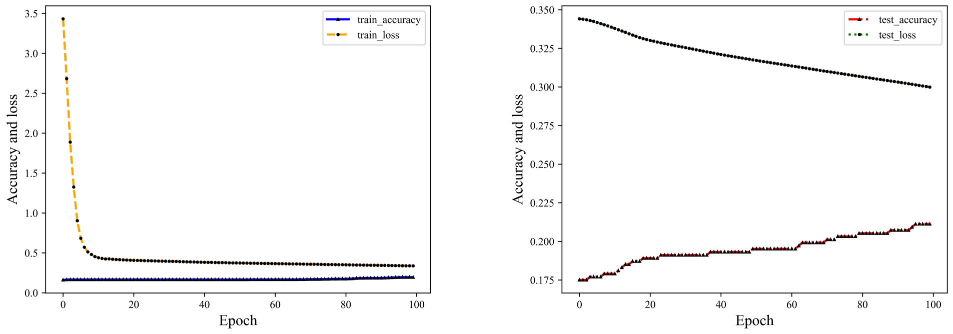

(a) Loss and accuracy of training set by RU

(b) Loss and accuracy of test set by RU

Figure 8. Results of training and test set by Encoder-EF and RU (node = 60)

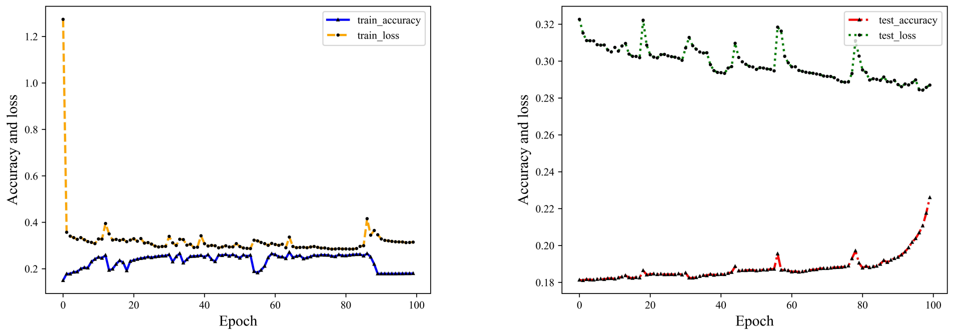

(a) Loss and accuracy of training set by ST

(b) Loss and accuracy of test set by ST

Figure 9. Results of training and test set by Encoder-EF and ST (node = 50)

In Figure 10(a), the loss of the training set also decreased very rapidly within 10 epochs, the accuracy ranges from 0.1 to 0.3. In Figure 10(b), the accuracy improves significantly.

Due to the small number of under-sampling samples, the performance of Encoder-FE by the RU method is not obvious, but the others that ST and RO methods have great performance.

PCA Feature Extractor

Principal component analysis (PCA) is one of the most classic dimension reduction methods, its core idea is through coordinate transformation to map data from high dimension space to low dimension space, making the transformed data maximum variance of the space, the transformed data is called main components, is a linear combination of the original data, at the same time, the conversion process should contain the original data information as possible.

In this paper, the representative PCA was selected as a feature extractor, named PCA Feature Extractor (PCA-FE). In practice, the features cumulative contribution rate (CCR), which this value indicates the amount of information contained in principal components after dimensionality reduction, is usually selected as 95%. The features contribution rate (CR) and CCR of principal components were obtained after the PCA dimension reduction of the original data in the training set, as shown in Table 8.

In the Table 8, the features CCR increases with increasing the number of cumulative features, when it is 10, is greater than 95%. Therefore, the number of cumulative features is 10, which equals the dimension. Both methods, which are ST and RO, are equal which the features CR and CCR. It indirectly indicates that the effects of the two sampling methods are similar.

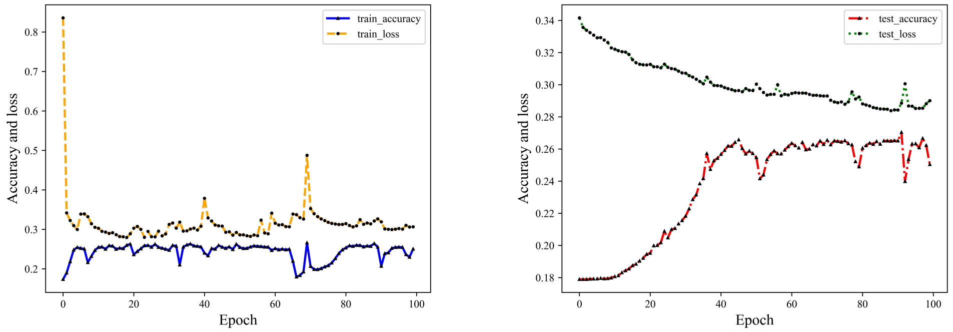

(a) Loss and accuracy of training set by RO

(b) Loss and accuracy of test set by RO

Figure 10. Results of training and test set by Encoder-EF and RO (node = 40)

For three sampling ways, the features CR and CCR are shown in the Figure 11 and 12.

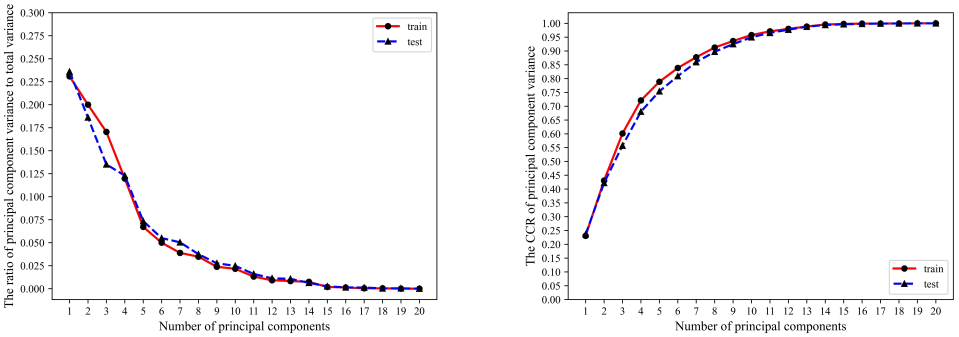

(a) The ratio of principal component variance to total variance

(b) The CCR of principal component variance

Figure 11. The features CR and CCR by RU

After PCA dimension reduction, the line chart, which describes the cumulative contribution rate of training and test set by the three methods, is shown in the Figure 13.

To sum up, this paper constructs three different types of feature extractors which including DNN-FE, Encoder-FE, and PCA-FE. For the DNN-FE, the results of ST and RO are similar and satisfying; For the Encoder-FE, the RO method with 40 nodes works best; And for the PCA-FE, the results of ST and RO are satisfactory.

Ensemble learning

Base learner

Ensemble learning is not only a single machine learning algorithm, but also builds and combines multiple machine learners (Base learners) to complete the learning task. The first part of the Ensemble learning model structure consists four base learners, such as Random Forest, XGBoost, LightGBM, and GDBT. For details, the accuracy and loss are shown in Table 9, and the best values are in bold. The accuracy and loss are the mean values of 3 cycles of 5-fold cross validation in training and test set.

In Table 9, LightGBM and GDBT perform well in both training and test set. In ST and RO ways, LightGBM performs better than GDBT in the test set, but GDBT in the training set. Therefore, the LightGBM or GDBT are the second part of Ensemble learning model. The base learners train with 5-fold

Table 8. The CR and the CCR of PCA

No.	RU		ST		RO	
x	y1	y2	y1	y2	y1	y2
1	0.2308	0.2308	0.2788	0.2788	0.2788	0.2788
2	0.2000	0.4308	0.2424	0.5212	0.2424	0.5212
3	0.1704	0.6013	0.1647	0.6859	0.1647	0.6859
4	0.1197	0.7210	0.0907	0.7767	0.0907	0.7767
5	0.0672	0.7883	0.0715	0.8482	0.0715	0.8482
6	0.0500	0.8383	0.0364	0.8846	0.0364	0.8846
7	0.0389	0.8773	0.0315	0.9162	0.0315	0.9162
8	0.0348	0.9122	0.0198	0.9360	0.0198	0.9360
9	0.0238	0.9360	0.0188	0.9549	0.0188	0.9549
10	0.0216	0.9576	0.0171	0.9720	0.0171	0.9720
11	0.0132	0.9709	0.0083	0.9803	0.0083	0.9803
12	0.0091	0.9800	0.0063	0.9867	0.0063	0.9867
13	0.0083	0.9883	0.0056	0.9923	0.0056	0.9923
14	0.0074	0.9957	0.0042	0.9966	0.0042	0.9966
15	0.0019	0.9977	0.0017	0.9984	0.0017	0.9984
16	0.0013	0.9990	0.0010	0.9994	0.0010	0.9994
17	0.0004	0.9991	0.0003	0.9997	0.0003	0.9997
18	0.0004	0.9995	0.0001	0.9998	0.0001	0.9998
19	0.0	1.0	0.0001	1.0	0.0001	1.0
20	0.0	1.0	0.0	1.0	0.0	1.0

Table 9. The accuracy and loss of four base learners

		RU		ST		RO	
		Training set	Test set	Training set	Test set	Training set	Test set
Random Forest	Accuracy	0.80020141	0.820909091	0.884209836	0.925312914	0.883944179	0.926738174
	Loss	0.686766957	0.737885838	0.41897397	0.388393875	0.424137006	0.38496877
XGBoost	Accuracy	0.818328298	0.81830303	0.899306417	0.931902906	0.899306417	0.931902906
	Loss	0.882461397	0.915079895	0.7529918	0.698919274	0.7529918	0.698919274
LightGBM	Accuracy	**0.822557905**	**0.869845118**	0.905014572	**0.944481528**	0.905014572	**0.944481528**
	Loss	**0.541475823**	**0.473699042**	0.338876355	**0.267208249**	0.338876355	**0.267208249**
GDBT	Accuracy	0.779657603	0.788787879	**0.905400113**	0.917441613	**0.905273771**	0.916460918
	Loss	0.684157963	0.675074413	**0.319928411**	0.315431028	**0.319976762**	0.315293562

cross validation and repeat for 3 cycles. The training results including the accuracy and loss are shown in Figure 14-17.

In Figure 14(a), the accuracy of the test set is better than the training set by ST and RO methods. But, the accuracy by RU way fluctuate greatly, the effect of the test set is not necessarily higher than the training set. In Figure 14(b), the loss of the test set is lower than the training set and ranging from 0.1 to 0.3 by ST and RO methods. The very poor effect of the loss value by RU way may have an important relationship with the number of samples.

In Figure 15, the accuracy and loss results of ST and RO methods are the same and the effect is good, while RU method has a poor effect.

In Figure 16, the accuracy and loss results is the best among the four base learners. The both have the same effect by ST and RO methods.

In Figure 17, the results of GDBT is only lower than LightGBM. The GDBT have the different effect by ST and RO methods.

According to Table 9 and the historical training results in Figure 14-17, it is finally concluded that the performance of LightGBM is great, which as the second layer of Ensemble learning model for the next training, and GDBT also.

Stacking

Stacking can be regarded as learning a model to combine several existing models. The algorithm that Stacking is a two-layer structure: the first layer is called base classifier, and the second layer is called

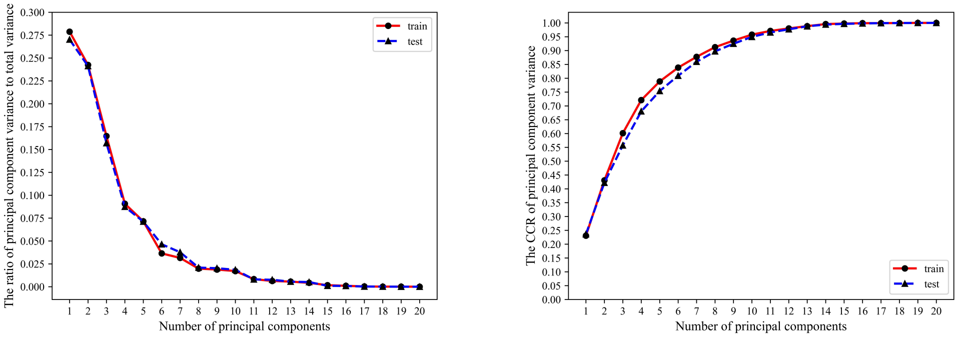

(a) The ratio of principal component variance to total variance

(b) The CCR of principal component variance

Figure 12. The features CR and CCR by ST and RO

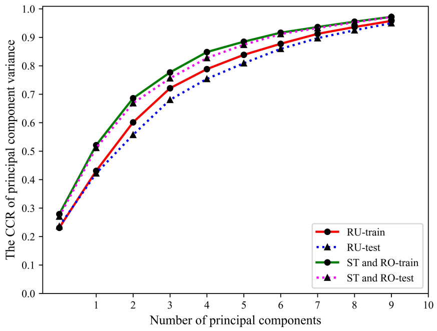

Figure 13. The features CCR by RU, ST, and RO

meta classifier. The four base models that Random Forest, XGBoost, LightGBM, and GDBT are 5-fold cross validation as the base classifier. The meta classifier is LightGBM or GDBT with better effect. The both parts are Stacking. For details, the accuracy and loss by Stacking are shown in Table 10, and the best values are in bold.

Table 10. The accuracy and loss of four base learners

		RU		ST		RO	
		Training set	Test set	Training set	Test set	Training set	Test set
Stacking	Accuracy	**0.842497482**	0.741792548	0.857246506	0.881522825	0.857246506	0.881522825
(LightGBM)	Loss	**0.492987796**	0.751033209	0.44708934	0.388245438	0.44708934	0.388245438
Stacking	Accuracy	0.821148036	**0.767371601**	**0.949484746**	**0.923953099**	**0.94918022**	**0.924070682**
(GDBT)	Loss	0.563468623	**0.743530804**	**0.21509402**	**0.269465728**	**0.214794269**	**0.268281244**

According to the results in Table 10, the best performing model is the two-layer structure of Stacking: the base classifier (Random Forest, XGBoost, LightGBM, and GDBT) and the meta classifier (GDBT). The training results, which including the value of accuracy and loss by RU, ST and RO, are shown in Figure 18-20.

In Figure 18, the gap between the maximum value and the minimum value of GDBT by RU method is larger than that of LightGBM, because the final value is the average value. So, the value of LightGBM by RU method is better.

In Figure 19, the accuracy of GDBT higher than LightGBM, and loss of GDBT is lower. So, the value of GDBT by ST method is better.

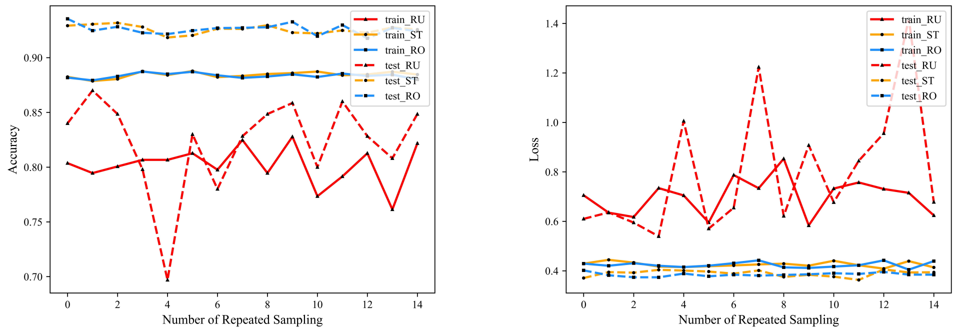

(a) The accuracy of training and test set

(b) The loss of training and test set

Figure 14. Results of training and test set by Random Forest

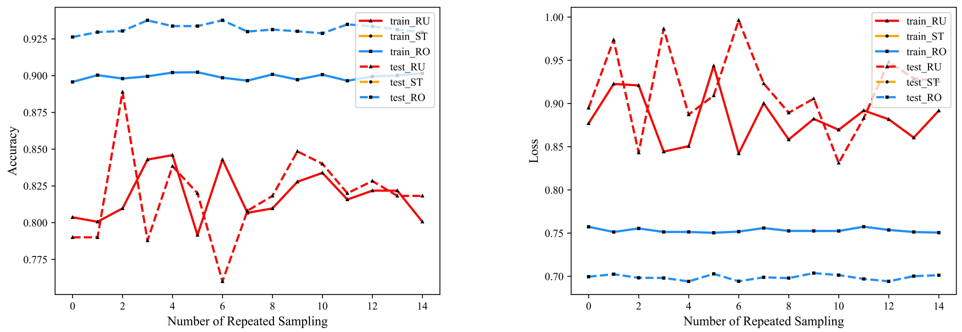

(a) The accuracy of training and test set

(b) The loss of training and test set

Figure 15. Results of training and test set by XGBoost

In Figure 20, the accuracy and loss value of GDBT by RO method is better. It is particularly worth mentioning, which the LightGBM test set is better than the training set.

Finally, the second layer (meta classifier), which is the Stacking model with GDBT, is better. Since LightGBM's result is approximated, and is also added to the hybrid model as a comparison.

Hybrid credit risk model

The feature extractor extracts the deeper data features. The first layer (base classifier) of Stacking by 5-fold cross validation is used to train the results as new features. The new features as the input of the second layer (meta classifier) to prevent model overfitting. The hybrid model is to concatenate the data extracted from the feature extractors (including DNN-FE, Encoder-FE, and PCA-FE) with the new features extracted from the base classifier, which is used as the input of the meta classifier.

This hybrid models can not only excavate the deeper features of the data, but also add new features and enrich the features of the data. The structure of hybrid models named way as the first part is the name of the feature extraction apparatus, in the second part is the name of the second layer of Stacking, such as the feature extraction with DNN-FE and the second layer of Stacking with LightGBM, the hybrid model name for DNN-FE+LightGBM. The hybrid models result of the three sampling methods are compared as shown in the Table 11.

From the Table 11, for the results of training set by RU, the Encoder-FE+LightGBM is the best accuracy, the PCA-FE+LightGBM is the least loss, and Encoder-FE+LightGBM or GDBT is the best accuracy and loss; For the results of training and test set by ST and RO ways, PCA-FE+LightGBM and

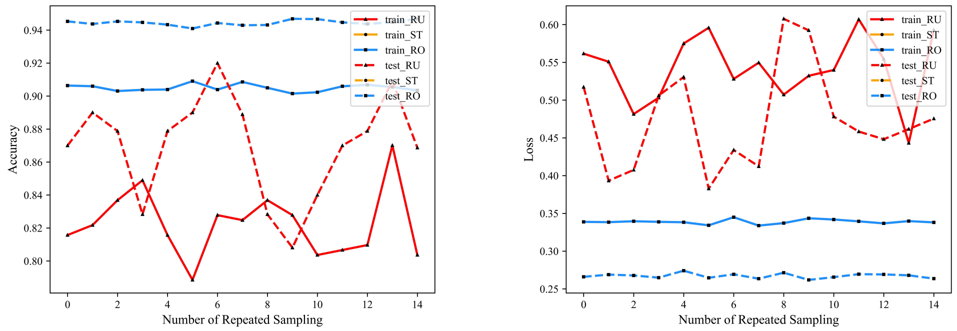

(a) The accuracy of training and test set

(b) The loss of training and test set

Figure 16. Results of training and test set by LightGBM

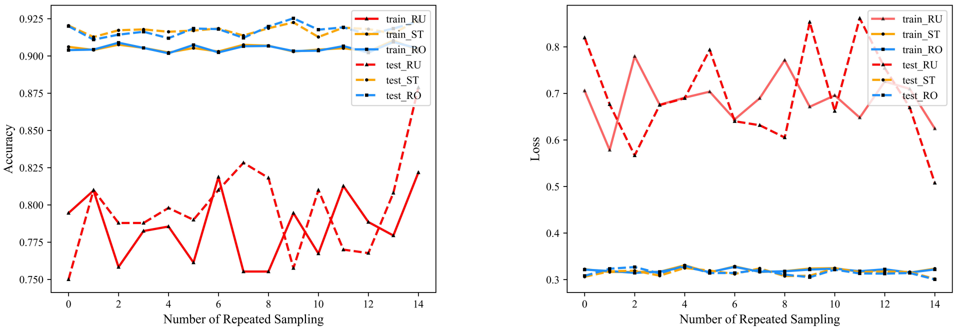

(a) The accuracy of training and test set

(b) The loss of training and test set

Figure 17. Results of training and test set by GDBT

Encoder-FE+GDBT are the best accuracy and loss. Moreover, the effect of Encoder-FE+GDBT is better than PCA-FE+LightGBM. The performance of hybrid credit risk models with three feature extractions is very close and great. Among the three sampling methods, the performance of RO and ST method is obviously better than that of RU, which is very unfriendly to the small sample size.

Finally, a comparison result in Ensemble learning and hybrid credit risk model, which GDBT, Stacking (GDBT) and Encoder-FE+GDBT, is shown in Table 12.

According to the results in Table 12, there is a small difference between the results of Stacking (GDBT) and hybrid credit risk model (Encoder-FE+GDBT). The results of ST are good, but the effect of the test set by RO is better. Both models, which Stacking (GDBT) and Encoder-FE+GDBT, are a significant improvement over the GDBT effect in training and test sets. For example, accuracy rate goes from 0.779657603 to 0.949484746, loss plummets from 0.684157963 to 0.213328147. By GDBT, Stacking (GDBT) and Encoder-FE+GDBT, the results of the comparison are shown in Figure 21 and 22.

In Figure 21 and 22, the accuracy of Stacking (GDBT) and Encoder-FE+GDBT is slightly improved, but the loss is greatly reduced rather than GDBT.

Analysis

The three hybrid models, which DNN-FE+GDBT, PCA-FE+GDBT, and Encoder-FE+GDBT propose in this paper. The experimental results between three hybrid models and the base model GDBT are shown in the Table 13.

For the RU method, after DNN-FE, Encoder-FE and PCA-FE feature extractors adding, the accuracy

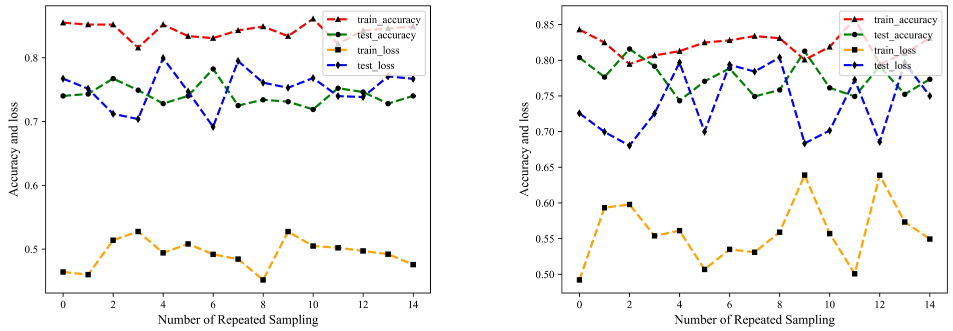

(a) The accuracy and loss of Stacking(LightGBM) (b) The accuracy and loss of Stacking(GDBT)

Figure 18. Results of Stacking(LightGBM) or (GDBT) by RU

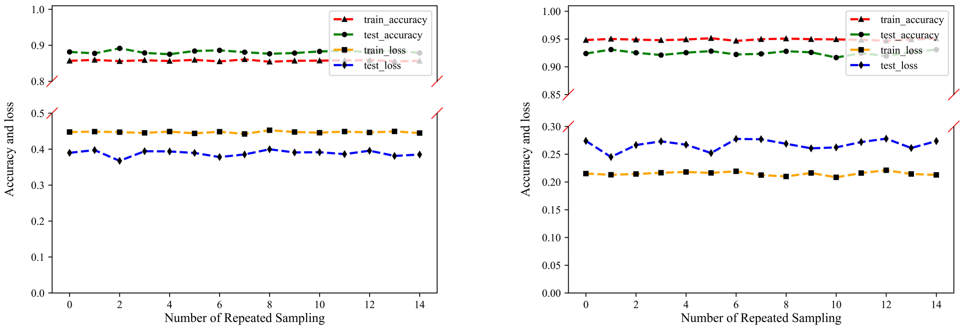

(a) The accuracy and loss of Stacking(LightGBM) (b) The accuracy and loss of Stacking(GDBT)

Figure 19. Results of Stacking(LightGBM) or (GDBT) by ST

and loss of hybrid credit risk models obviously become the worse performance, which sample size is too small to cause this bad situation. Especially, the accuracy decreases significantly after PCA dimension reduction. The reason may be that the characteristics of compression are not representative, and PCA would be meaningless to continue using PCA.

For ST and RO methods, no matter in the training or test set, the accuracy improvement effect of the three hybrid credit risk models which DNN-FE+GDBT, PCA-FE+GDBT, and Encoder-FE+GDBT is similar, and Encoder-FE+GDBT has the best effect. Loss is the same result.

The comparison results of the above models are shown in Figure 23 and 24 .

In the paper, the PCA-FE not improve the model effect, but make the effect worse. PCA has a general performance, which is not applicable to the models in this paper, and is more suitable for compression of high-dimensional original features. In the case of this paper, the performance of DNN-FE is poor, indicating that DNN do not necessarily have advantages as feature extractors. As the point of view of the feature extractor, Encoder-FE performance is the best, indicating that a deep neural network Encoder with a special structure can seek better features, which further reflects the powerful learning ability of the deep model.

RESULTS AND DISCUSSION

For the feature extractors, DNN-FE is 20 nodes of $H4$ layer with dimensions such as the number of features, the Encoder-FE structure is a hidden layer with nodes of 60,50,40 (by RU ST and RO), and PCA

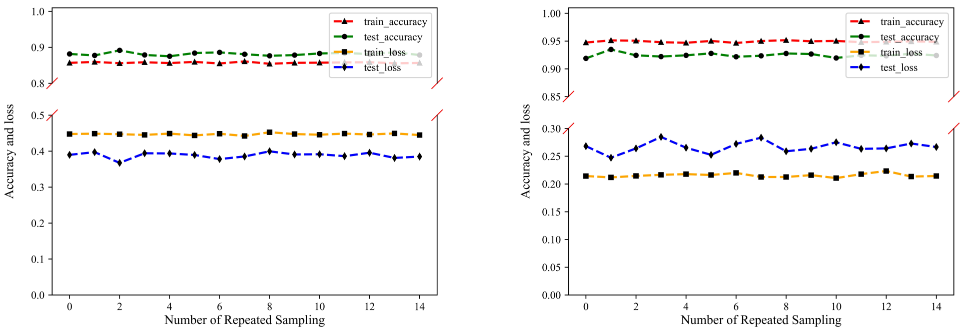

(a) The accuracy and loss of Stacking(LightGBM) (b) The accuracy and loss of Stacking(GDBT)

Figure 20. Results of Stacking(LightGBM) or (GDBT) by RO

Table 11. The accuracy and loss of four base learners

		RU		ST		RO	
		Training set	Test set	Training set	Test set	Training set	Test set
DNN-FE+	Accuracy	0.703927492	0.818731118	0.858769154	0.884504217	0.857405242	0.883209548
LightGBM	Loss	0.78982887	0.526418311	0.44336777	0.380706898	0.445401388	0.381879365
Encoder-	Accuracy	**0.761732125**	**0.836253776**	0.858101789	0.883719657	0.85713963	0.881327049
FE+LightGBM	Loss	0.737395976	**0.497954241**	0.445993667	0.384024339	0.447656644	0.386526802
PCA-FE+	Accuracy	0.744209466	0.824572004	**0.858772373**	**0.885040264**	**0.859034797**	**0.886138711**
LightGBM	Loss	**0.732185166**	0.5135886	**0.441834362**	**0.375201165**	**0.442513064**	**0.376077677**
DNN-FE+	Accuracy	0.60060423	0.658006042	0.948056072	0.919598794	0.947437305	0.923599932
GDBT	Loss	1.087597693	0.999606748	0.22242533	0.285884853	0.222999778	0.280770423
Encoder-	Accuracy	**0.626384693**	**0.679355488**	**0.949300066**	**0.923979068**	**0.948551719**	**0.925848872**
FE+GDBT	Loss	**1.03864035**	**0.926550281**	**0.213328147**	**0.264849724**	**0.215698703**	**0.255333715**
PCA-FE+	Accuracy	0.595568983	0.651560926	0.943860766	0.914643263	0.943588639	0.915833224
GDBT	Loss	1.099122227	1.016438266	0.233357467	0.292038384	0.235875592	0.304823102

reduces dimensionality to dimension equal to 10.

For Ensemble learning, four base learners firstly such as Random Forest, XGBoost, LightGBM and GDBT are training. Their performance selected as the second layer that LightGBM and GDBT is better. The first layer of Stacking is Random Forest, XGBoost, LightGBM and GDBT are used for 5-fold cross-validation, the output result is the mean of the 5-fold cross-validation, which is the new features and serves as the input of the second layer. The first layer and the second layer are stacked to get a better Ensemble learning model for this study.

Finally, the feature extractors and the Ensemble learning models are combined, which named hybrid credit risk models. The experimental results are summarized as follows:

1. The accuracy of feature extractions is poor, which ranges from 0.2 to 0.36. The reason is that the data is compressed due to dimensionality reduction.

2. For the base learners, the best result of training set is GDBT that accuracy and loss by ST are 90.54% and 0.3199; In test set, the best is LightGBM that accuracy and loss by ST and RO are 94.45% and 0.2672. For Stacking (GDBT), the best results are 94.95% and 0.2151 in training set by ST, and 92.41% and 0.2683 in test set by RO. After Stacking, the accuracy of GDBT increases from 90.54% to 94.95% in training set by ST, and the loss reduces from 0.3199 to 0.2151.

3. After Stacking, there is a significant leap in accuracy, and the loss value drops obviously; However, there is a special result that needs to be explained: the loss value of the test set by RU is not decreased, but increased, and the effect is not as good as the results of the basic model (GDBT).

4. For the hybrid credit risk models, the best results of training set by ST are 94.95% and 0.2151, and are 92.58% and 0.2553 by RO and test set. The hybrid credit risk model that works best is named

Table 12. The accuracy and loss of four base learners

		ST		RO	
		Training set	Test set	Training set	Test set
GDBT	Accuracy	0.779657603	0.788787879	0.905400113	0.917441613
	Loss	0.684157963	0.675074413	0.319928411	0.315431028
Stacking(GDBT)	Accuracy	**0.949484746**	0.923953099	**0.94918022**	0.924070682
	Loss	0.21509402	0.269465728	**0.214794269**	0.268281244
Encoder-FE+GDBT	Accuracy	0.949300066	**0.923979068**	0.948551719	**0.925848872**
	Loss	**0.213328147**	**0.264849724**	0.215698703	**0.255333715**

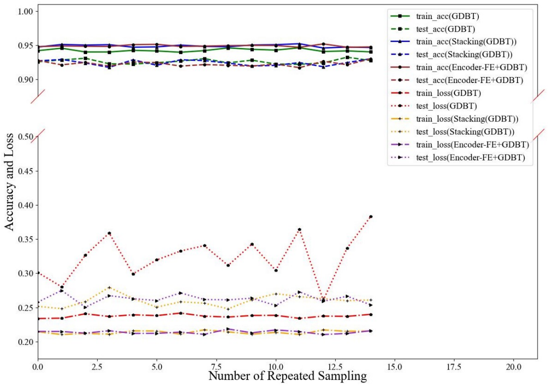

Figure 21. The accuracy and loss by ST

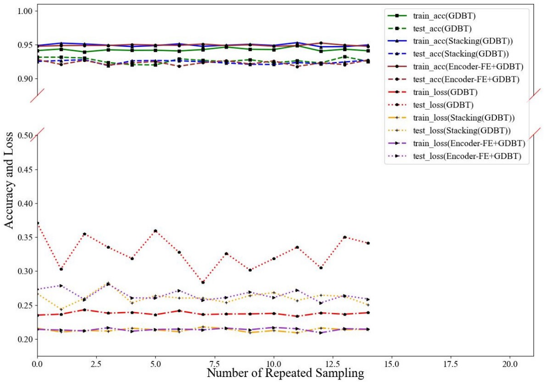

Figure 22. The accuracy and loss by RO

Encoder-FE+GDBT. After models combining, the loss reduces from 0.2151 to 0.2133 and from 0.2695 to 0.2648 in training set and test set by ST; the accuracy increases from 92.41% to 92.58%, and the loss reduces from 0.2683 to 0.2553 in test set by RO.

Although the improvement effect of the hybrid credit risk model is small, it shows that the improvement of the training set and the sampling method are effective and meaningful. And the optimal model of this study is obtained, which is Encoder-FE+GDBT. It is of positive significance to evaluate the risk level from the personal credit data with certain characteristics in the future.

CONCLUSIONS AND FUTURE WORK

The main work of this study is as follows:

1. Since the data in this paper belong to imbalanced data, three sampling methods (RU, ST and RO) are selected to process data at the beginning of the whole paper, which is convenient for subsequent research. Among, the performance effect of ST and RO is close and good, while the effect of RU is not satisfactory, which is caused by too few under-sampling samples.

2. For the accuracy of feature extractions, it is not good and ranges from 0.2 to 0.36, but the focus of feature extractions is not accuracy. This paper is more interested in mining the deep feature information of the data, for example, the DNN selected has this effect.

3. In ensemble learning, four basic models are selected for cross validation in the first layer, and the average value is taken as the new feature in the second layer. In the second layer, the GDBT of Stacking is the best.

4. The feature extraction and ensemble learning are combined, and the original data and the feature extraction data are input into the ensemble learning optimal model training, the Encoder-FE+GDBT model has the best effect.

Table 13. The accuracy and loss of four base learners

		RU		ST		RO	
		Training set	Test set	Training set	Test set	Training set	Test set
GDBT	Accuracy	0.779657603	0.788787879	0.905400113	0.917441613	0.905273771	0.916460918
	Loss	0.684157963	0.675074413	0.319928411	0.315431028	0.319976762	0.315293562
DNN-FE+GDBT	Accuracy	0.60060423	0.658006042	0.948056072	0.919598794	0.947437305	0.923599932
	Loss	1.087597693	0.999606748	0.22242533	0.285884853	0.222999778	0.280770423
PCA-FE+GDBT	Accuracy	0.595568983	0.651560926	0.943860766	0.914643263	0.943588639	0.915833224
	Loss	1.099122227	1.016438266	0.233357467	0.292038384	0.235875592	0.304823102
Encoder-FE+GDBT	Accuracy	0.626384693	0.679355488	0.949300066	0.923979068	0.948551719	0.925848872
	Loss	1.03864035	0.926550281	0.213328147	0.264849724	0.215698703	0.255333715

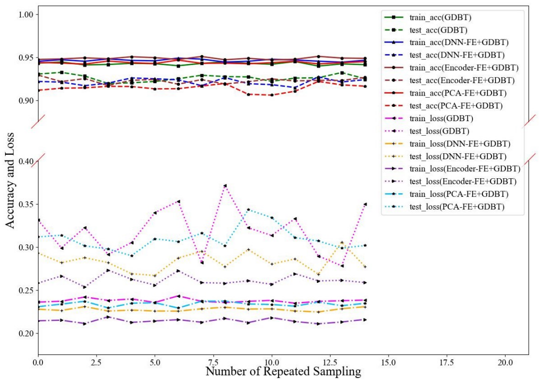

Figure 23. The accuracy and loss by ST

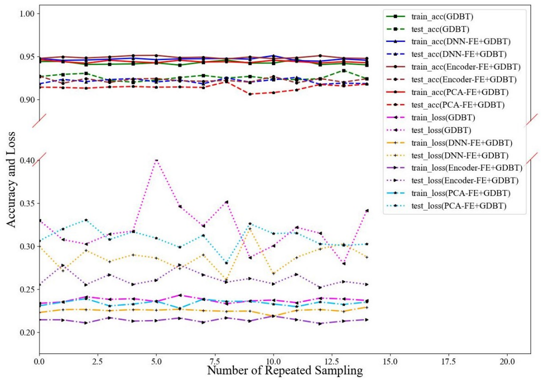

Figure 24. The accuracy and loss by RO

The purpose of the current study is to determine the initial grade of personal credit risk by mining the deep features of existing personal credit data. The ST and RO sampling methods solve the problem of sample data imbalance well and make the sample size sufficient. Over-sampling may lead to data overfitting, but ST method is a new sampling method and has similar effects to RO, so ST sampling method can be selected to carry out more experiments. In the results of the experiment, we learn that the features of PCA compression do not improve the accuracy and cause more losses, DNN-FE that the deep neural network with a common structure has no advantage as a feature extractor. Encoder-FE performance is great, which a deep neural network Encoder with a special structure? Encoder-FE is a new and feasible method to mine features.

The hybrid credit risk model of this study still has some shortcomings, for example, the results of the model are not well interpretable. For the appearance of special results, the loss value of the test set by RU is not decreased, but increased, not explaines well from the model itself. The range of change is not large after Stacking, But the results of basic learners with few samples have a wide range of fluctuations, taking the mean value is not the best decision. The future research work of this study includes:

1. Improve the effect of the Encoder-FE+GDBT model by ST to better assess the personal credit risk level of new users.

2. When the number of samples is small, it is obviously inappropriate to further select the evaluation index of the model and choose the mean value with a large range.

3. The optimal model (Encoder-FE+GDBT) is applied to more classification problems in finance to realize the application of diverse data and experiment the generalization ability of the model.

This research helps us to dig deep personal credit information characteristics to better help us evaluate the personal credit risk level of new customers. It provides new ideas and methods for banks and other financial institutions to assess the credit risk of new users.

ACKNOWLEDGMENTS

This work was supported by the Innovation Fund of Industry, Education and Research of China University (2021LDA11003) and the 2023 Graduate Teaching Reform Project of Capital University of Economics and Business

COMPETING INTERESTS

The authors declare that they have no competing interests.

CONSENT FOR PUBLICATION

All authors consent to the final version for publication.

AUTHOR CONTRIBUTIONS

- Yuanyuan Wang and Zhuang Wu processed the data and designed the experiments, wrote the draft of the paper, and approved the final draft.
- Jing Gao collected data, conducted data mining and analysis, and finally reviewed the manuscript.
- Chenjun Liu and Fangfang Guo assisted in the experimental inspection and result analysis, supplemented the polishing of the paper, and completed the approval of the final manuscript.

DATA AVAILABILITY

The raw data and code are available in the Supplemental Files.

SUPPLEMENTAL INFORMATION

Supplemental information for this article can be found online at doi :

REFERENCES

Abellán, J. and Castellano, J. G. (2017). A comparative study on base classifiers in ensemble methods for credit scoring. *Expert systems with applications*, 73:1–10.
AF Ferreira, F., P. Santos, S., SE Marques, C., and Ferreira, J. (2014). Assessing credit risk of mortgage lending using macbeth: a methodological framework. *Management Decision*, 52(2):182–206.
Arora, N. and Kaur, P. D. (2020). A bolasso based consistent feature selection enabled random forest classification algorithm: An application to credit risk assessment. *Applied Soft Computing*, 86:105936.
Behr, A. and Weinblat, J. (2017). Default patterns in seven eu countries: A random forest approach. *International Journal of the Economics of Business*, 24(2):181–222.
Chawla, N. V., Bowyer, K. W., Hall, L. O., and Kegelmeyer, W. P. (2002). Smote: synthetic minority over-sampling technique. *Journal of artificial intelligence research*, 16:321–357.
Chen, W., Ma, C., and Ma, L. (2009). Mining the customer credit using hybrid support vector machine technique. *Expert systems with applications*, 36(4):7611–7616.
Dahiya, S., Handa, S., and Singh, N. (2017). A feature selection enabled hybrid-bagging algorithm for credit risk evaluation. *Expert Systems*, 34(6):e12217.
De Andres, J., Lorca, P., de Cos Juez, F. J., and Sánchez-Lasheras, F. (2011). Bankruptcy forecasting: A hybrid approach using fuzzy c-means clustering and multivariate adaptive regression splines (mars). *Expert Systems with Applications*, 38(3):1866–1875.
Dong, G., Lai, K. K., and Yen, J. (2010). Credit scorecard based on logistic regression with random coefficients. *Procedia Computer Science*, 1(1):2463–2468.
Finlay, S. (2011). Multiple classifier architectures and their application to credit risk assessment. *European Journal of Operational Research*, 210(2):368–378.
Ghosh, A. (2015). Banking-industry specific and regional economic determinants of non-performing loans: Evidence from us states. *Journal of financial stability*, 20:93–104.

Guzmán-Ponce, A., Sánchez, J. S., Valdovinos, R. M., and Marcial-Romero, J. R. (2021). Dbig-us: A two-stage under-sampling algorithm to face the class imbalance problem. *Expert Systems with Applications*, 168:114301.

Hsieh, N.-C. and Hung, L.-P. (2010). A data driven ensemble classifier for credit scoring analysis. *Expert systems with Applications*, 37(1):534–545.

Kearns, M. J., Schapire, R. E., and Sellie, L. M. (1992). Toward efficient agnostic learning. In *Proceedings of the fifth annual workshop on Computational learning theory*, pages 341–352.

Khashman, A. (2010). Neural networks for credit risk evaluation: Investigation of different neural models and learning schemes. *Expert Systems with Applications*, 37(9):6233–6239.

Kruppa, J., Schwarz, A., Arminger, G., and Ziegler, A. (2013). Consumer credit risk: Individual probability estimates using machine learning. *Expert systems with applications*, 40(13):5125–5131.

Lenka, S. R., Bisoy, S. K., Priyadarshini, R., and Sain, M. (2022). Empirical analysis of ensemble learning for imbalanced credit scoring datasets: A systematic review. *Wireless Communications and Mobile Computing*, 2022.

Lessmann, S. and Voß, S. (2009). A reference model for customer-centric data mining with support vector machines. *European Journal of Operational Research*, 199(2):520–530.

Li, H., Adeli, H., Sun, J., and Han, J.-G. (2011). Hybridizing principles of topsis with case-based reasoning for business failure prediction. *Computers & Operations Research*, 38(2):409–419.

Lin, S. L. (2009). A new two-stage hybrid approach of credit risk in banking industry. *Expert Systems with Applications*, 36(4):8333–8341.

Louzis, D. P., Vouldis, A. T., and Metaxas, V. L. (2012). Macroeconomic and bank-specific determinants of non-performing loans in greece: A comparative study of mortgage, business and consumer loan portfolios. *Journal of Banking & Finance*, 36(4):1012–1027.

Malik, M. and Thomas, L. C. (2010). Modelling credit risk of portfolio of consumer loans. *Journal of the Operational Research Society*, 61(3):411–420.

Mirzaei, B., Nikpour, B., and Nezamabadi-Pour, H. (2020). An under-sampling technique for imbalanced data classification based on dbscan algorithm. In *2020 8th Iranian Joint Congress on Fuzzy and intelligent Systems (CFIS)*, pages 21–26. IEEE.

Oreski, S. and Oreski, G. (2014). Genetic algorithm-based heuristic for feature selection in credit risk assessment. *Expert systems with applications*, 41(4):2052–2064.

Peng, Y., Wang, G., Kou, G., and Shi, Y. (2011). An empirical study of classification algorithm evaluation for financial risk prediction. *Applied Soft Computing*, 11(2):2906–2915.

Pławiak, P., Abdar, M., Pławiak, J., Makarenkov, V., and Acharya, U. R. (2020). Dghnl: A new deep genetic hierarchical network of learners for prediction of credit scoring. *Information Sciences*, 516:401–418.

Psillaki, M., Tsolas, I. E., and Margaritis, D. (2010). Evaluation of credit risk based on firm performance. *European journal of operational research*, 201(3):873–881.

Sun, J., Lang, J., Fujita, H., and Li, H. (2018). Imbalanced enterprise credit evaluation with dte-sbd: Decision tree ensemble based on smote and bagging with differentiated sampling rates. *Information Sciences*, 425:76–91.

Tong, E. N., Mues, C., and Thomas, L. C. (2012). Mixture cure models in credit scoring: If and when borrowers default. *European Journal of Operational Research*, 218(1):132–139.

Tsai, C.-F. and Chen, M.-L. (2010). Credit rating by hybrid machine learning techniques. *Applied soft computing*, 10(2):374–380.

Tsai, C.-F., Hsu, Y.-F., and Yen, D. C. (2014). A comparative study of classifier ensembles for bankruptcy prediction. *Applied Soft Computing*, 24:977–984.

Wang, G., Hao, J., Ma, J., and Jiang, H. (2011). A comparative assessment of ensemble learning for credit scoring. *Expert systems with applications*, 38(1):223–230.

Wang, G. and Ma, J. (2012). A hybrid ensemble approach for enterprise credit risk assessment based on support vector machine. *Expert Systems with Applications*, 39(5):5325–5331.

Xia, Y., Liu, C., Da, B., and Xie, F. (2018). A novel heterogeneous ensemble credit scoring model based on bstacking approach. *Expert Systems with Applications*, 93:182–199.

Xia, Y., Liu, C., Li, Y., and Liu, N. (2017a). A boosted decision tree approach using bayesian hyper-parameter optimization for credit scoring. *Expert systems with applications*, 78:225–241.

Xia, Y., Liu, C., and Liu, N. (2017b). Cost-sensitive boosted tree for loan evaluation in peer-to-peer

lending. *Electronic Commerce Research and Applications*, 24:30–49.

Yeh, I.-C. and Lien, C.-h. (2009). The comparisons of data mining techniques for the predictive accuracy of probability of default of credit card clients. *Expert systems with applications*, 36(2):2473–2480.

Yu, L., Zhou, R., Tang, L., and Chen, R. (2018). A dbn-based resampling svm ensemble learning paradigm for credit classification with imbalanced data. *Applied Soft Computing*, 69:192–202.

Zambaldi, F., Aranha, F., Lopes, H., and Politi, R. (2011). Credit granting to small firms: A brazilian case. *Journal of Business Research*, 64(3):309–315.

Zhou, L., Lai, K. K., and Yu, L. (2010). Least squares support vector machines ensemble models for credit scoring. *Expert systems with applications*, 37(1):127–133.

Zhu, X., Li, J., Wu, D., Wang, H., and Liang, C. (2013). Balancing accuracy, complexity and interpretability in consumer credit decision making: A c-topsis classification approach. *Knowledge-Based Systems*, 52:258–267.
