# Supplementary figures and images for: A multi-level classification based ensemble and feature extractor for credit risk assessment
Source: PeerJ Comput Sci. 2024 Feb 29;10:e1915. doi: 10.7717/peerj-cs.1915 (PMC10909241; doi:10.7717/peerj-cs.1915)

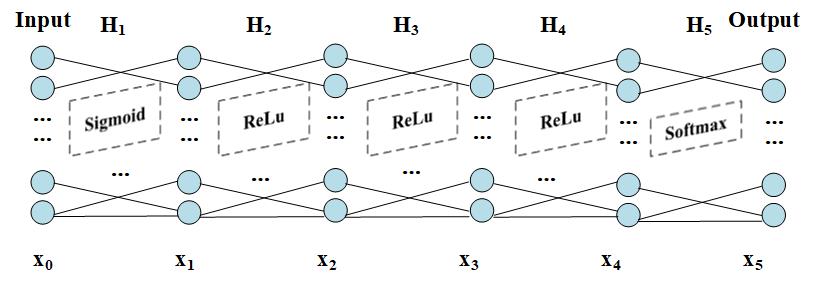

Supplement: Supplemental Information 1 — Model training results and comparison data. [file peerj-cs-10-1915-s001.zip › latex/4.1.jpg]

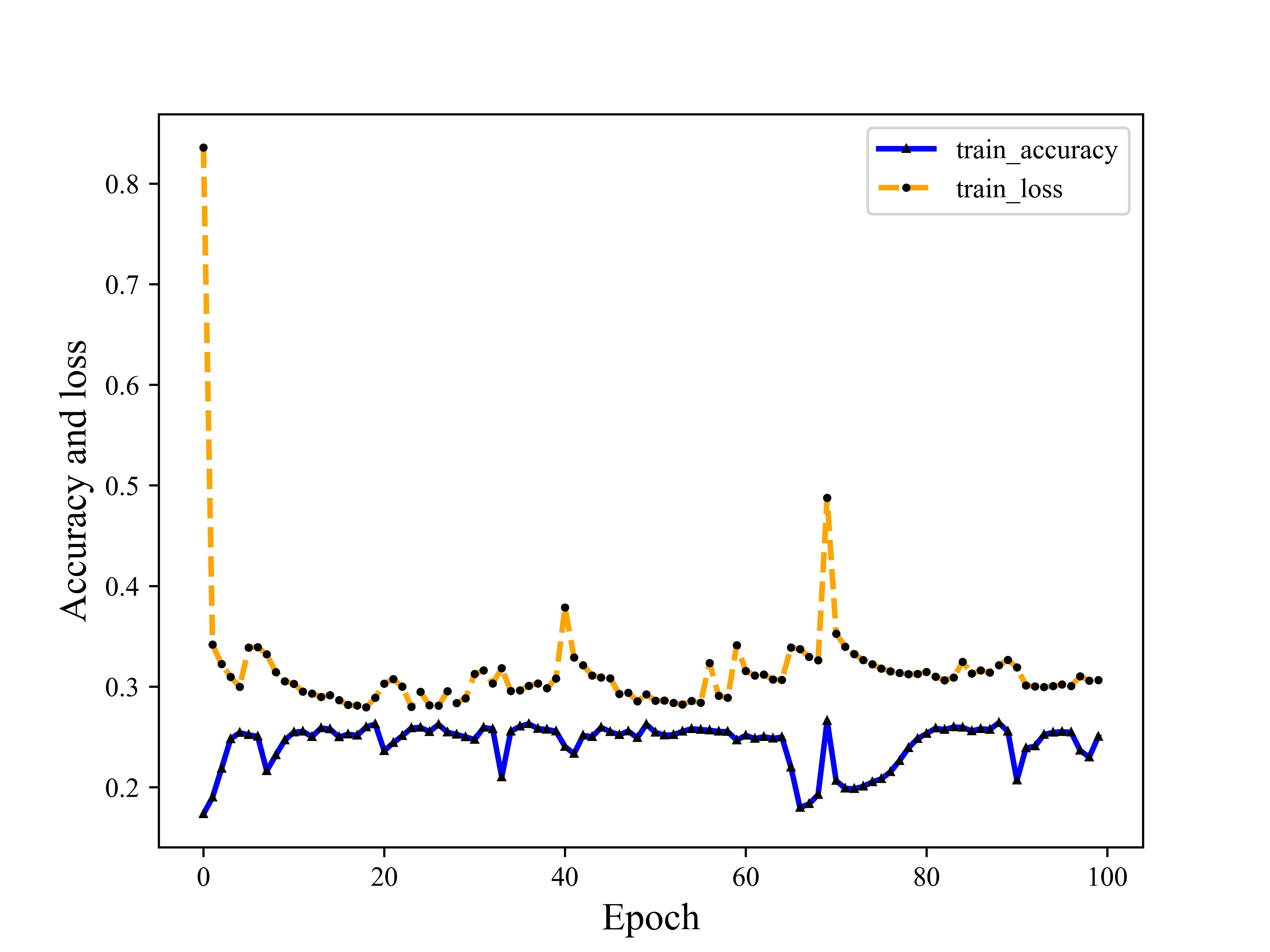

Supplement: Supplemental Information 1 — Model training results and comparison data. [file peerj-cs-10-1915-s001.zip › latex/4.10a.jpg]

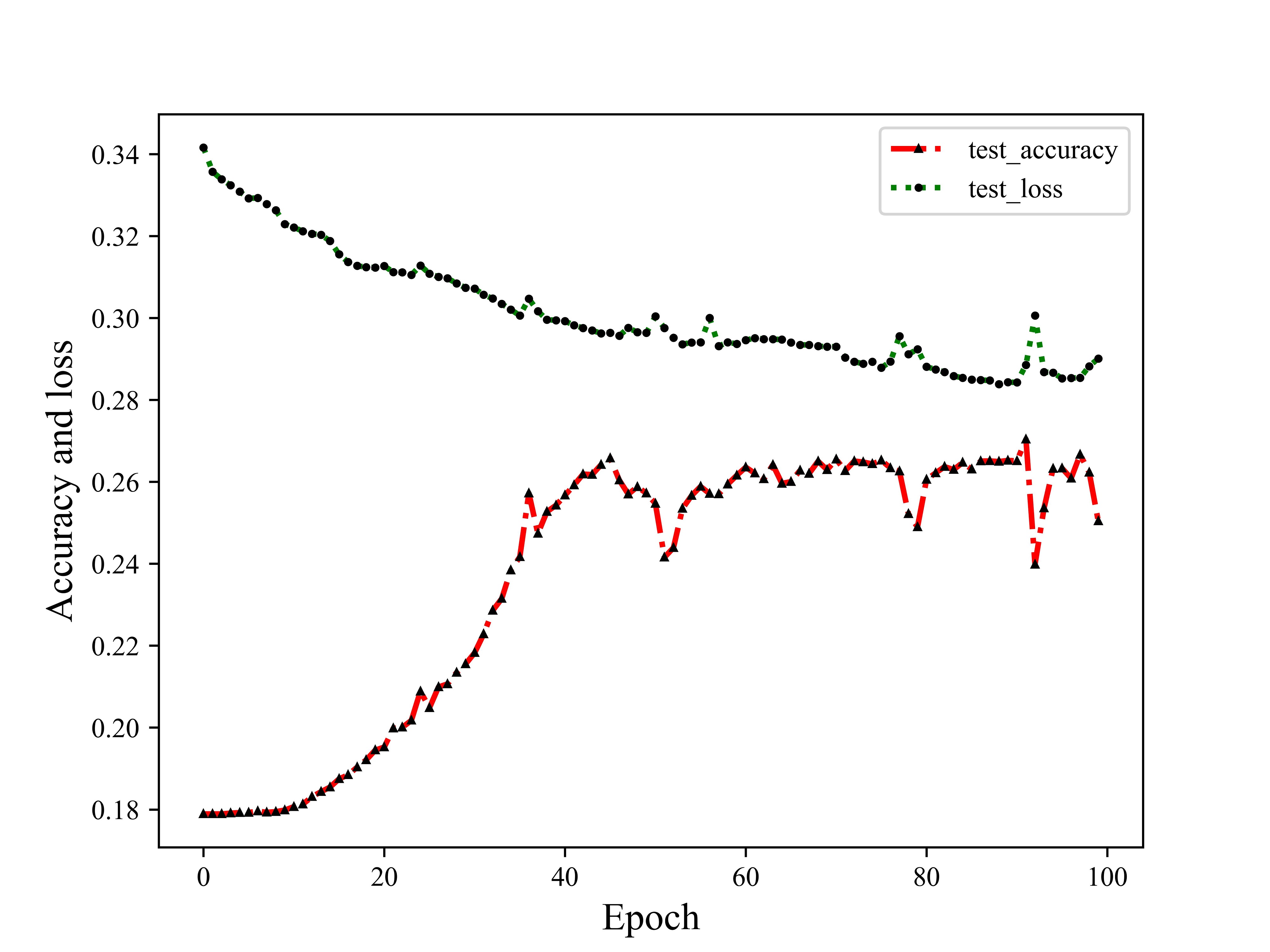

Supplement: Supplemental Information 1 — Model training results and comparison data. [file peerj-cs-10-1915-s001.zip › latex/4.10b.jpg]

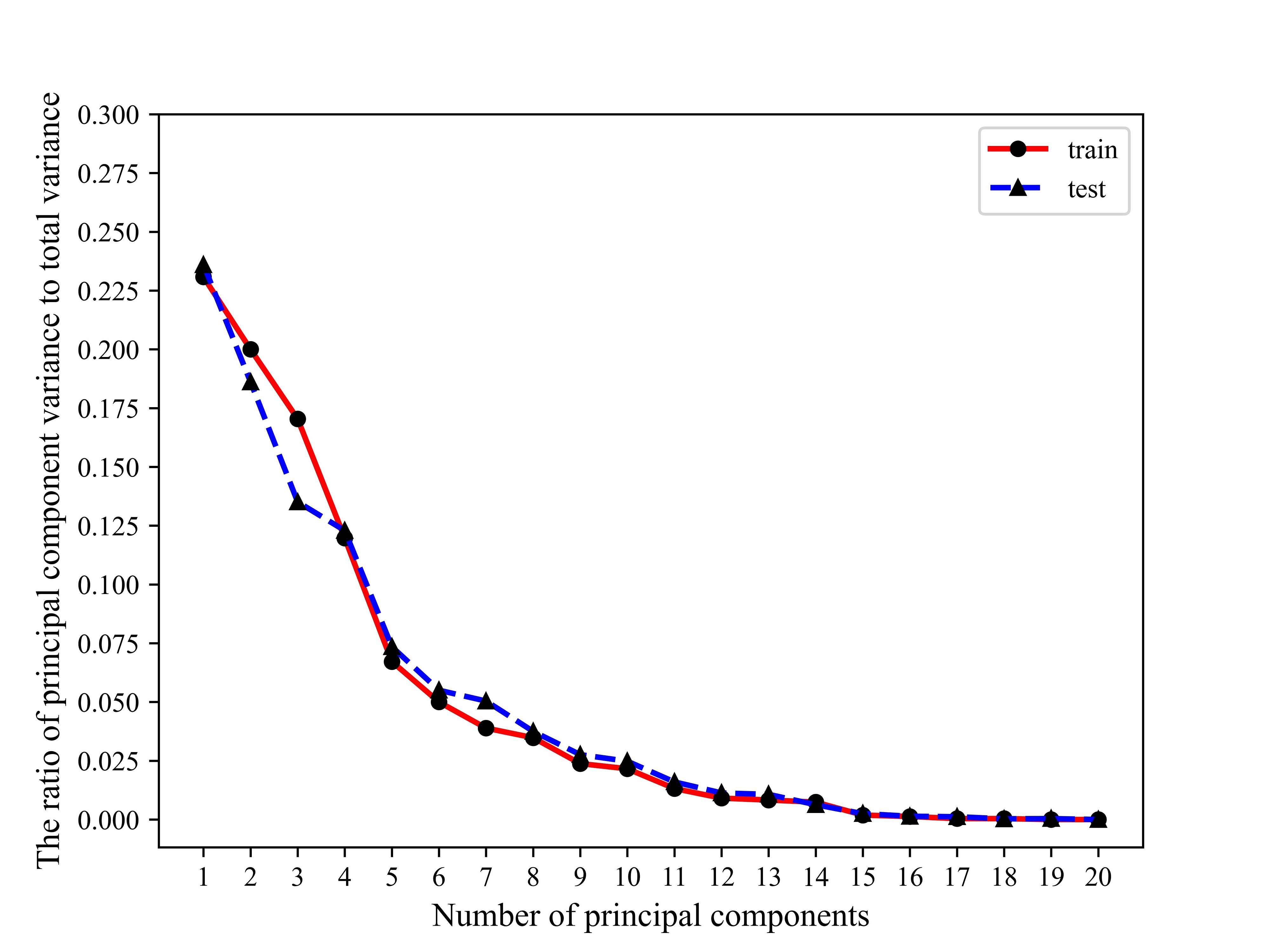

Supplement: Supplemental Information 1 — Model training results and comparison data. [file peerj-cs-10-1915-s001.zip › latex/4.11a.jpg]

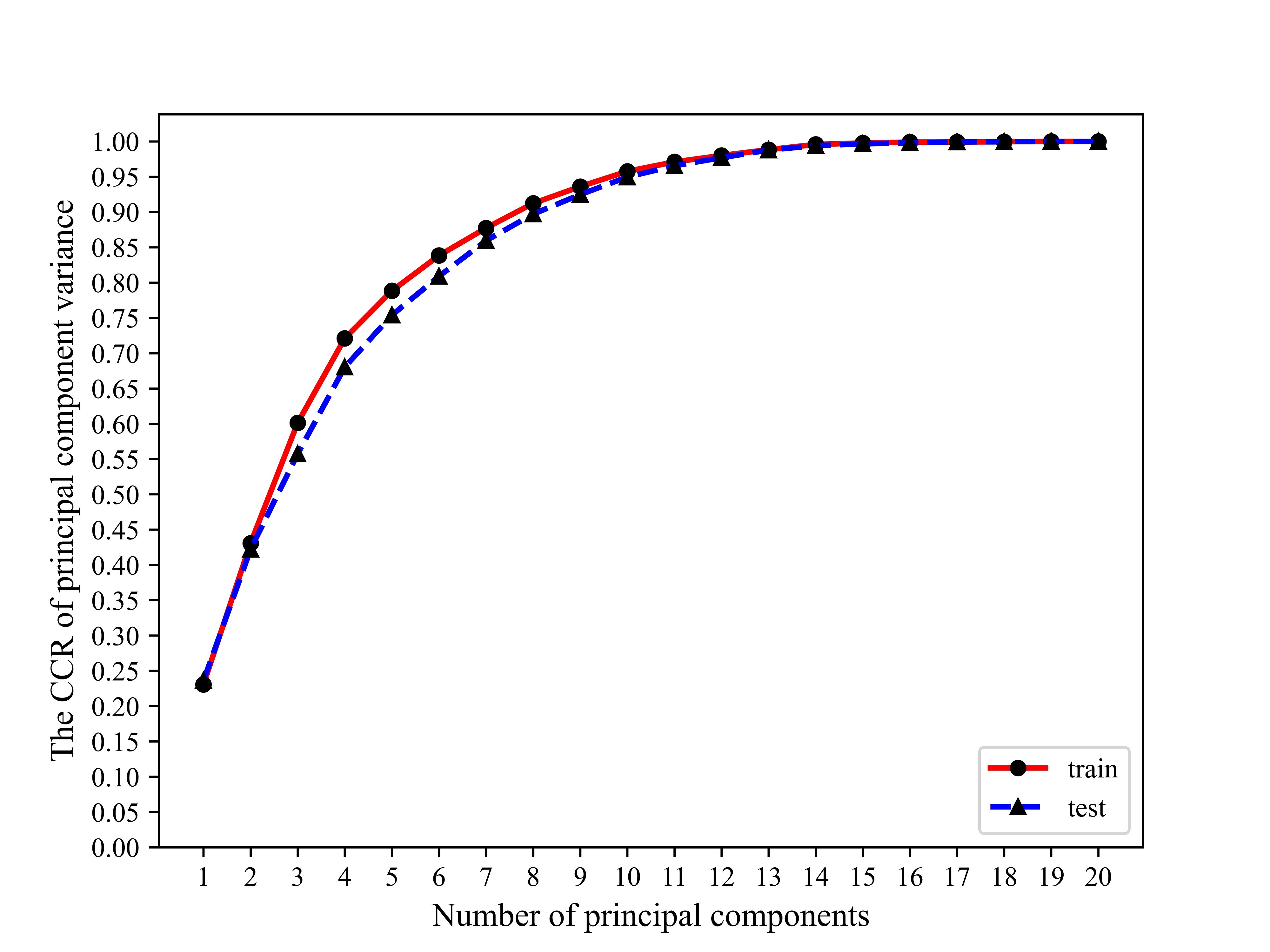

Supplement: Supplemental Information 1 — Model training results and comparison data. [file peerj-cs-10-1915-s001.zip › latex/4.11b.jpg]

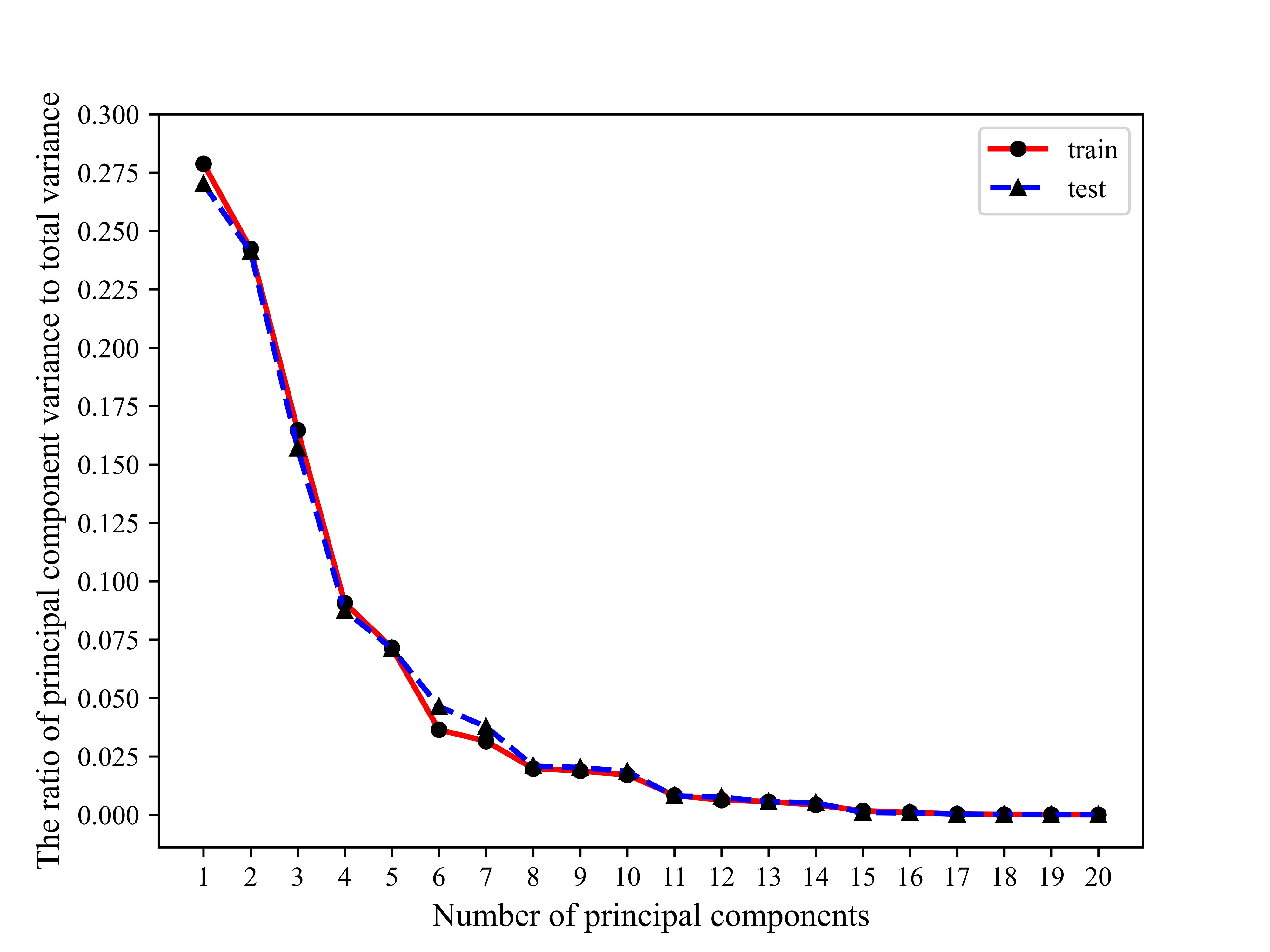

Supplement: Supplemental Information 1 — Model training results and comparison data. [file peerj-cs-10-1915-s001.zip › latex/4.12a.jpg]

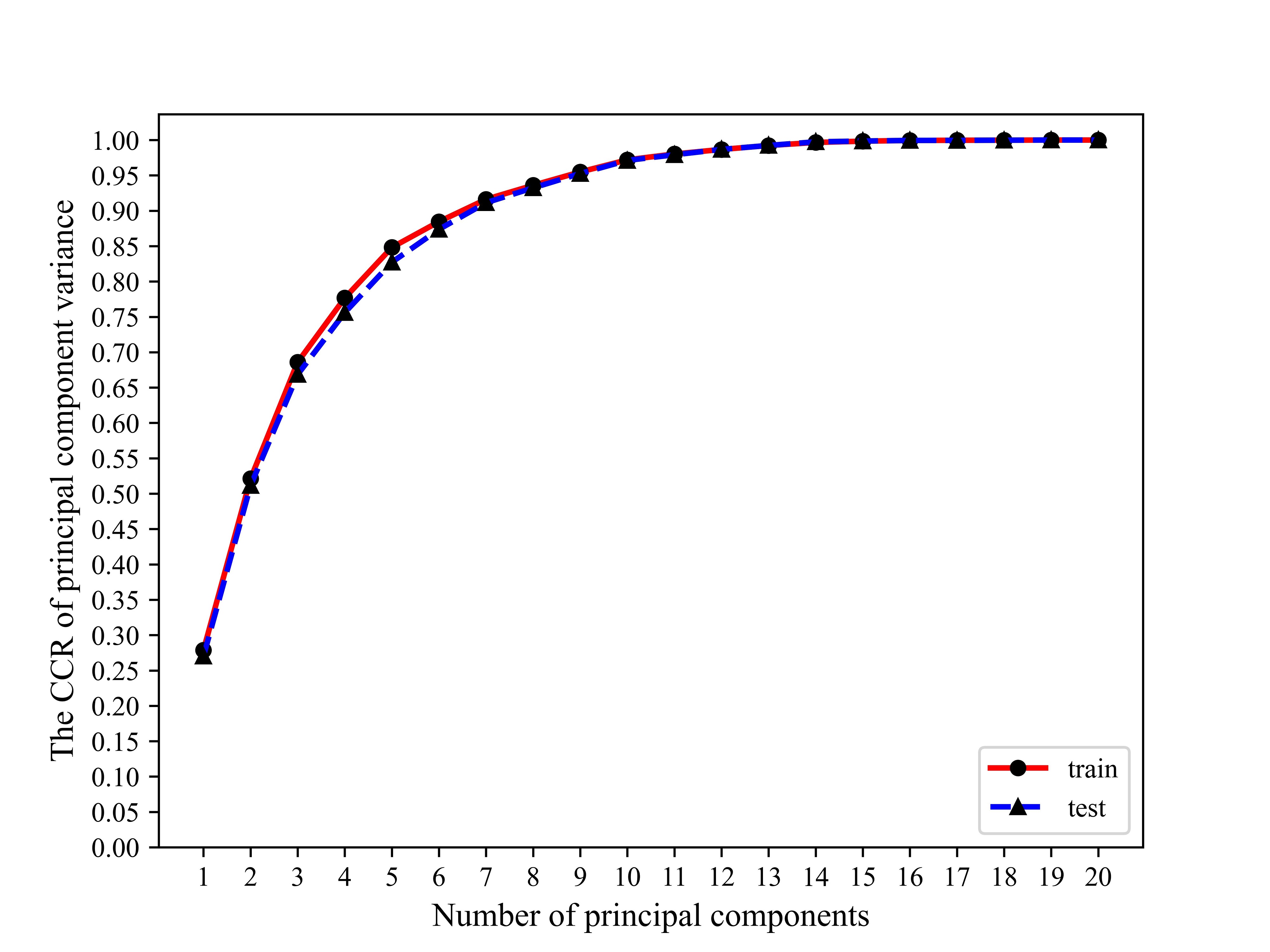

Supplement: Supplemental Information 1 — Model training results and comparison data. [file peerj-cs-10-1915-s001.zip › latex/4.12b.jpg]

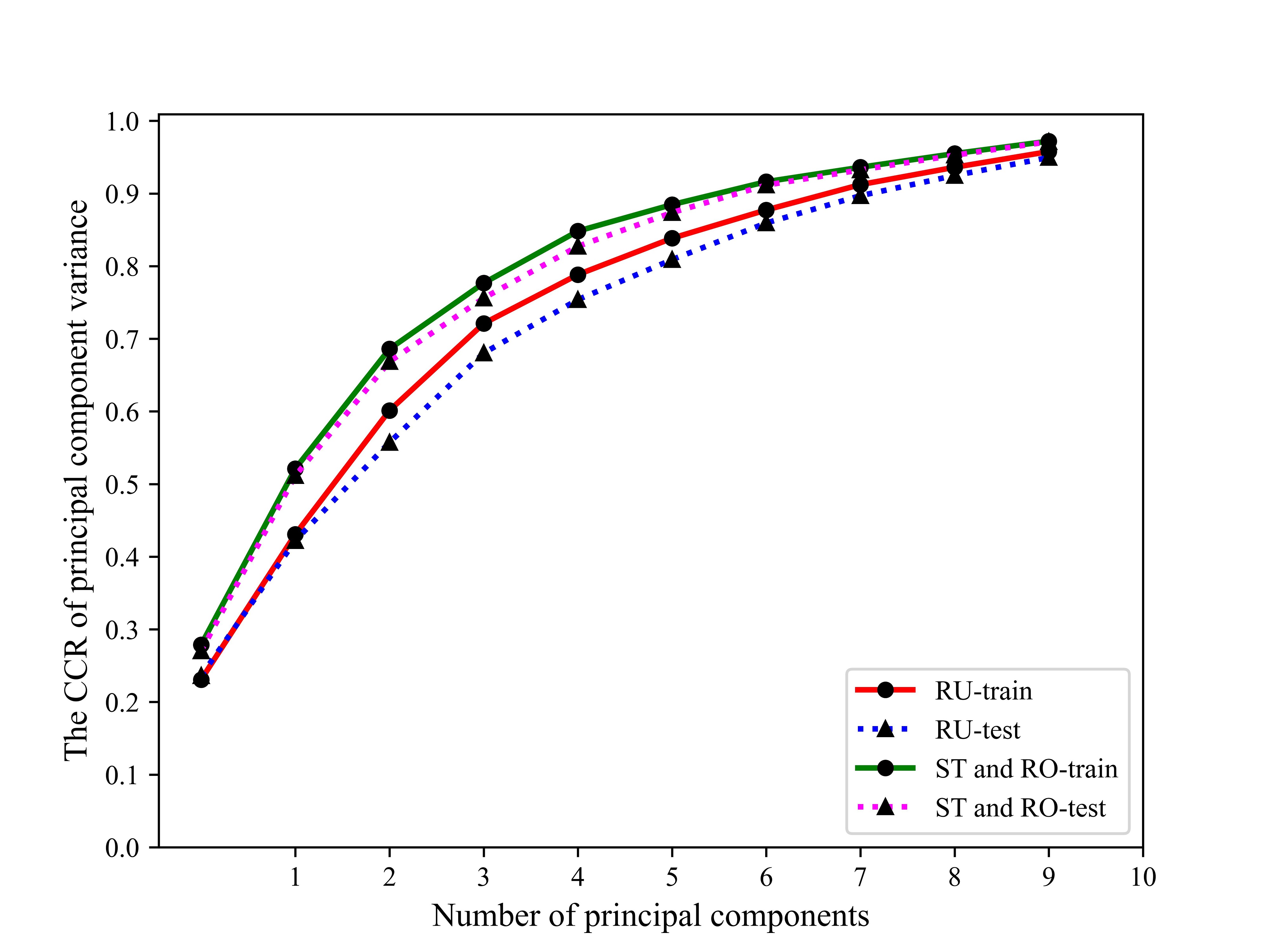

Supplement: Supplemental Information 1 — Model training results and comparison data. [file peerj-cs-10-1915-s001.zip › latex/4.13.jpg]

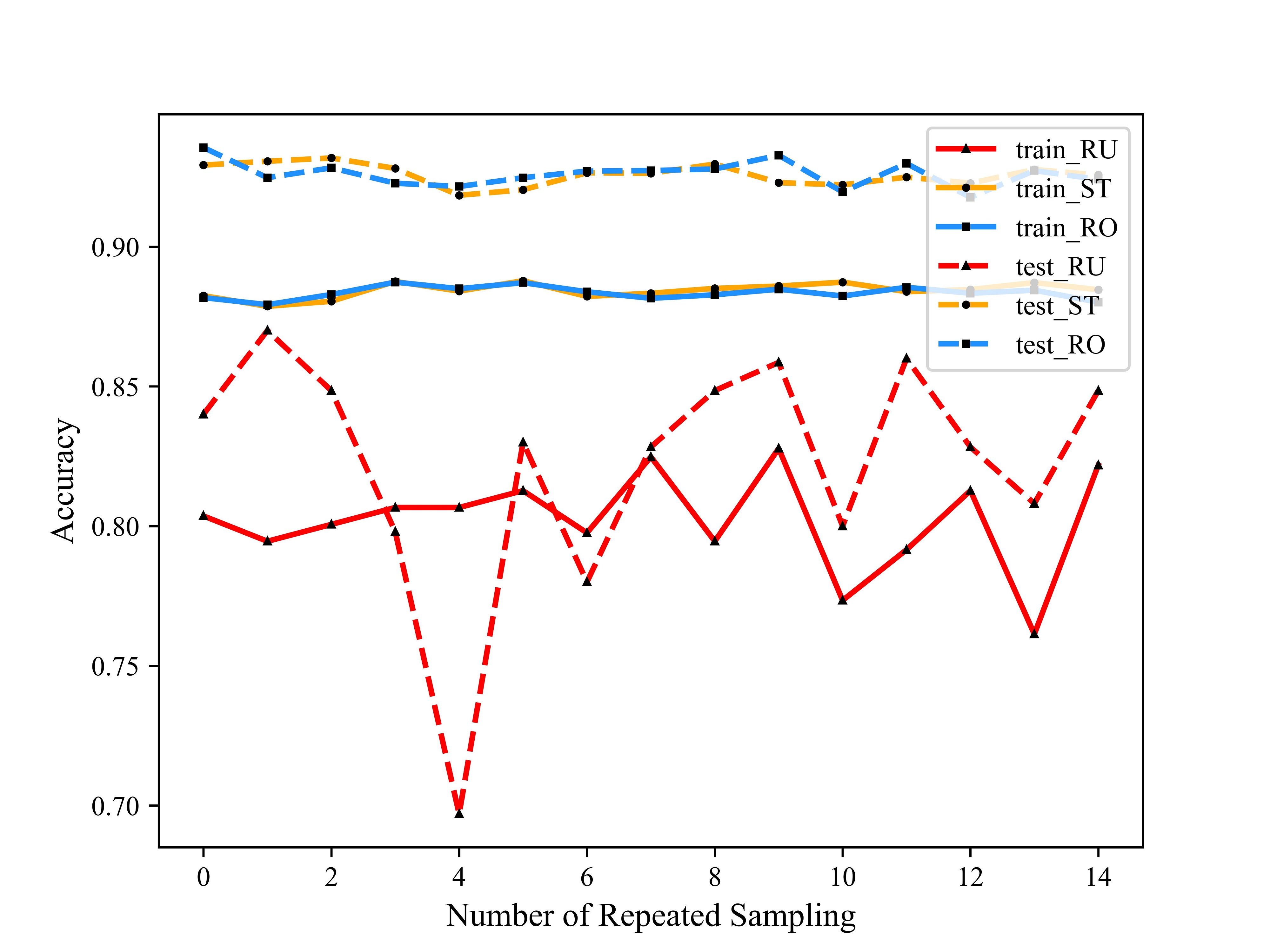

Supplement: Supplemental Information 1 — Model training results and comparison data. [file peerj-cs-10-1915-s001.zip › latex/4.14a.jpg]

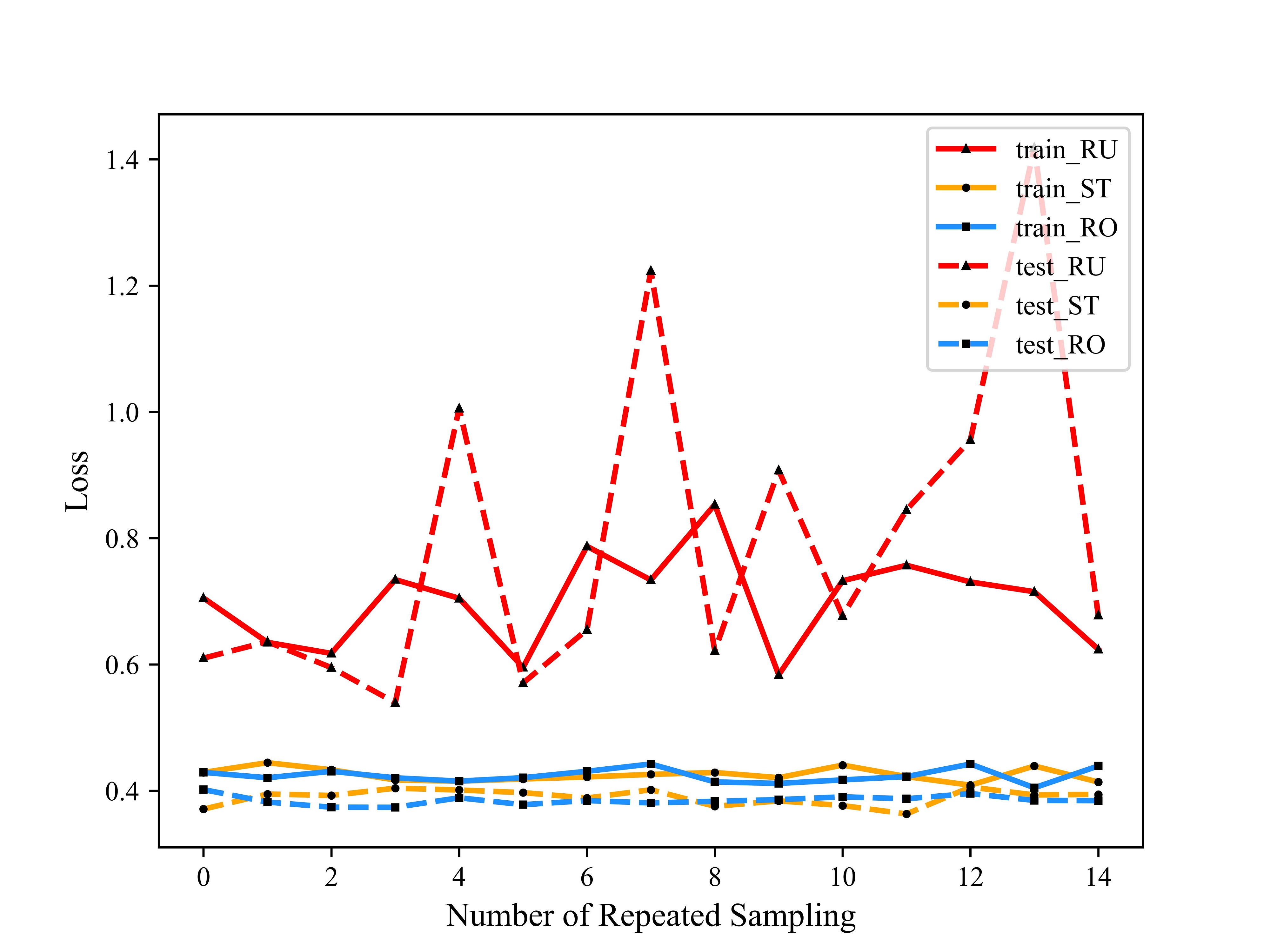

Supplement: Supplemental Information 1 — Model training results and comparison data. [file peerj-cs-10-1915-s001.zip › latex/4.14b.jpg]

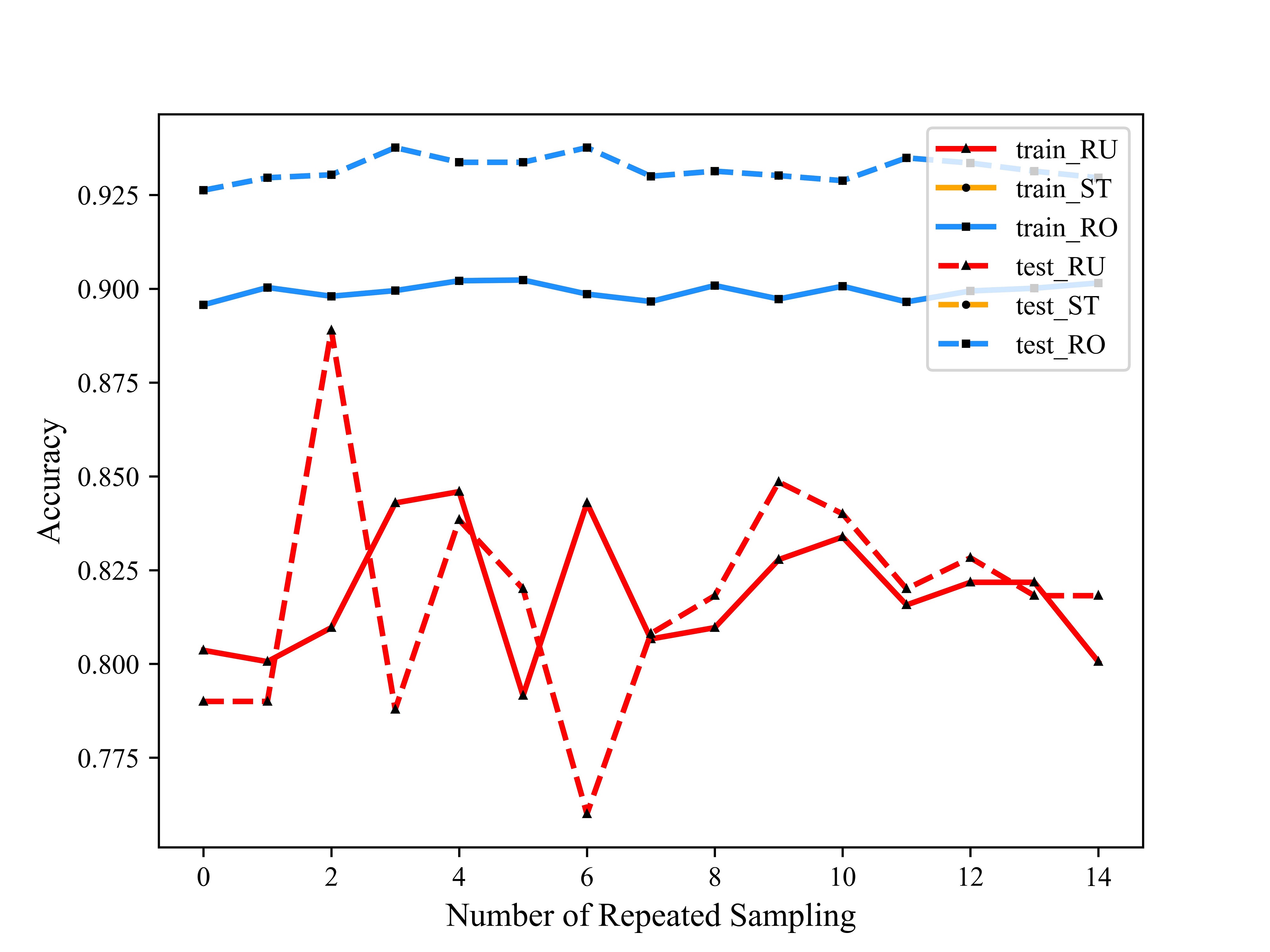

Supplement: Supplemental Information 1 — Model training results and comparison data. [file peerj-cs-10-1915-s001.zip › latex/4.15a.jpg]

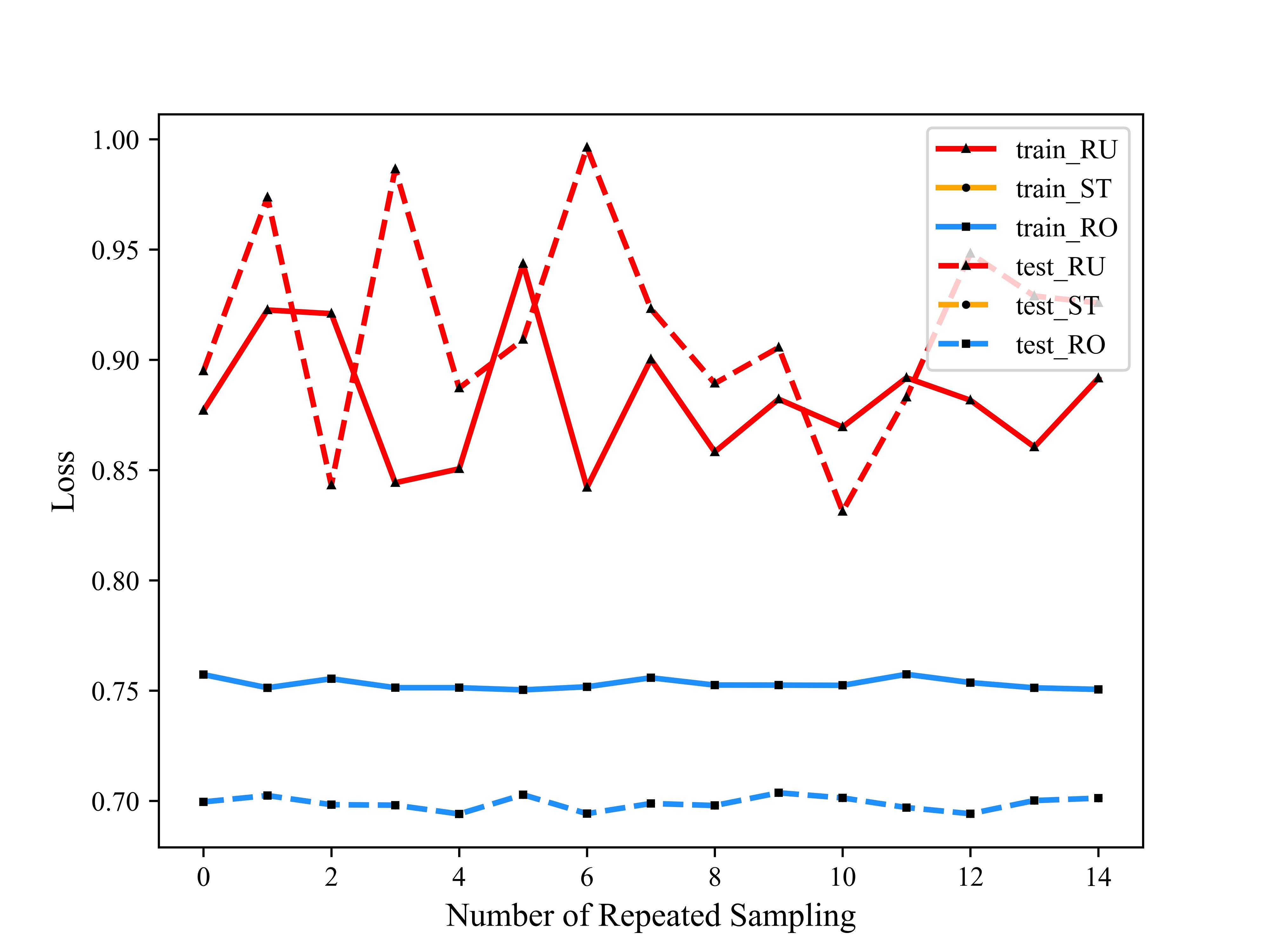

Supplement: Supplemental Information 1 — Model training results and comparison data. [file peerj-cs-10-1915-s001.zip › latex/4.15b.jpg]

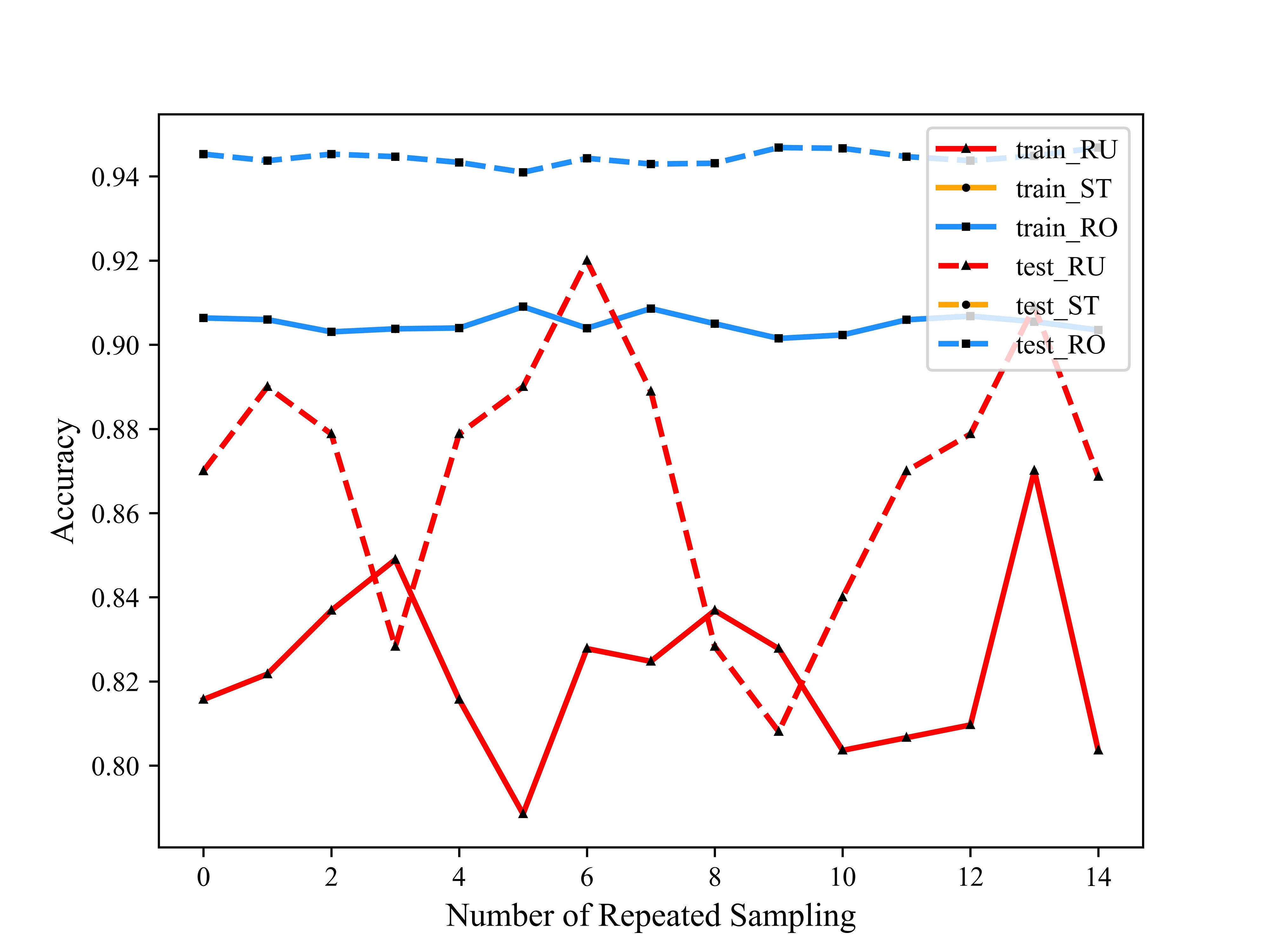

Supplement: Supplemental Information 1 — Model training results and comparison data. [file peerj-cs-10-1915-s001.zip › latex/4.16a.jpg]

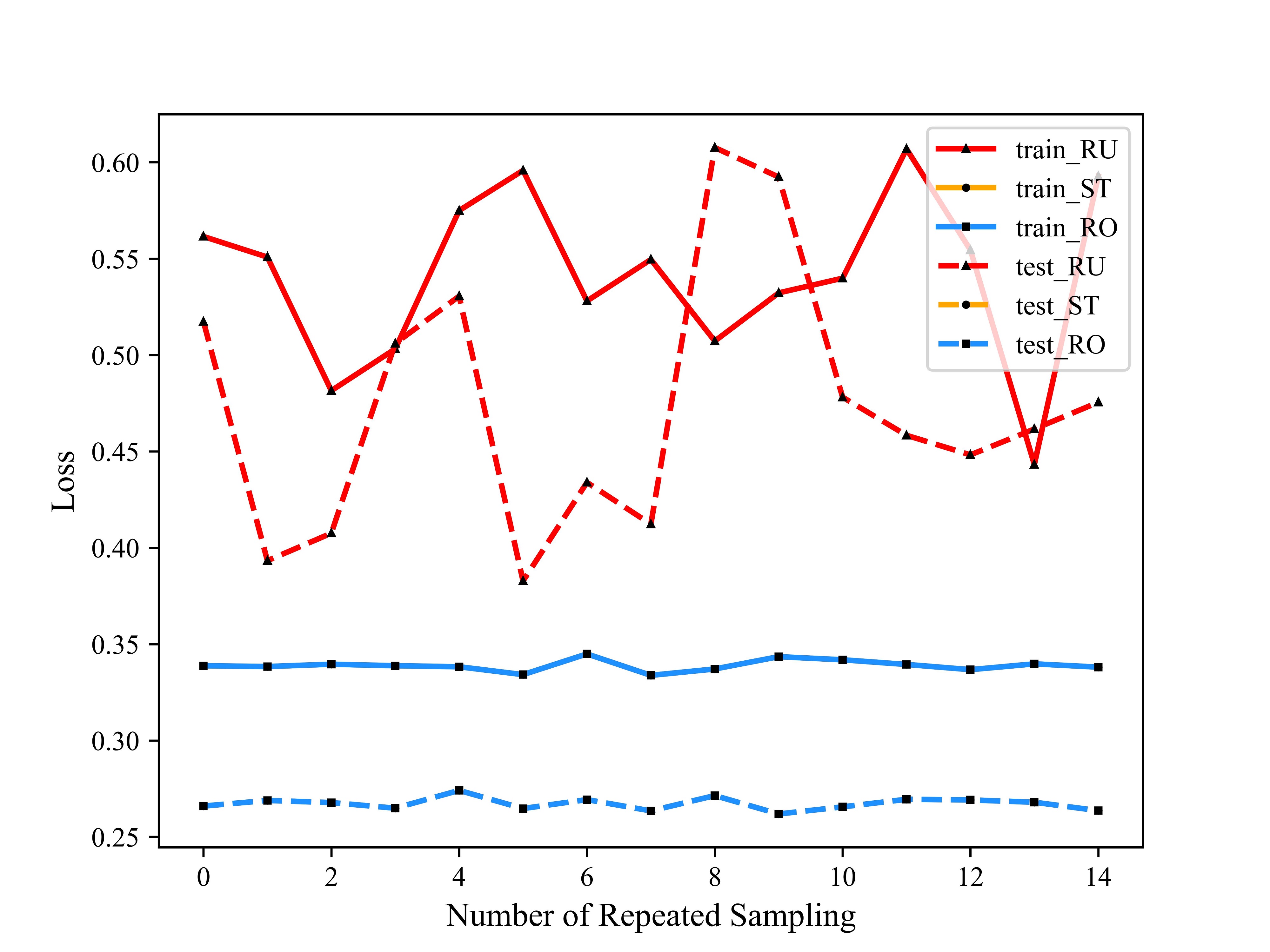

Supplement: Supplemental Information 1 — Model training results and comparison data. [file peerj-cs-10-1915-s001.zip › latex/4.16b.jpg]

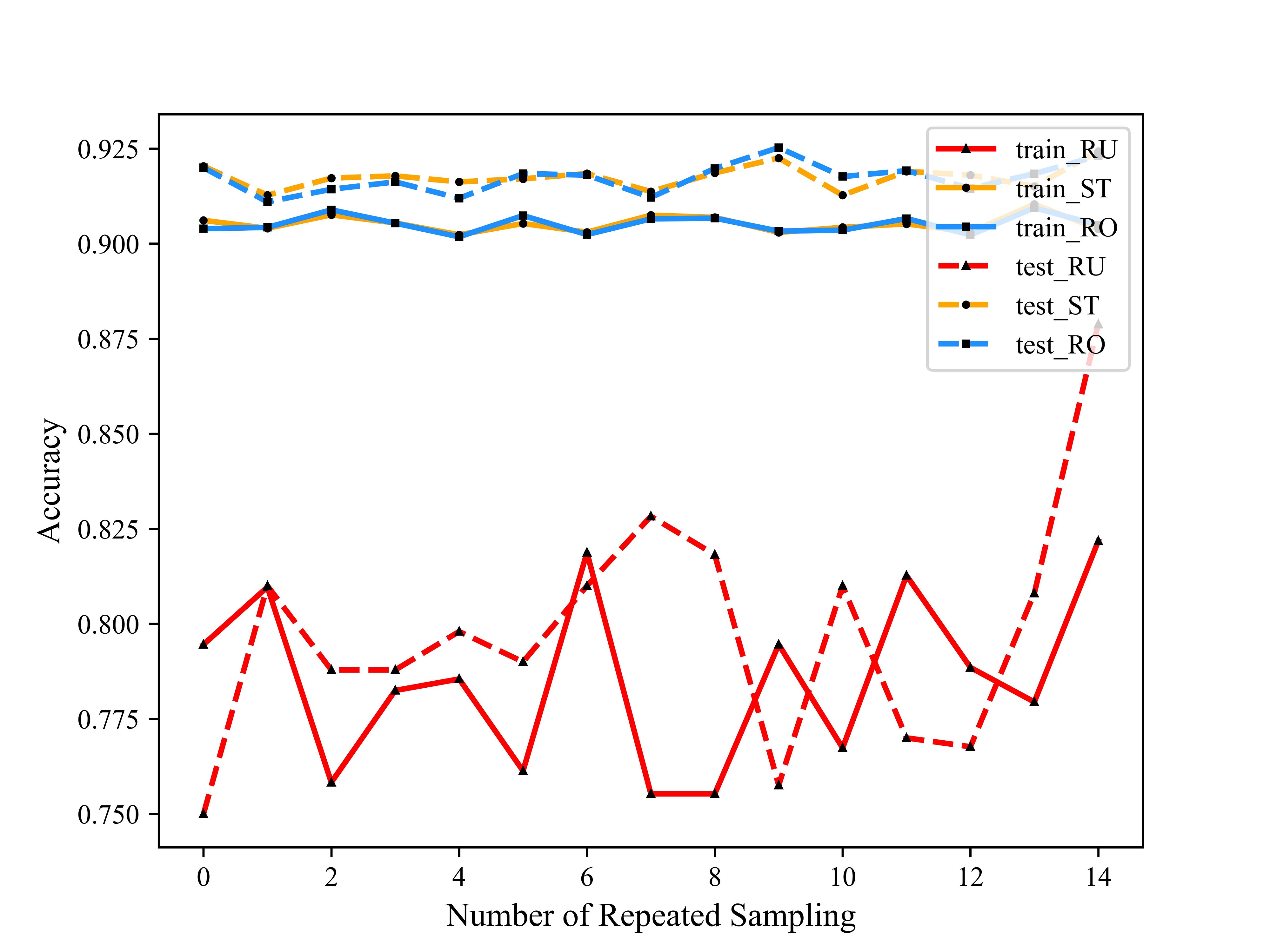

Supplement: Supplemental Information 1 — Model training results and comparison data. [file peerj-cs-10-1915-s001.zip › latex/4.17a.jpg]

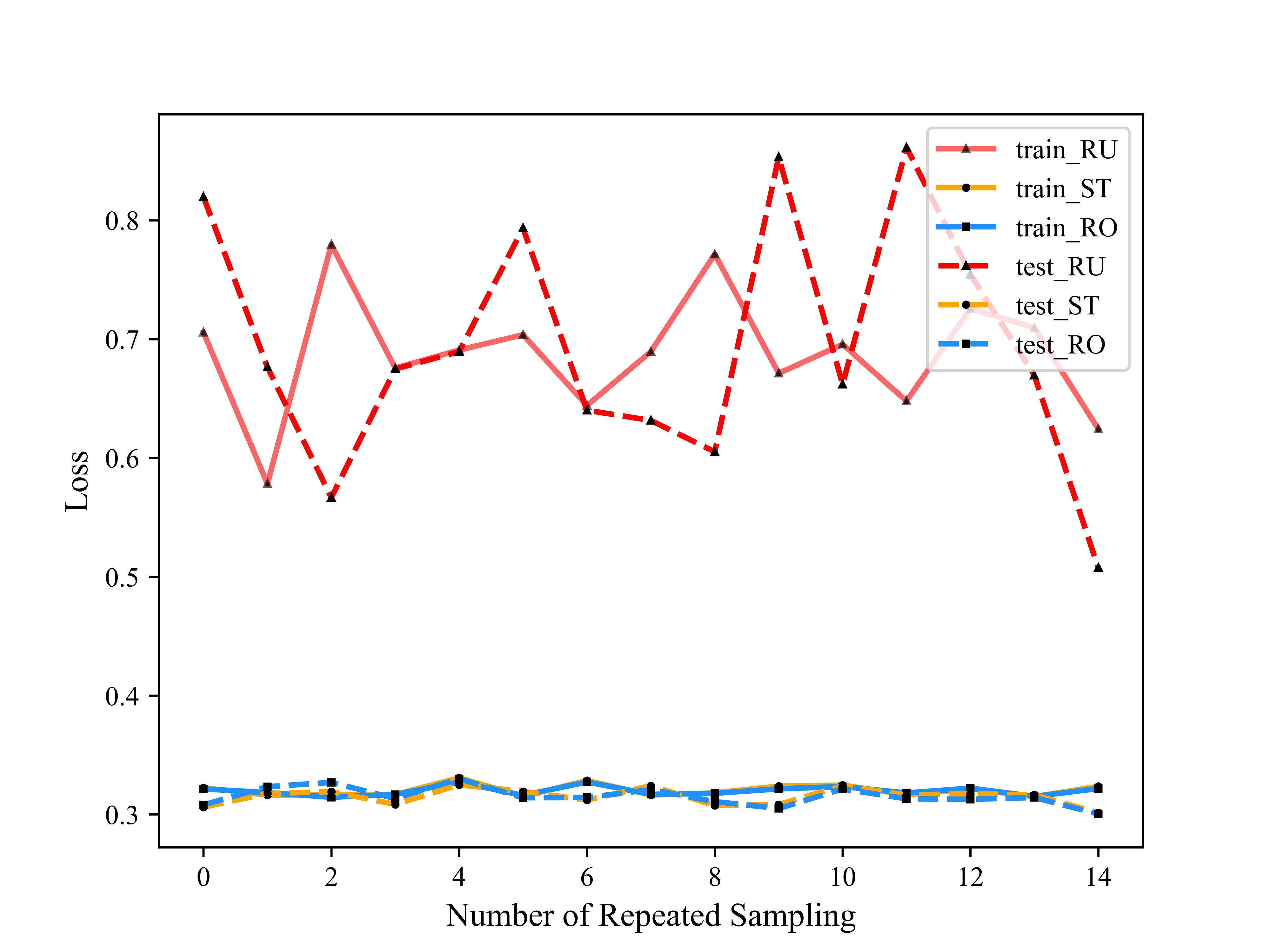

Supplement: Supplemental Information 1 — Model training results and comparison data. [file peerj-cs-10-1915-s001.zip › latex/4.17b.jpg]

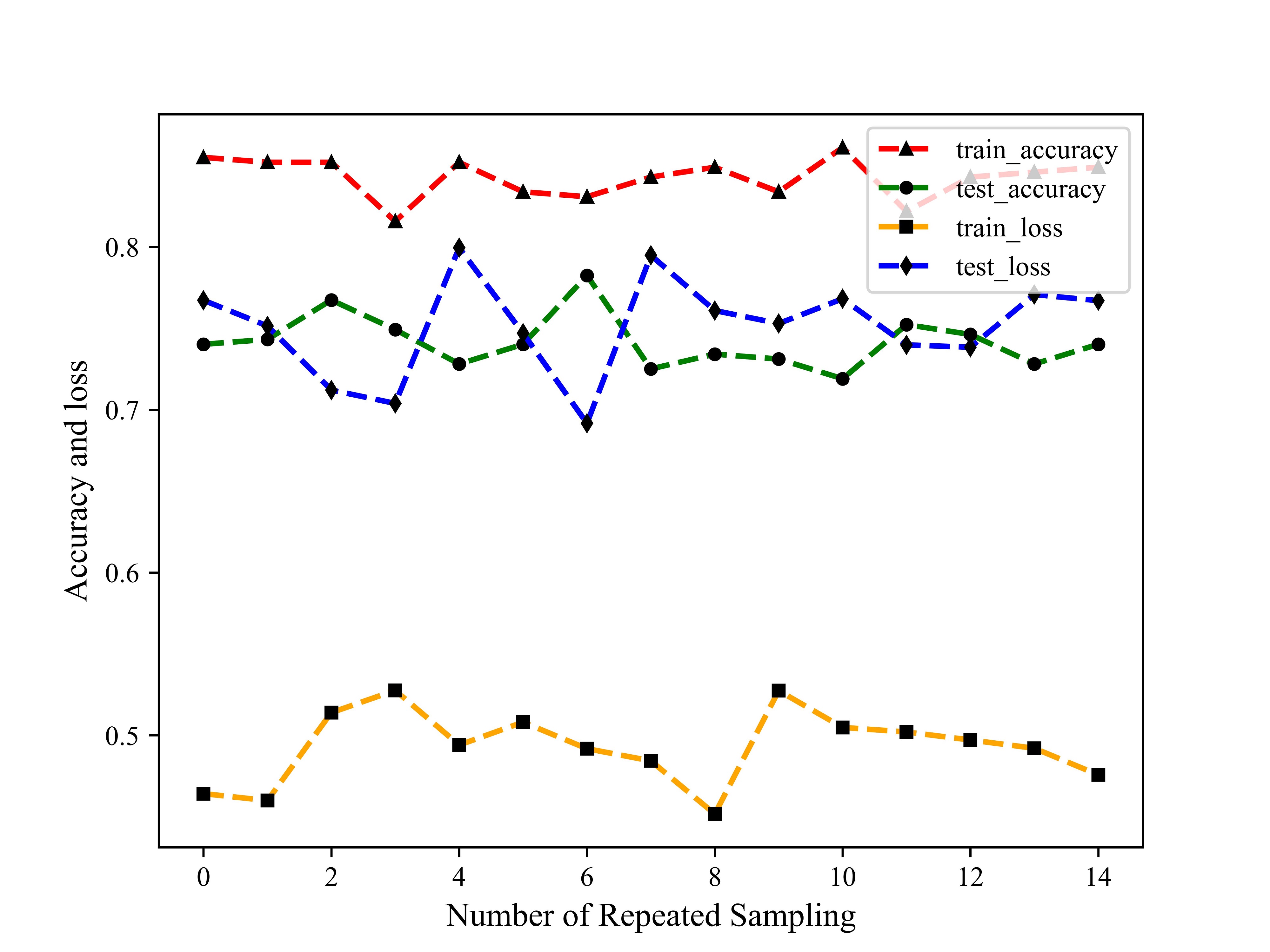

Supplement: Supplemental Information 1 — Model training results and comparison data. [file peerj-cs-10-1915-s001.zip › latex/4.18a.jpg]

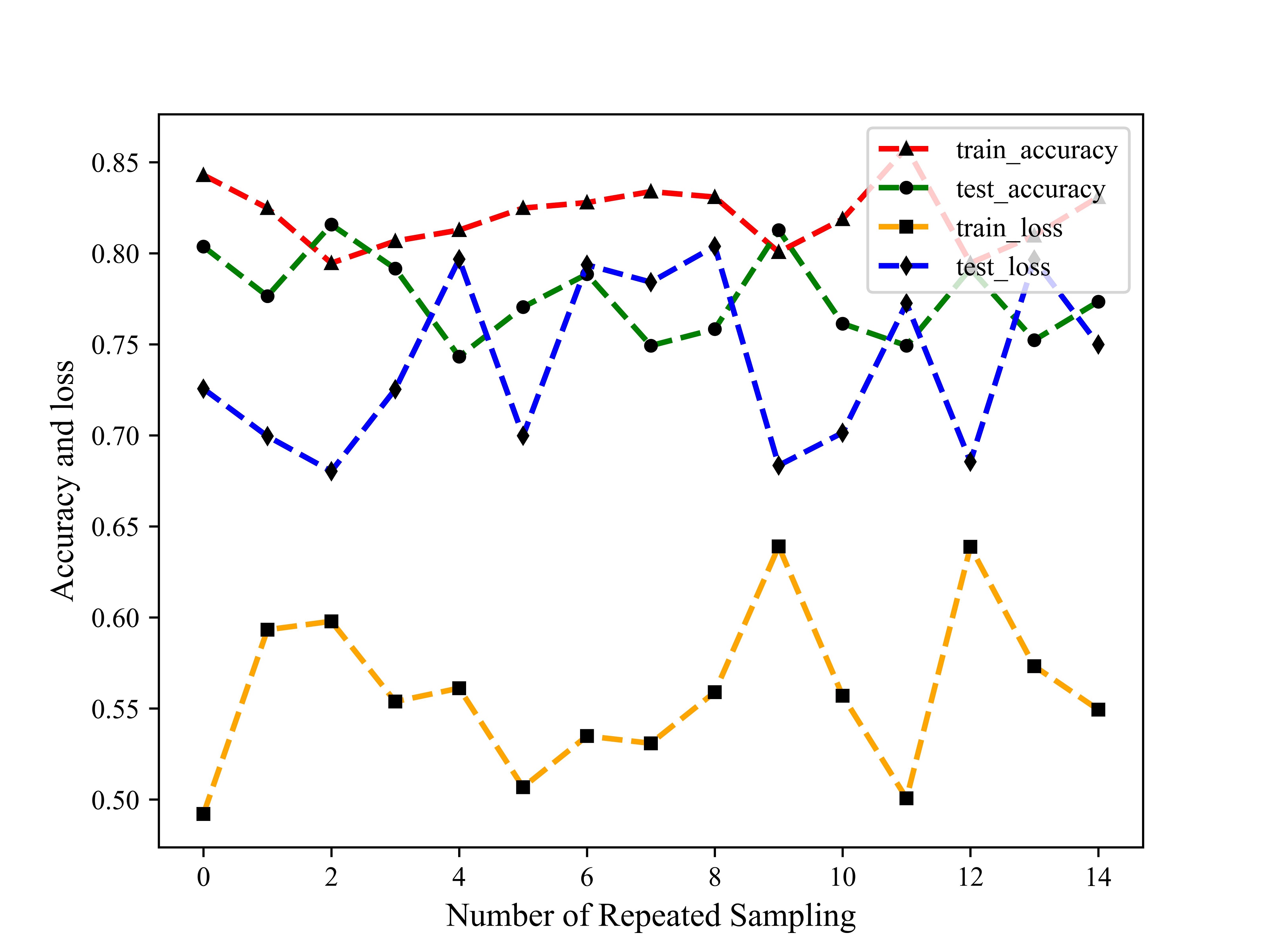

Supplement: Supplemental Information 1 — Model training results and comparison data. [file peerj-cs-10-1915-s001.zip › latex/4.18b.jpg]

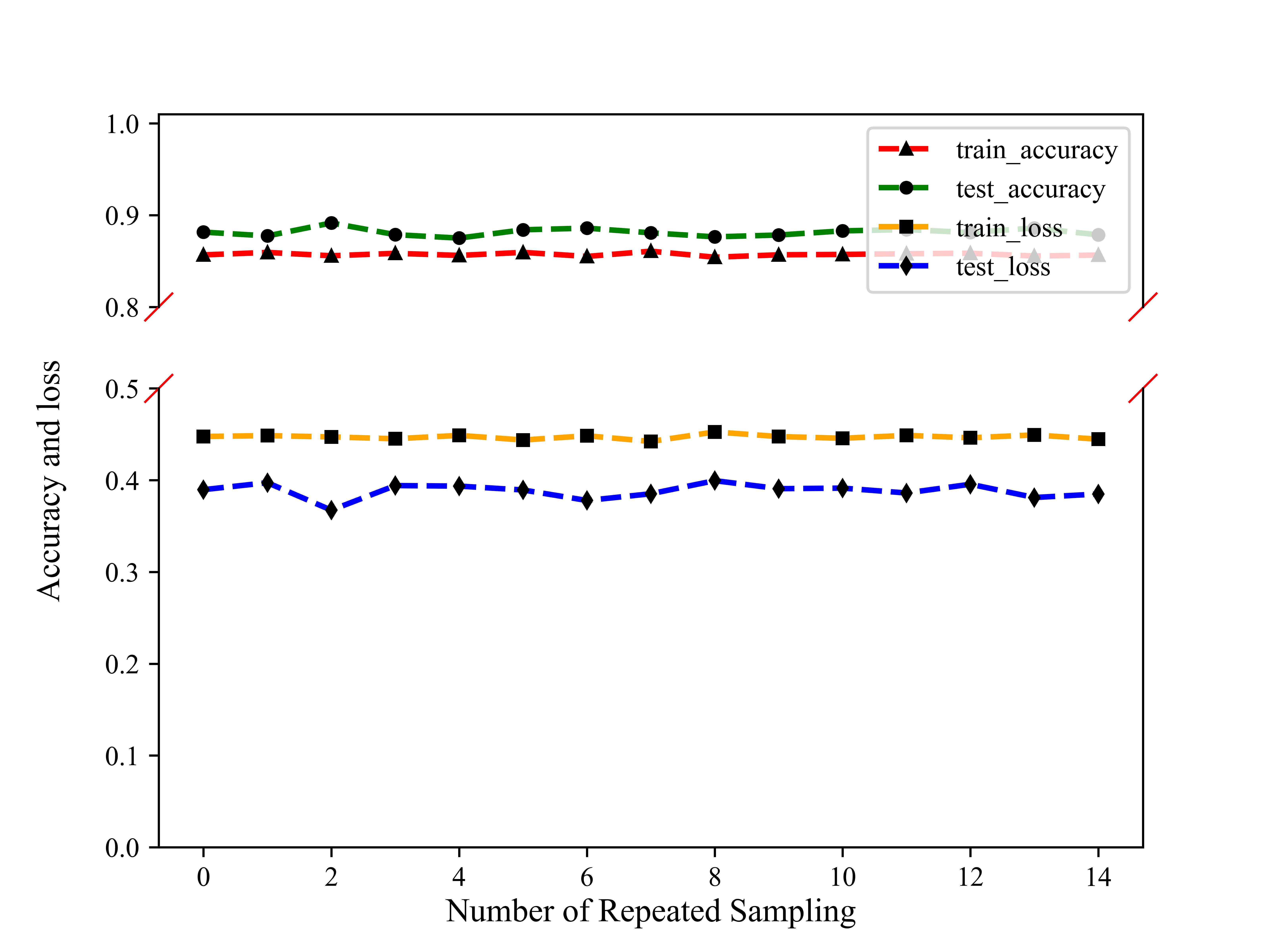

Supplement: Supplemental Information 1 — Model training results and comparison data. [file peerj-cs-10-1915-s001.zip › latex/4.19a.jpg]

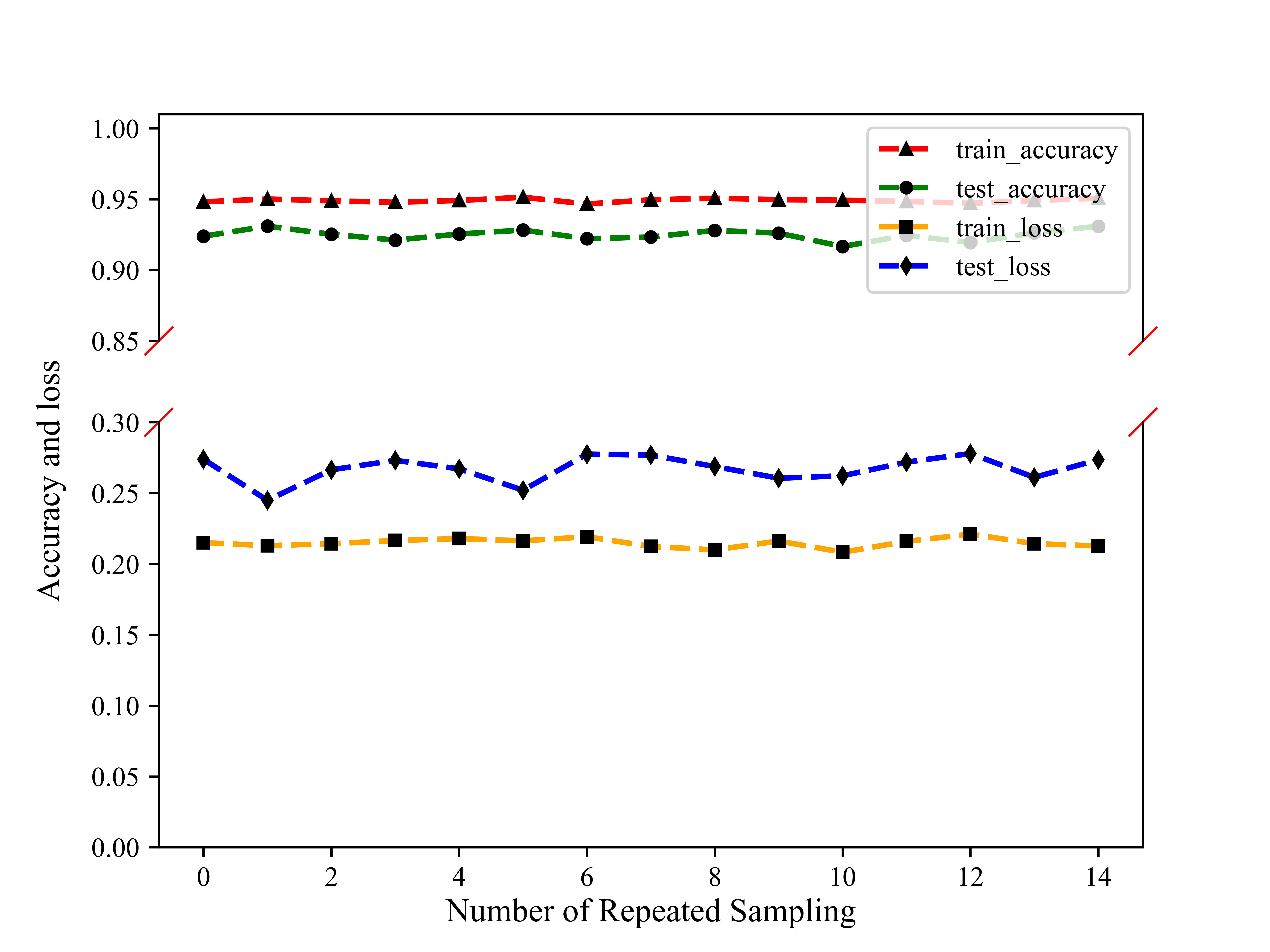

Supplement: Supplemental Information 1 — Model training results and comparison data. [file peerj-cs-10-1915-s001.zip › latex/4.19b.jpg]

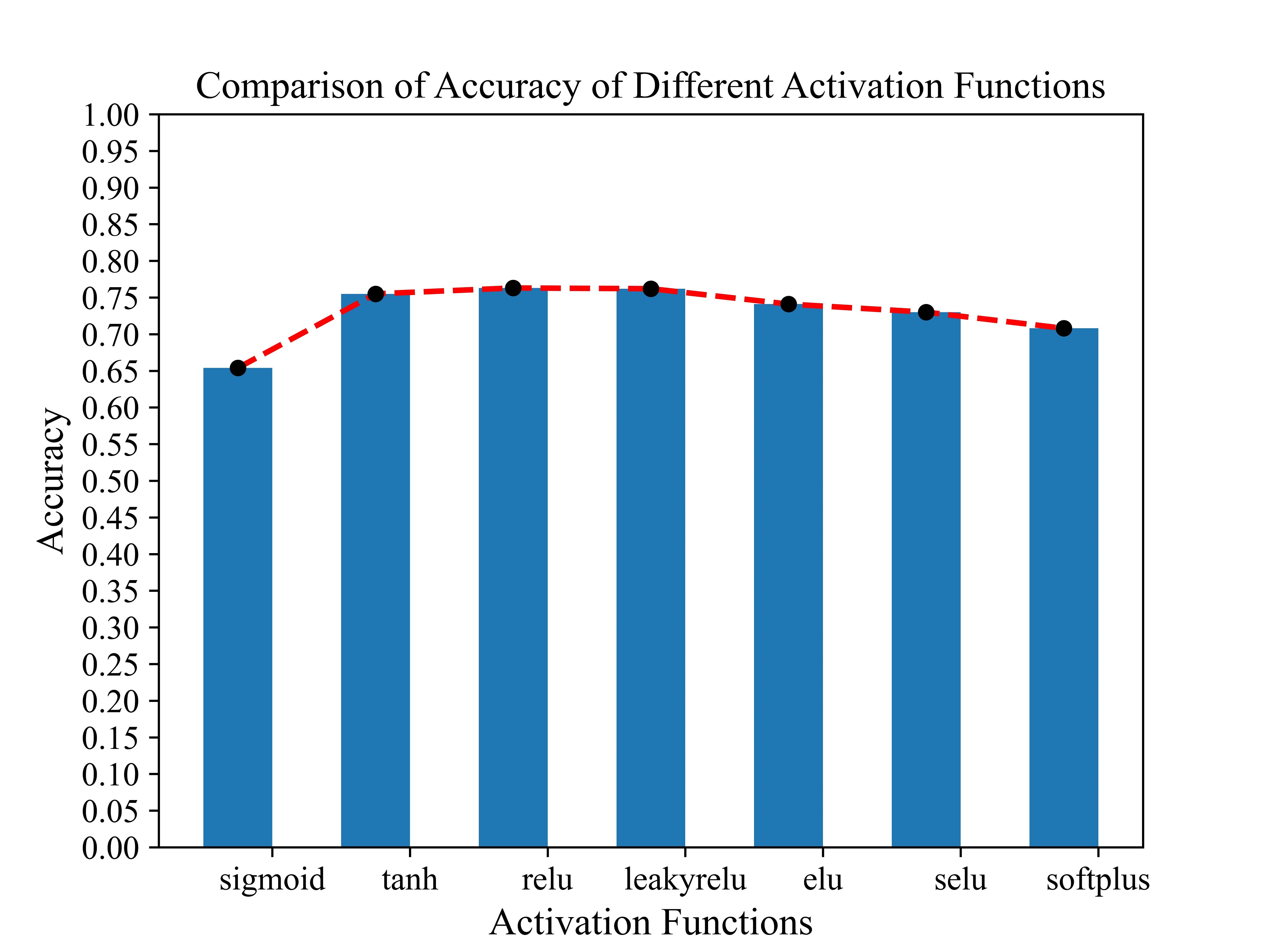

Supplement: Supplemental Information 1 — Model training results and comparison data. [file peerj-cs-10-1915-s001.zip › latex/4.2.jpg]

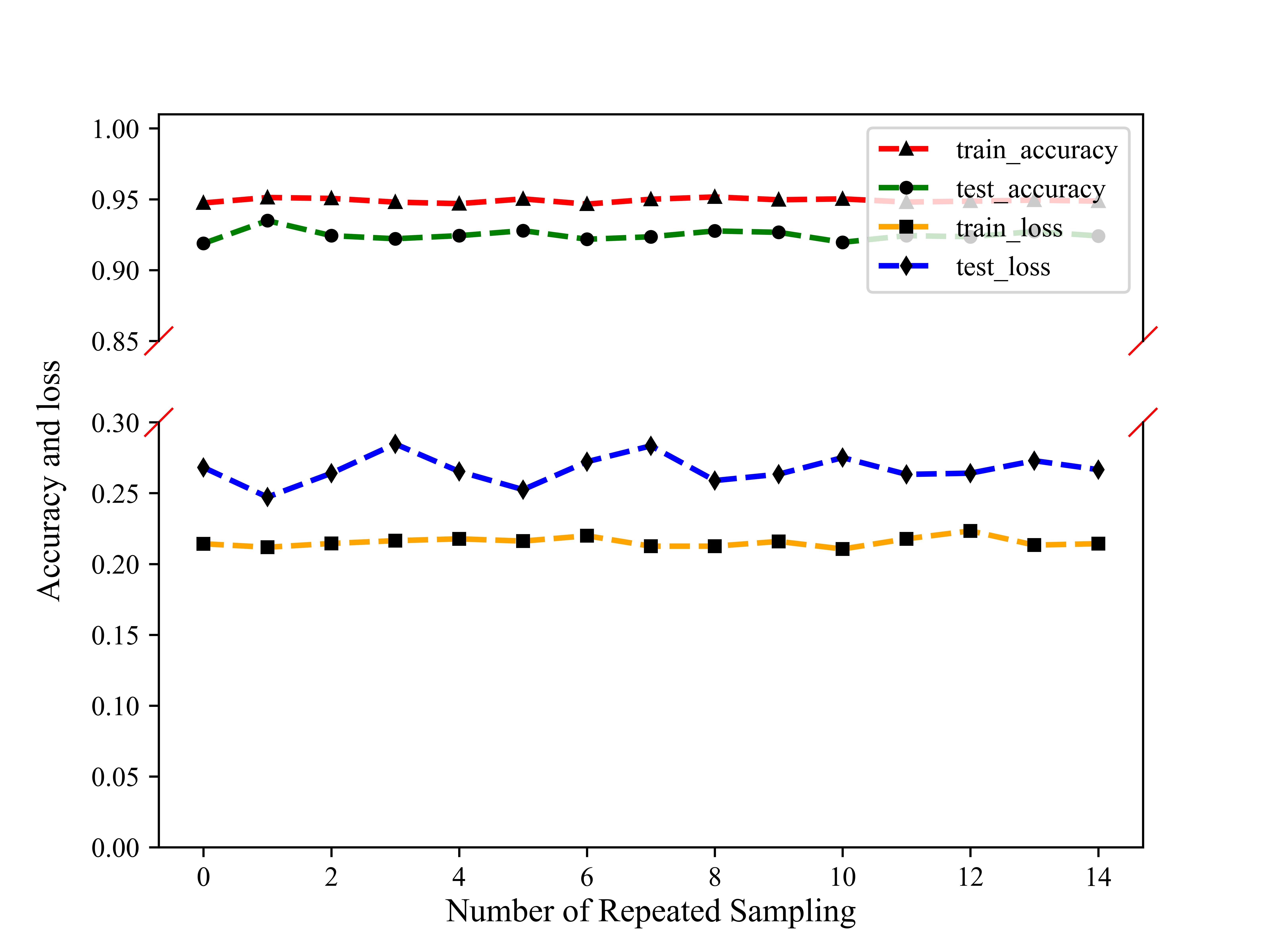

Supplement: Supplemental Information 1 — Model training results and comparison data. [file peerj-cs-10-1915-s001.zip › latex/4.20b.jpg]

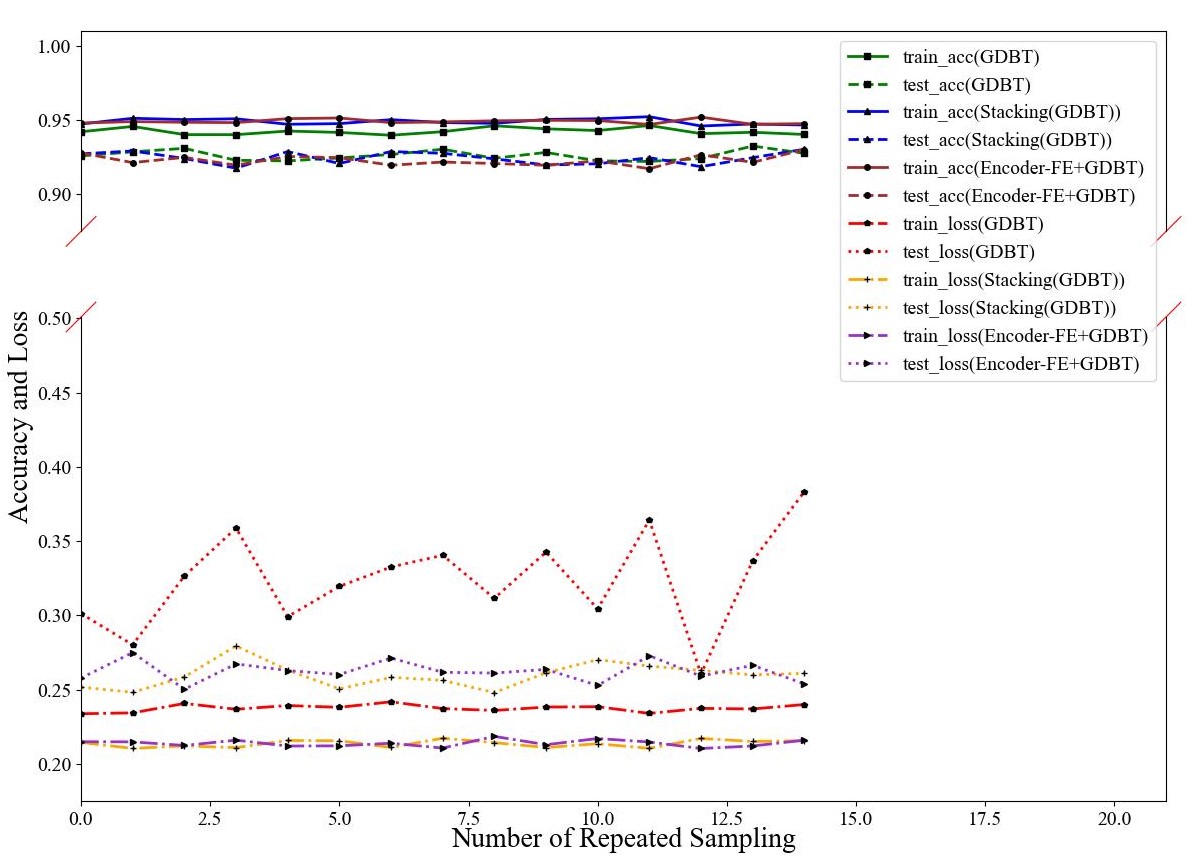

Supplement: Supplemental Information 1 — Model training results and comparison data. [file peerj-cs-10-1915-s001.zip › latex/4.21a.jpg]

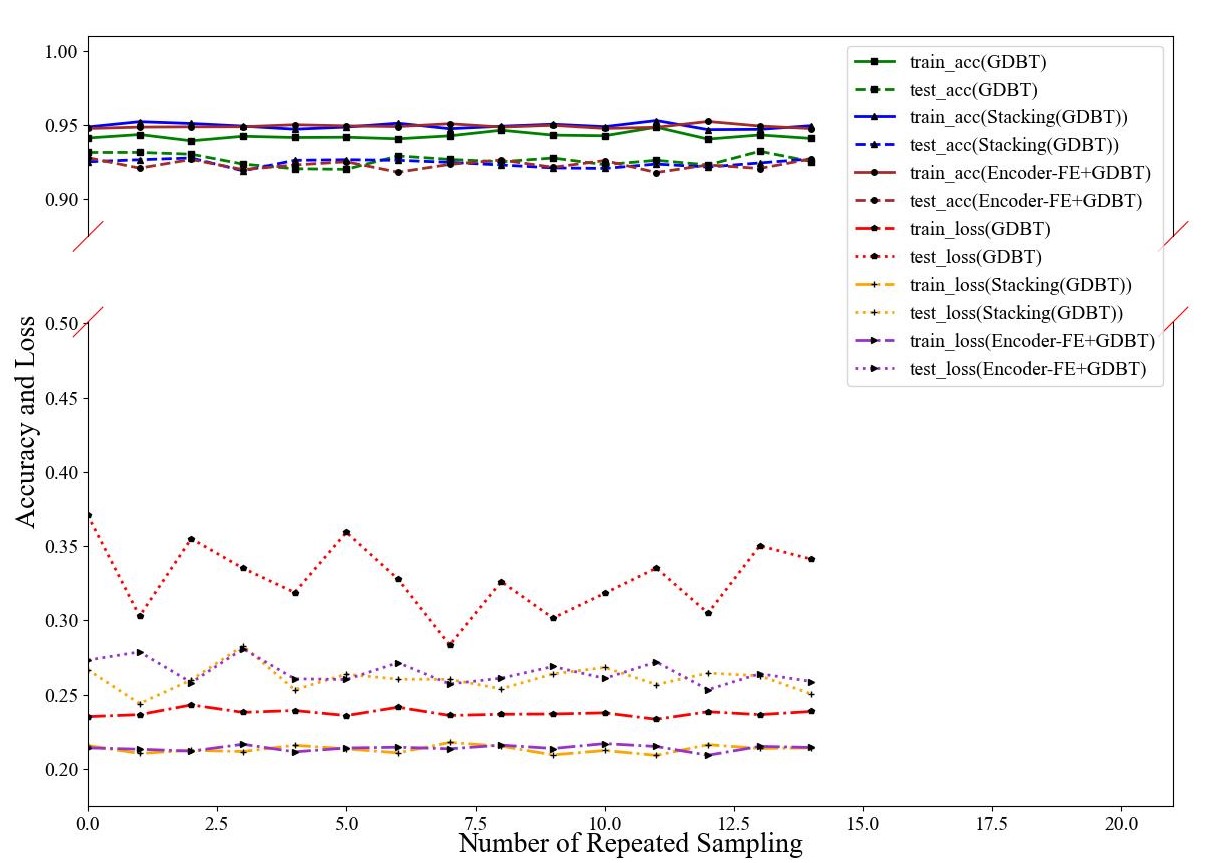

Supplement: Supplemental Information 1 — Model training results and comparison data. [file peerj-cs-10-1915-s001.zip › latex/4.21b.jpg]

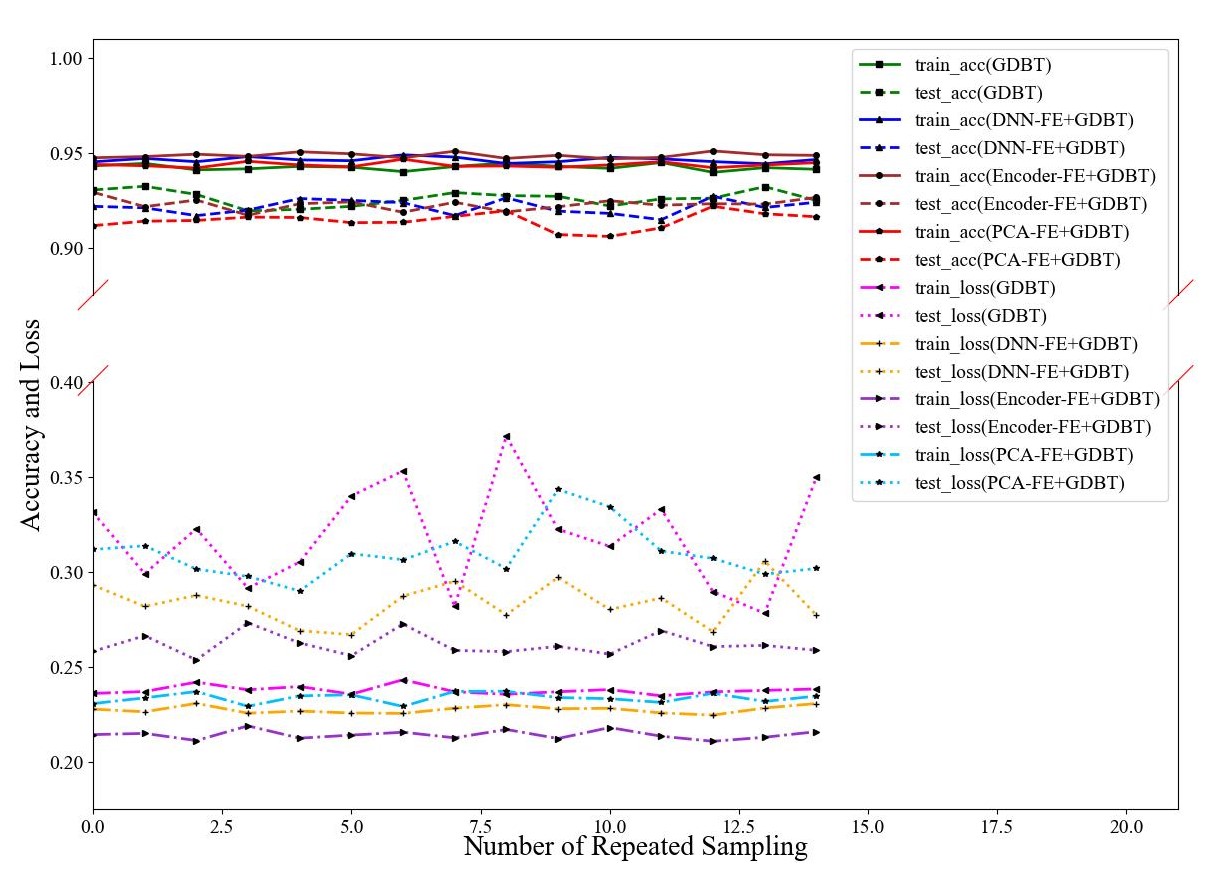

Supplement: Supplemental Information 1 — Model training results and comparison data. [file peerj-cs-10-1915-s001.zip › latex/4.22a.jpg]

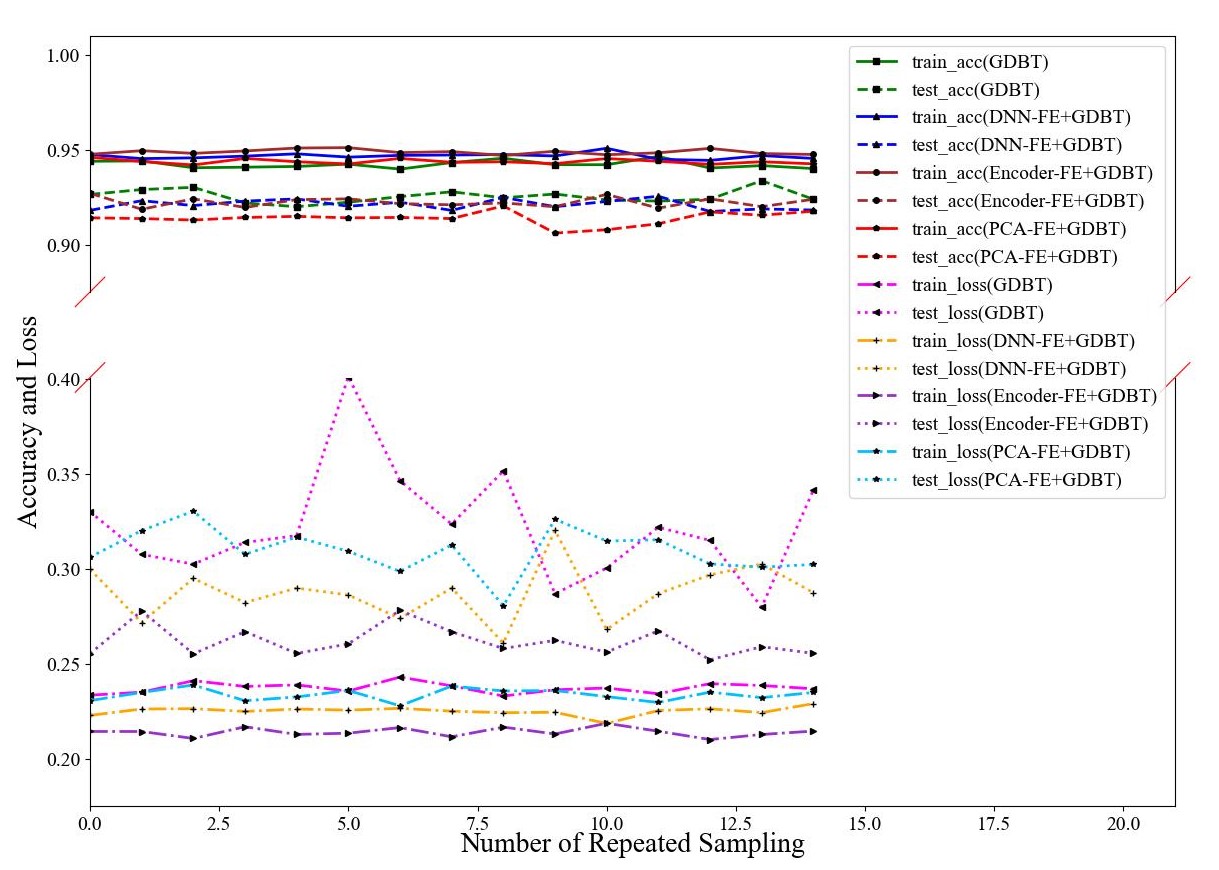

Supplement: Supplemental Information 1 — Model training results and comparison data. [file peerj-cs-10-1915-s001.zip › latex/4.22b.jpg]

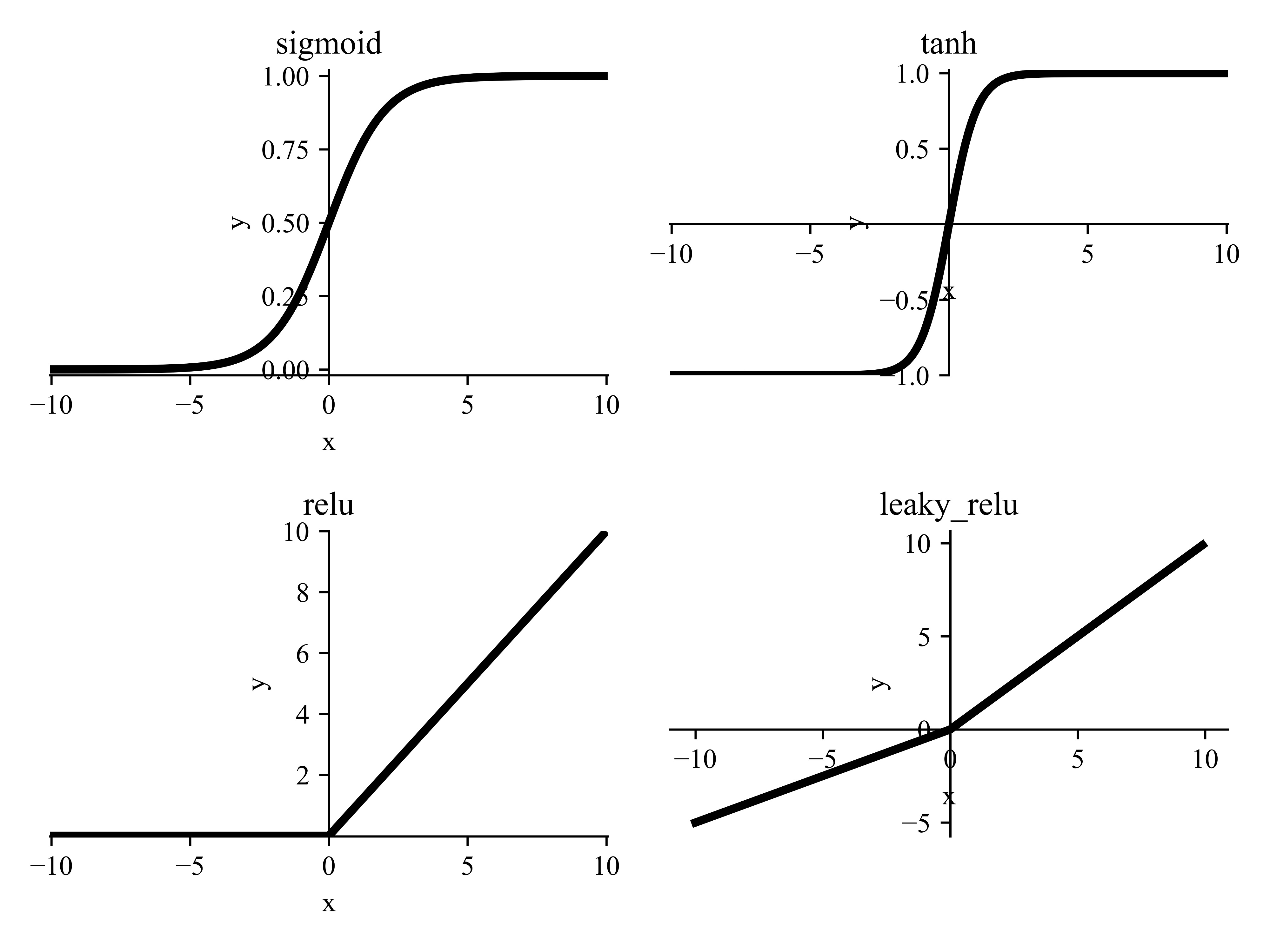

Supplement: Supplemental Information 1 — Model training results and comparison data. [file peerj-cs-10-1915-s001.zip › latex/4.3.jpg]

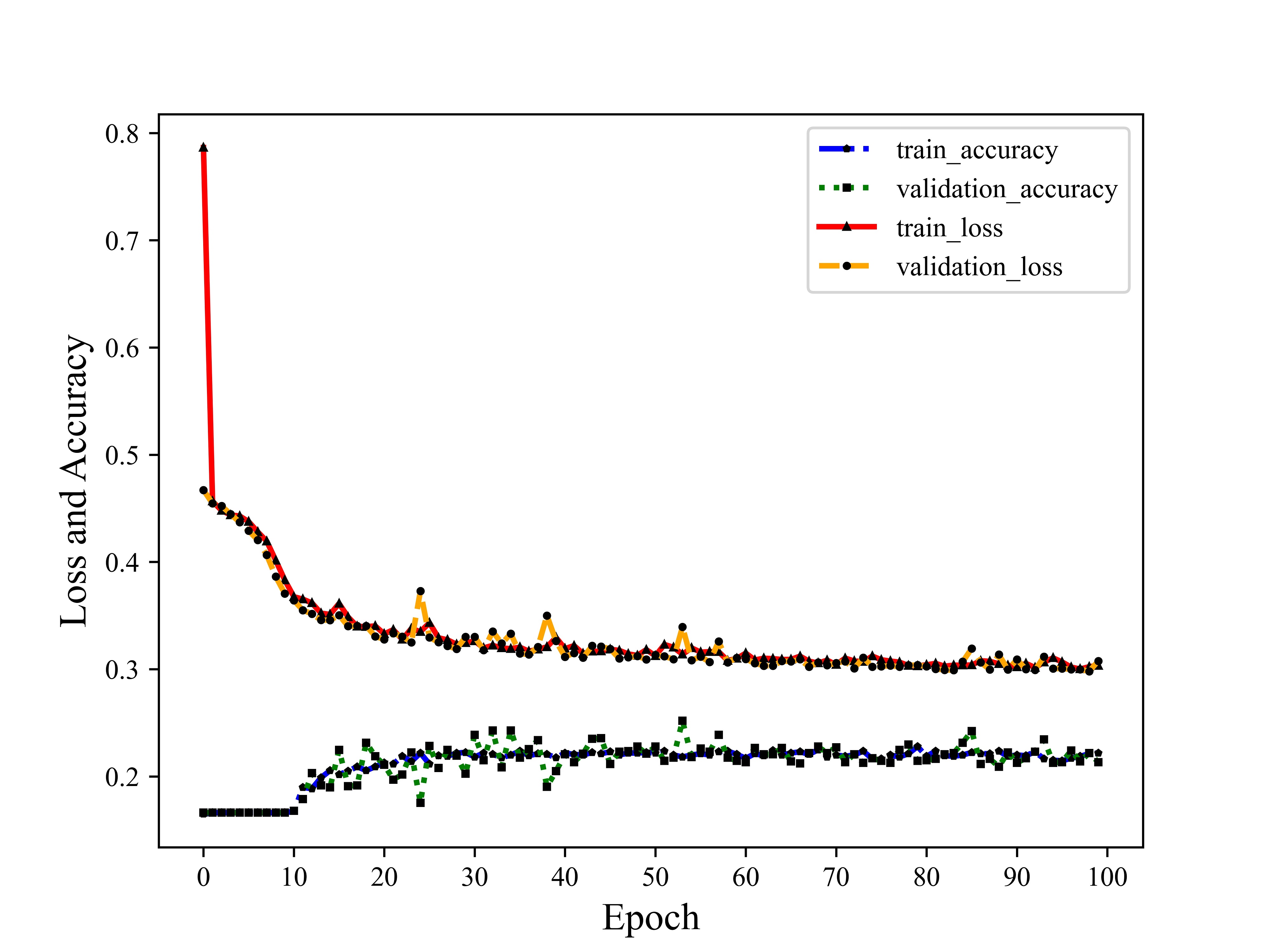

Supplement: Supplemental Information 1 — Model training results and comparison data. [file peerj-cs-10-1915-s001.zip › latex/4.4a.jpg]

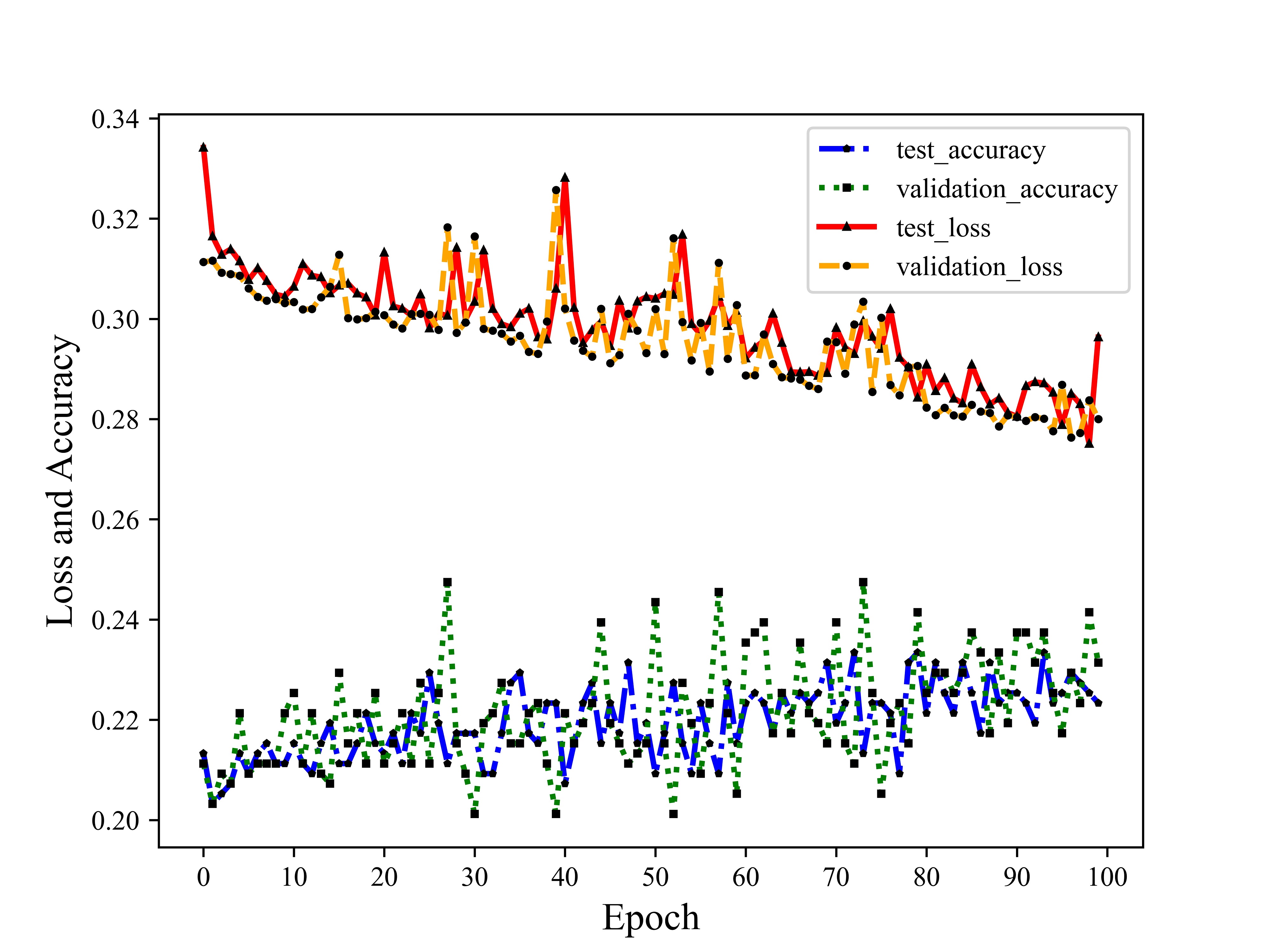

Supplement: Supplemental Information 1 — Model training results and comparison data. [file peerj-cs-10-1915-s001.zip › latex/4.4b.jpg]

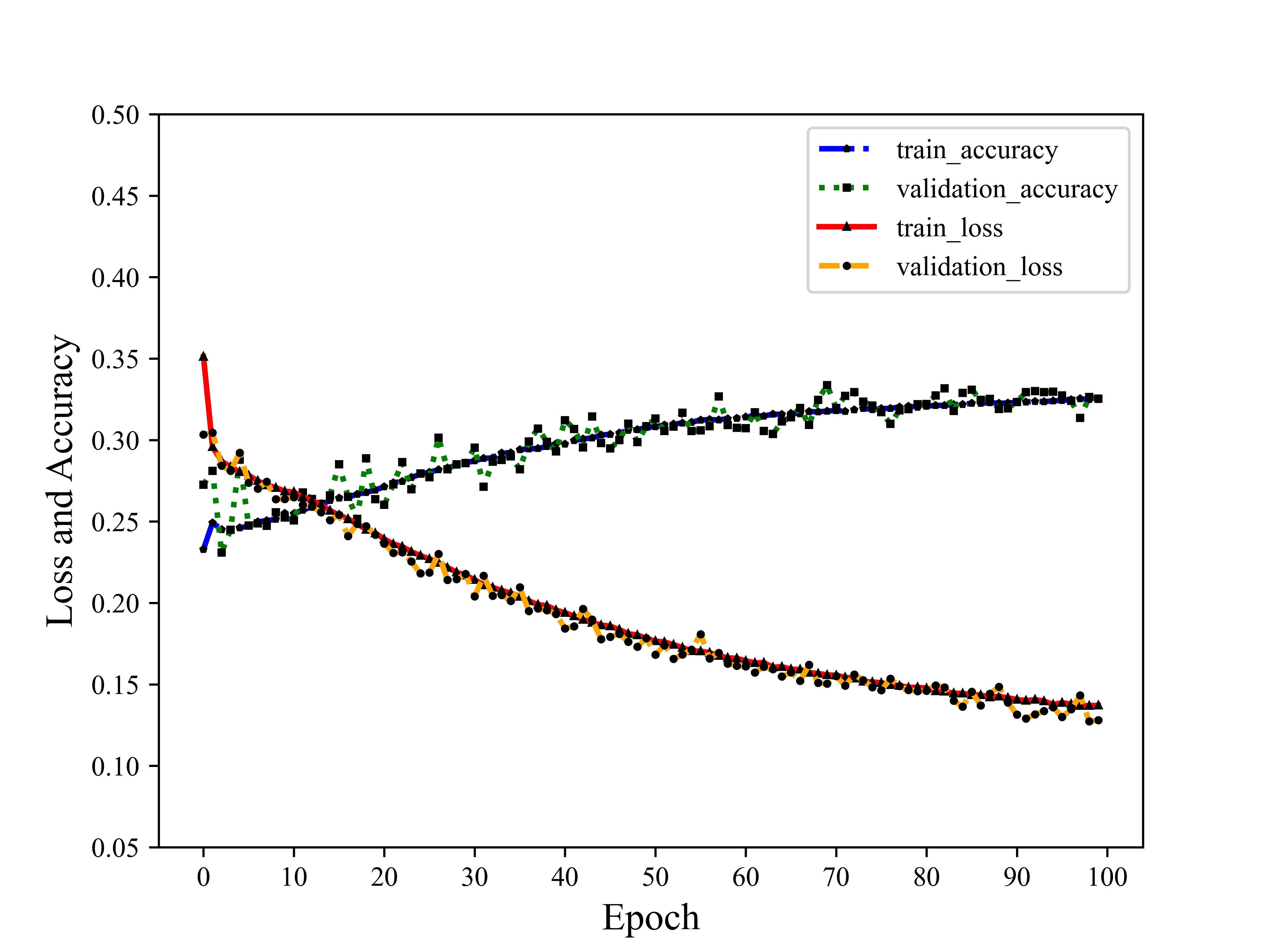

Supplement: Supplemental Information 1 — Model training results and comparison data. [file peerj-cs-10-1915-s001.zip › latex/4.5a.jpg]

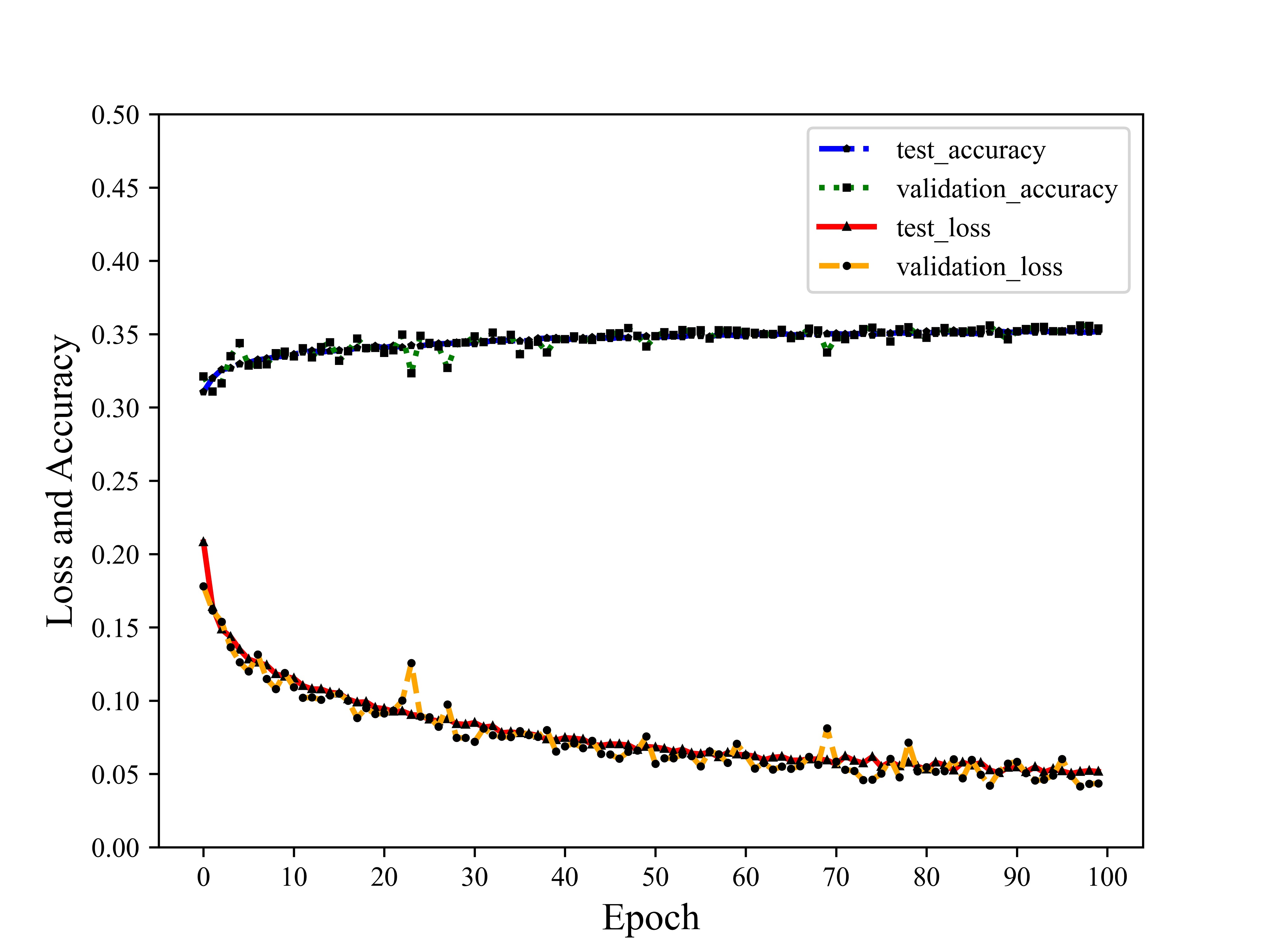

Supplement: Supplemental Information 1 — Model training results and comparison data. [file peerj-cs-10-1915-s001.zip › latex/4.5b.jpg]

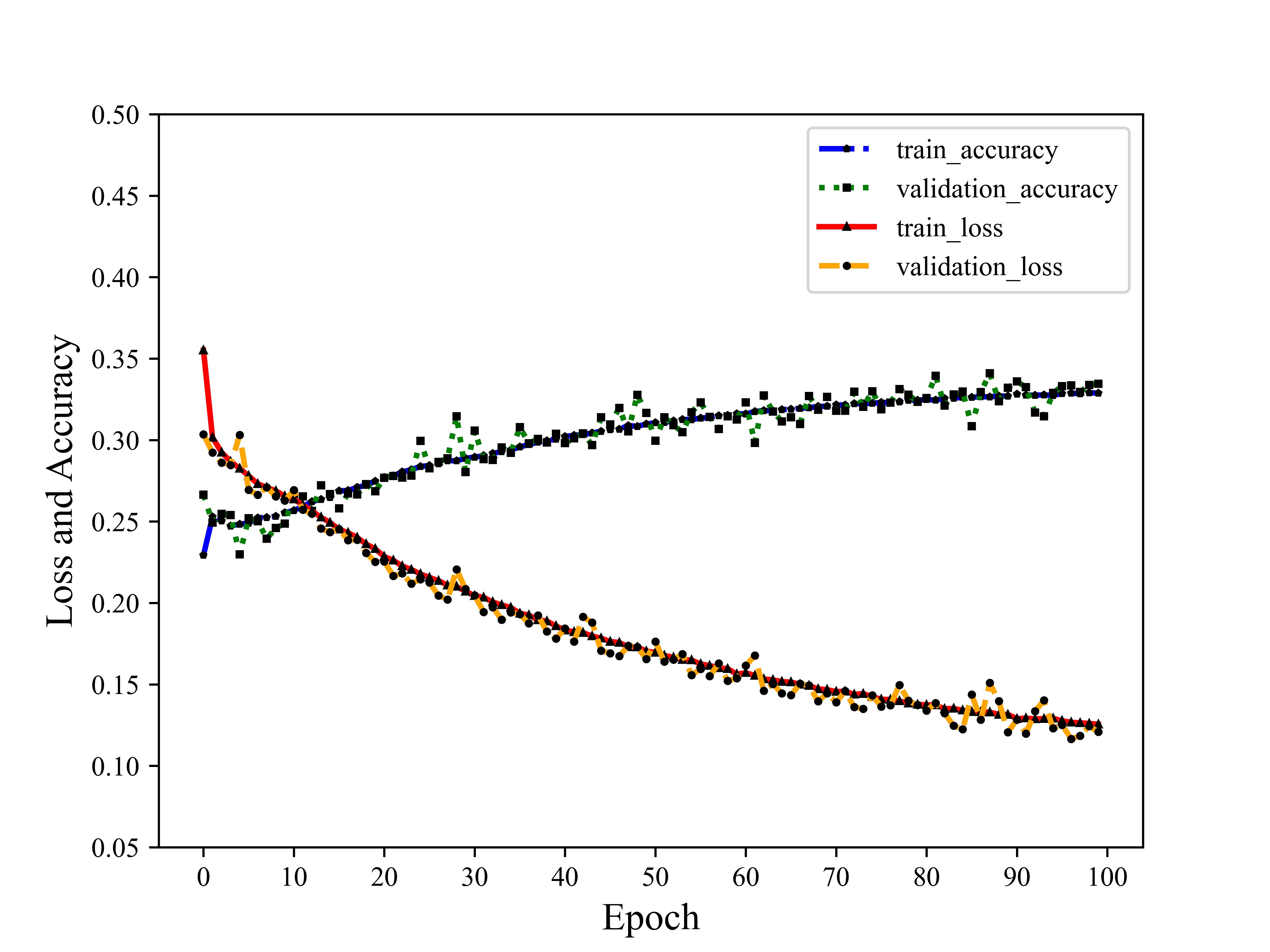

Supplement: Supplemental Information 1 — Model training results and comparison data. [file peerj-cs-10-1915-s001.zip › latex/4.6a.jpg]

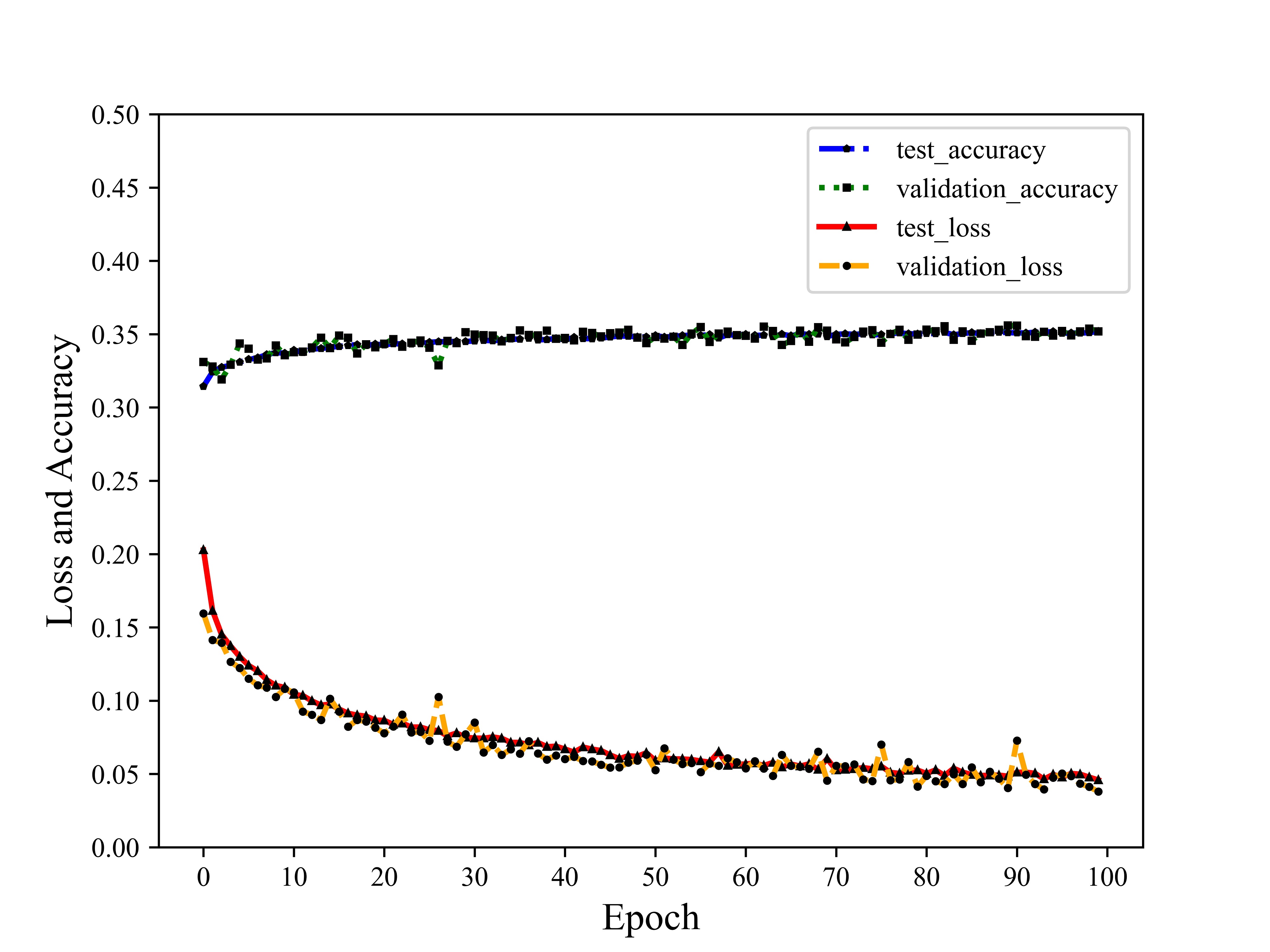

Supplement: Supplemental Information 1 — Model training results and comparison data. [file peerj-cs-10-1915-s001.zip › latex/4.6b.jpg]

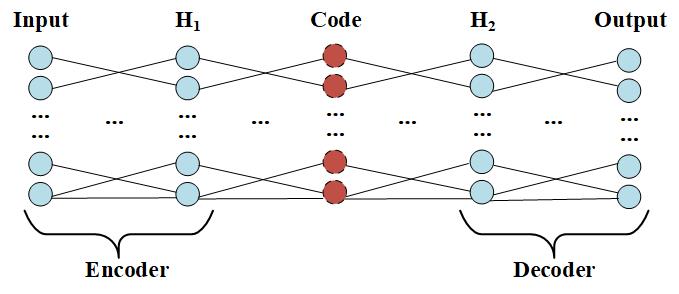

Supplement: Supplemental Information 1 — Model training results and comparison data. [file peerj-cs-10-1915-s001.zip › latex/4.7.jpg]

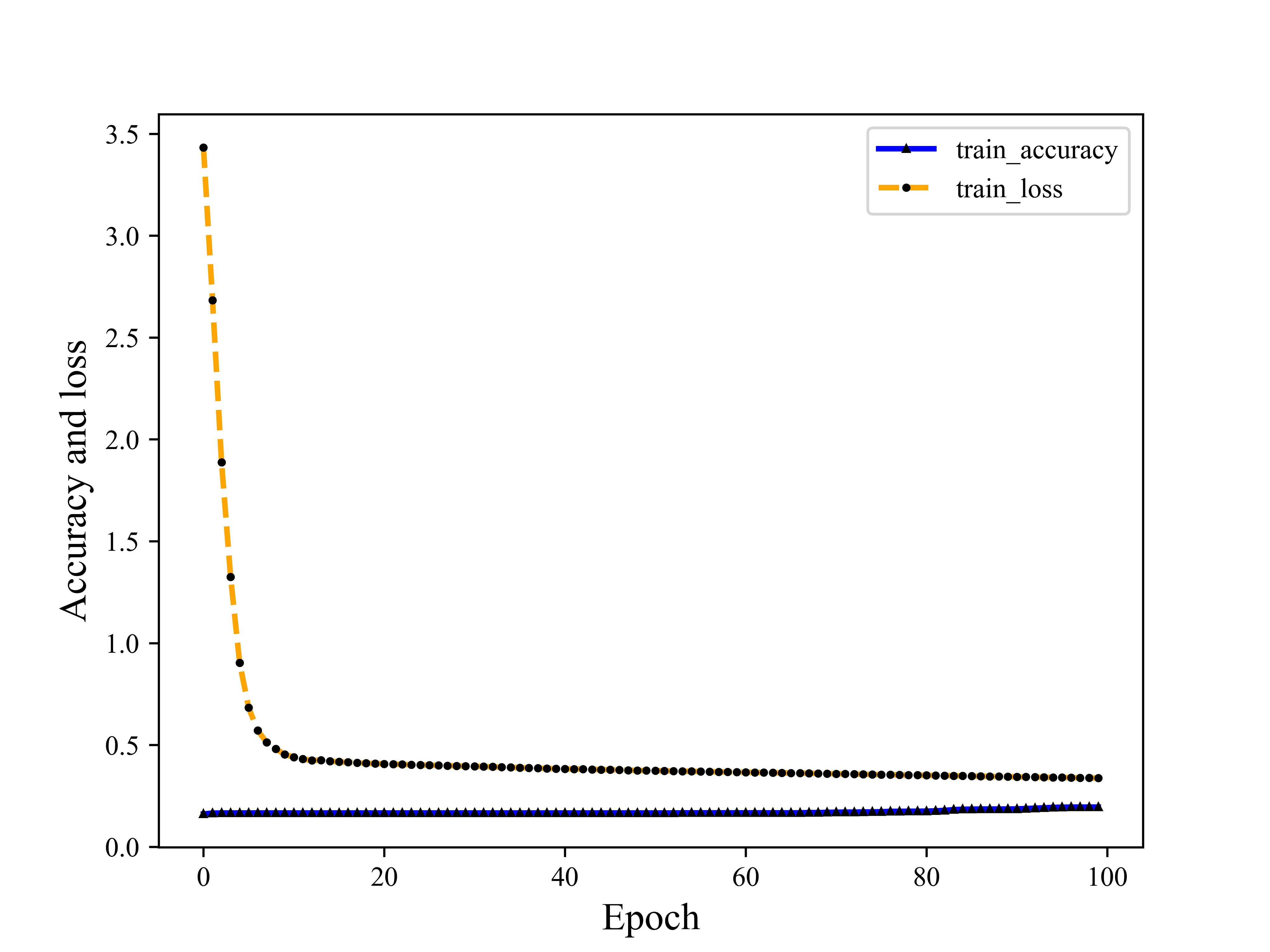

Supplement: Supplemental Information 1 — Model training results and comparison data. [file peerj-cs-10-1915-s001.zip › latex/4.8a.jpg]

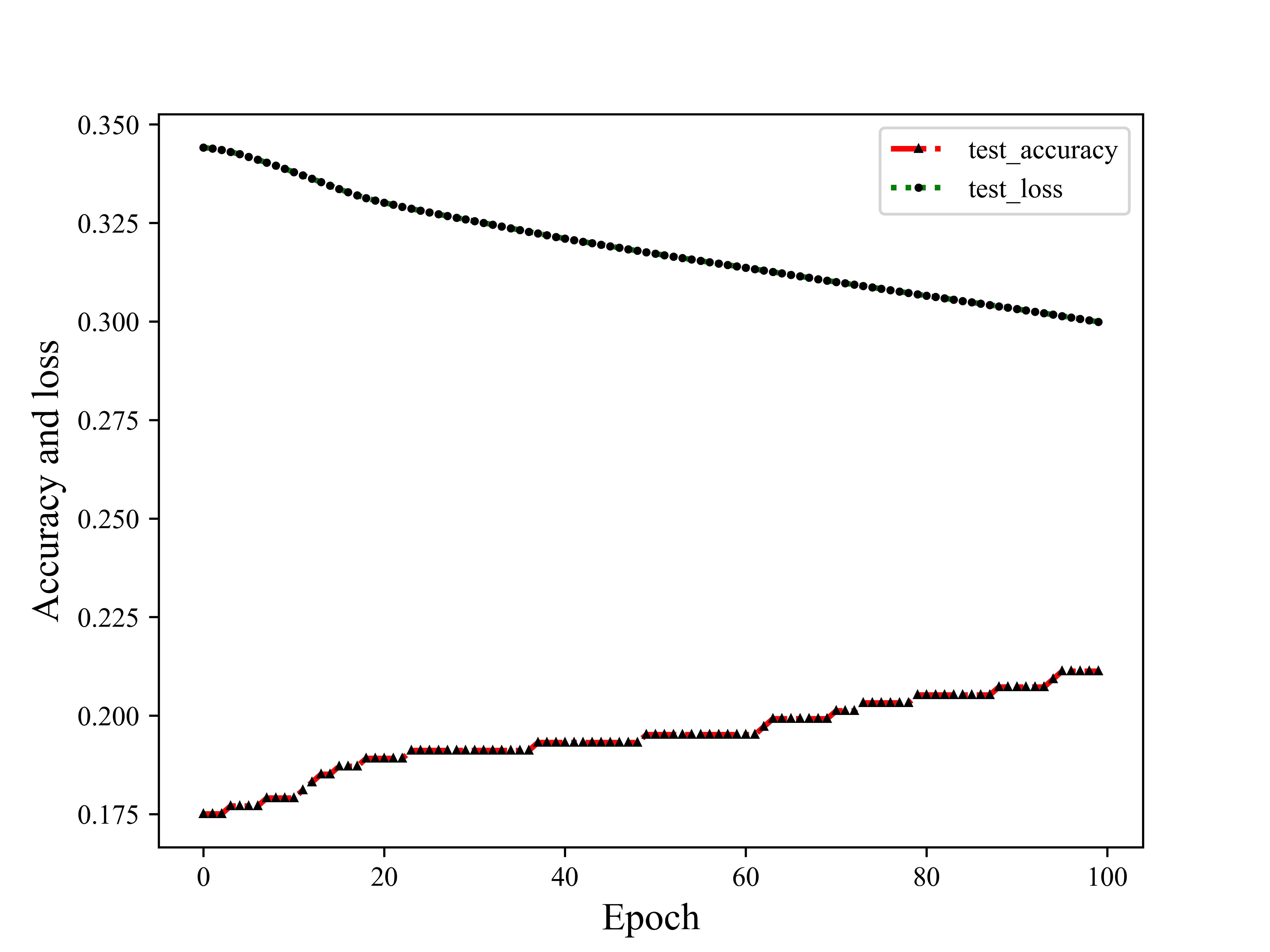

Supplement: Supplemental Information 1 — Model training results and comparison data. [file peerj-cs-10-1915-s001.zip › latex/4.8b.jpg]

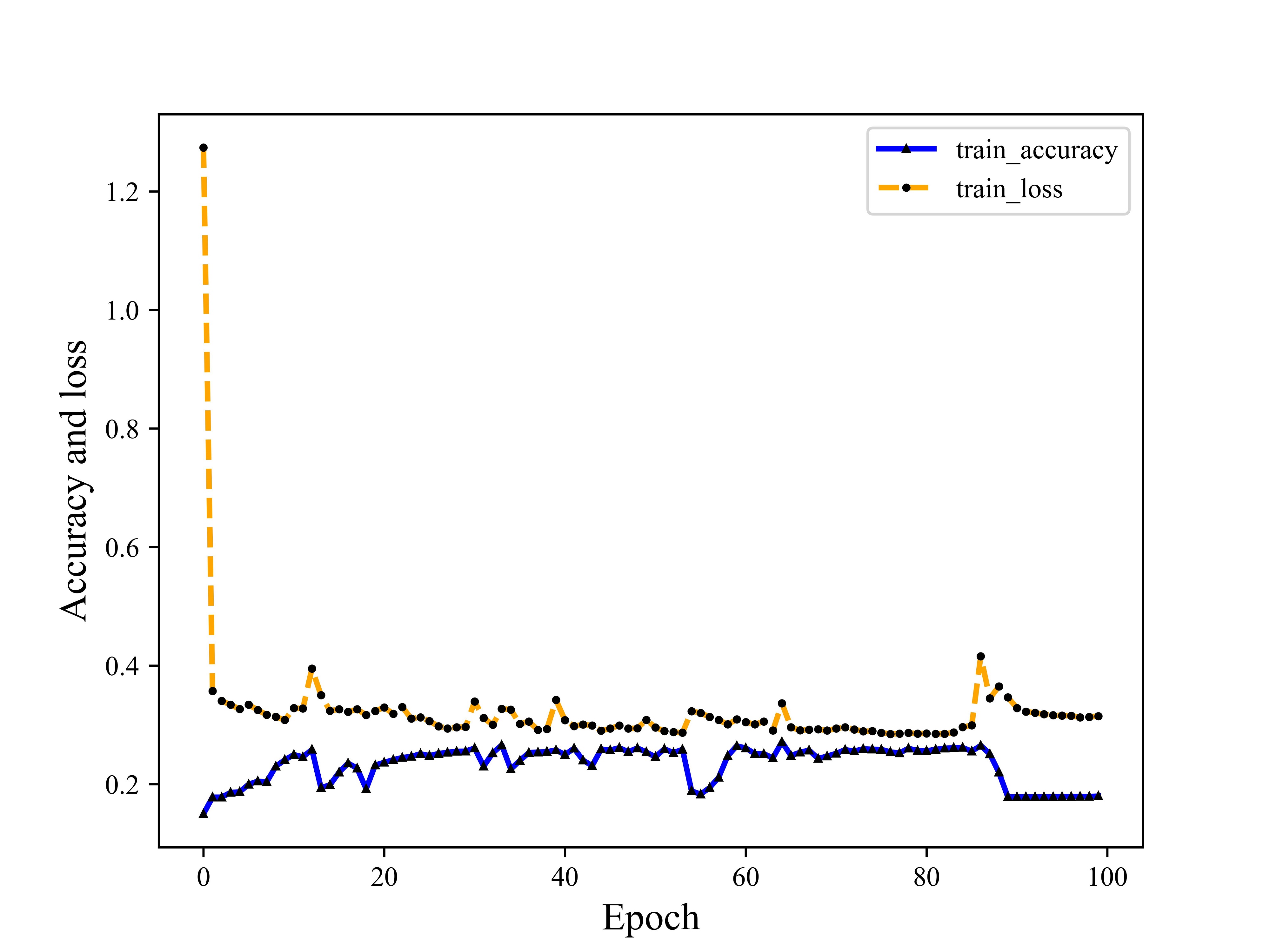

Supplement: Supplemental Information 1 — Model training results and comparison data. [file peerj-cs-10-1915-s001.zip › latex/4.9a.jpg]

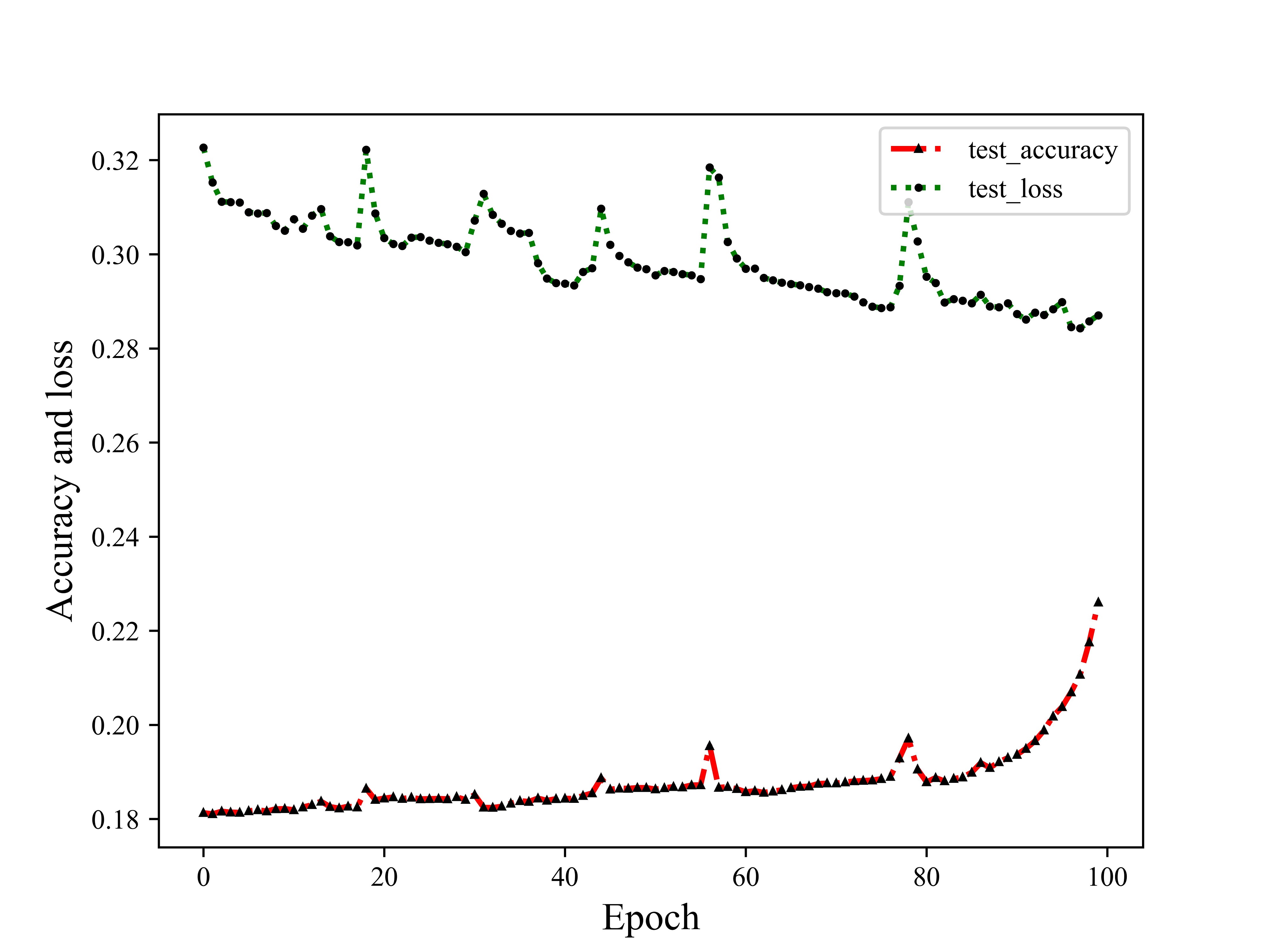

Supplement: Supplemental Information 1 — Model training results and comparison data. [file peerj-cs-10-1915-s001.zip › latex/4.9b.jpg]
